# Immunoinformatics-based design of artificial chimeric proteins as universal vaccine candidates against foot-and-mouth disease virus serotypes A, O, and SAT2

**DOI:** 10.1038/s41598-026-49135-5

**Published:** 2026-06-09

**Authors:** Alyaa Elrashedy, Mohamed Nayel, Akram Salama, Ahmed Zaghawa, Mohamed E. Hasan

**Affiliations:** 1 Department of Animal Medicine and Infectious Diseases (Infectious Diseases), Faculty of Veterinary Medicine, University of Sadat City, Sadat City, Egypt; 2 Bioinformatics Department, Faculty of Biotechnology, University of Sadat City, Sadat City, Egypt; 3 Faculy of Computer and Artificial Intelligence, Al-Ryada University for Science & Technology, Sadat City, 5730306, Egypt

**Keywords:** Foot-and-mouth disease, Protein modeling, Immunoinformatics, Broad-spectrum vaccine, Cross-protection, Molecular docking, Biochemistry, Biotechnology, Computational biology and bioinformatics, Drug discovery, Immunology, Microbiology, Structural biology

## Abstract

Foot-and-mouth disease virus (FMDV) remains a major constraint to livestock health due to its high mutation rate and serotype diversity. Currently, FMDV vaccines, primarily inactivated whole-virus formulations, have significant limitations, including limited cross-protection, high production costs, and potential biosafety risks. To address the need for broad-spectrum protection, this study aimed to design a universal vaccine candidate by rationally constructing artificial chimeric proteins (ACPs) integrating conserved structural (VP1–VP3) and non-structural (3 A, 3 C) proteins from the predominant Egyptian FMDV serotypes A, O, and SAT 2. Three-dimensional modeling via AlphaFold3 and Swiss-Model confirmed the high structural quality of the constructs, with the ACP2 candidate exhibiting superior stability and reliability metrics (TM-score > 0.95, RMSD < 0.5, and overall quality > 88). Functional annotation revealed three conserved domains critical for virion assembly, receptor interaction, and host immune activation. Immunoinformatics analysis identified a robust antigenic profile for ACP1 and ACP2 proteins, comprising (21 and 36) cytotoxic T-lymphocyte (CTL), (18 and 20) helper T-lymphocyte (THL), and (15 and 19) B-cell epitopes prioritized for conservancy and population coverage. Based on these epitopes, three multiepitope vaccine constructs were assembled and analyzed computationally. Molecular docking demonstrated strong and stable binding affinities between the vaccine constructs and bovine TLR9 and TLR4 receptors (lowest binding energies of − 19.4 and − 16.9 kcal/mol, respectively), supported by stable interactions in 100 ns molecular dynamics simulations. These findings highlight the ACP2 construct as a novel, structurally stable, and highly immunogenic candidate capable of eliciting cross-serotype protection. The study provides a translational blueprint for a universal recombinant FMDV vaccine, warranting immediate in vitro expression and in vivo validation.

**Supplementary Information:**

The online version contains supplementary material available at 10.1038/s41598-026-49135-5.

## Introduction

Foot-and-mouth disease virus (FMDV) is considered a highly contagious viral pathogen for cloven-hoofed animals- including cattle, sheep, goats, and pigs- causing severe production losses and trade restrictions^1^. FMDV has distinguished seven serotypes (A, O, C, SAT 1–3, and Asia 1), each exhibiting unique antigenic properties. This diversity poses a major challenge for disease control, as immunity developed against one serotype does not protect against others^2,3^. Furthermore, the virus’s high mutation rate enables it to evade immune responses (IR), increasing the risk of outbreaks even in vaccinated populations^4^. These factors underscore the pressing need for innovative vaccine strategies to address FMDV’s variability and provide universal, long-lasting, cross-serotype protection.

Vaccination remains the cornerstone of FMDV control programs, playing a critical role in minimizing outbreaks and reducing disease severity in infected animals. Current inactivated vaccines are serotype-specific, require frequent boosters, and carry biosafety risks during production, highlighting the need for safer, broadly protective alternatives^5,6^.

Next-generation vaccine strategies focus on subunit and chimeric protein designs, which can safely target conserved antigenic regions across multiple serotypes to generate broad protection^7–9^. Structural proteins (SPs) such as VP1, VP2, and VP3 are pivotal in eliciting immune responses, humoral immunity, as they contain key antigenic sites that stimulate neutralizing antibodies^10–12^. Additionally, non-structural proteins like 3 A and 3 C play vital roles in viral replication and robust cytotoxic T-lymphocyte (CTL) and T-helper lymphocyte (THL) responses, which are critical for cellular immunity^13,14^. Combining these structural and non-structural components into artificial chimeric proteins offers a promising strategy for creating universal vaccine candidates that trigger both arms of immunity.

Bioinformatics plays a vital role in modern vaccine development by leveraging computational tools to analyze complex biological data and predict immune responses^15,16^. It provides the foundation for understanding the structural and functional characteristics of viral proteins, identifying conserved regions, and assessing genetic variability across serotypes, which are essential for designing stable and broadly protective vaccine candidates^17,18^. Immunoinformatics, a specialized field within bioinformatics, focuses specifically on modeling immune recognition through the interaction between antigen and the immune system^19^. Using advanced algorithms to predict B- and T-cell epitopes, identify the protein regions most likely to trigger protective immune responses^20,21^. This targeted approach streamlines vaccine design, avoids allergenic or toxic components, and accelerates development while reducing experimental costs^22^. It also paves the way for innovative strategies, such as chimeric and multivalent vaccine formulations.

This study hypothesized that integrating conserved structural domains with immunogenic non-structural proteins into a single chimeric molecule could overcome the limitations of serotype-specific immunity. Consequently, it aimed to rationally design and validate universal vaccine candidates (ACP1 and ACP2) capable of eliciting broad-spectrum protection against the predominant FMDV serotypes A, O, and SAT 2 in Egypt.

## Materials & methods

**Fig. 1:**
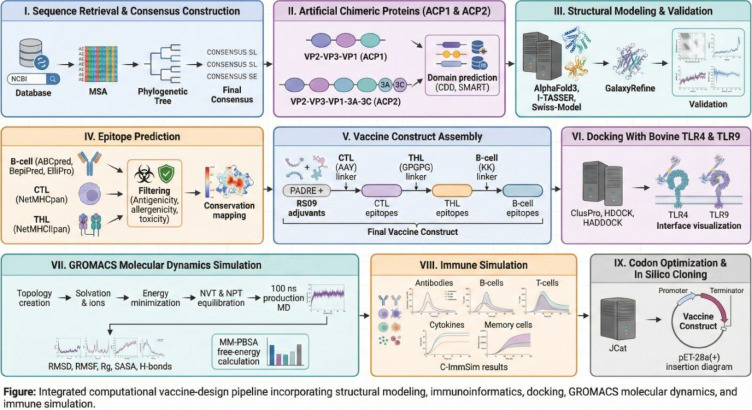
Flowchart of the computational vaccine design pipeline. The workflow integrates target selection, epitope prediction, structural modeling, and immunological simulation across four phases. Key bioinformatics tools utilized for sequence analysis, structure validation, and in silico cloning are listed within the corresponding process boxes.

All major stages were implemented using widely accepted computational approaches supported by recent literature **(**Fig. 1**)**^23^.

### Data retrieval

Amino acid (aa) sequences of five target proteins (VP1, VP2, VP3, 3 A, and 3 C) of the Egyptian target serotypes A, O, and SAT 2 of FMDV were retrieved from the National Center for Biotechnology Information (NCBI) GenBank database (https://www.ncbi.nlm.nih.gov/). The VP1 protein is 213 aa, and the accession numbers of the three serotypes used are in Table S1. The VP2, VP3, 3 A, and 3 C proteins consist of 218, 220, 152, and 212 aa, respectively. These sequences were retrieved from the complete polyprotein sequences of the three FMDV serotypes A, O, and SAT 2, with accession numbers QNN94950, QNN94951, and ARO74646, respectively.

### Multiple sequence alignment, phylogenetic analysis, and consensus sequence

The VP1 protein sequences from each target serotype (A, O, and SAT 2) were first aligned separately with other strains belonging to the same serotype using the CLUSTAL W algorithm^24^ implemented in the BIOEDIT software^25^(https://bioedit.software.informer.com/). Phylogenetic analysis was subsequently performed at the protein level for VP1 sequences from different Egyptian strains using the neighbor-joining approach with the Jones–Taylor–Thornton (JTT) matrix-based model^26^ in the MEGA12 software^27^(https://www.megasoftware.net/). To ensure the robustness of the inferred relationships, a bootstrap analysis method with 1,000 replicates was performed, and nodes with confidence values below 70% were excluded from interpretation^28^. Based on the resulting phylogenetic clusters, one representative VP1 sequence from each clade within a serotype was selected. For each serotype, these representative sequences were aligned to create a serotype-specific consensus sequence. The consensus sequences from the three serotypes were then aligned to generate a final VP1 consensus sequence representing all serotypes.

In contrast, the VP2, VP3, 3 A, and 3 C proteins, which are more conserved across serotypes, were directly aligned among the three serotypes (A, O, and SAT 2) to generate individual consensus sequences for each protein. All consensus sequences were then used in the design of artificial chimeric proteins (ACP1 and ACP2).

### Construction of artificial chimeric proteins

The two artificial chimeric proteins were designed, one encompassing the three SPs (VP1, VP2, and VP3) and the other composed of the three SPs and non-structural proteins (NSPs) (3 A and 3 C), namely ACP1 and ACP2, respectively.

The sequential order of the chimeric protein is VP2 (the Rhv region), VP3 (the rhv-like region), VP1 (complete), 3 A, and 3 C. The final length of the ACP1 and ACP2 proteins was 577 and 943 aa, respectively.

### Domain prediction

Domain separation represents the initial phase in predicting the three-dimensional (3-D) structure of the target proteins. The NCBI Conserved Domain Database (CDD)^24^(https://www.ncbi.nlm.nih.gov/Structure/cdd/cdd.shtml) and SMART (Simple Modular Architecture Research Tool)^25^(http://smart.embl-heidelberg.de/) servers were employed to predict the domains of ACP1 and ACP2. CDD uses Reverse Position-Specific BLAST (RPS-BLAST) to compare query sequences against position-specific scoring matrices (PSSMs) that detect novel domain families and assigns functional annotations to preserved domains. SMART is an extensive analytical server that provides supplementary insights into the protein structure features by predicting domain architectures, motifs, and protein classification.

### Secondary structural & solvent accessibility prediction

To improve the accuracy and reliability of secondary structure and solvent accessibility predictions, eight computational servers were utilized for the ACP1 and ACP2 proteins: RaptorX (http://raptorx.uchicago.edu/)^29^, SOPMA (https://npsa-prabi.ibcp.fr/)^30^, PredictProtein (https://status.predictprotein.org/)^31^, PSIPRED (https://bioinf.cs.ucl.ac.uk/psipred/)^32^, GOR4 (https://npsa-prabi.ibcp.fr/NPSA/npsa_gor4.html)^33^, jpred4 (https://www.compbio.dundee.ac.uk/jpred/)^34^, CFSSP (https://www.biogem.org/tool/chou-fasman/)^35^, and PSSpred (https://aideepmed.com/PSSpred/)^36^.

Using multiple servers improves confidence in the results by generating a robust consensus across different algorithms, as each server employs distinct computational methods and training datasets. This consensus-based approach reduces the likelihood of errors and ensures that the selected predictions reflect the most reliable structural features. The RaptorX server depends on a Deep Convolutional Neural Field (DeepCNF); however, the other servers use neural networks, except PSIPRED, which uses two feed-forward neural networks. The physicochemical properties, transmembrane topology, and signal peptide prediction of the two target proteins were calculated via the ProtParam server^37^(https://web.expasy.org/protparam/) and Phobius prediction server^38^(https://phobius.sbc.su.se/), respectively.

### Three-dimensional structure prediction

The 3-D prediction was performed using different servers employing various approaches: homology modeling (AlphaFold3^39^ (https://alphafold.ebi.ac.uk/), I-TASSER server^40^, Swiss-Model server^41^, fold recognition (LOMETS3 “meta-server”) (https://zhanglab.ccmb.med.umich.edu/LOMETS/), and ab-initio prediction method (Phyre2 server^42^(https://www.sbg.bio.ic.ac.uk/phyre2/html/page.cgi? id=index), I-TASSER, and CEthreader (https://aideepmed.com/CEthreader/)^43^). Employing multiple structure prediction servers allows cross-validation of results, leveraging complementary algorithms to increase reliability and reduce bias from any single method. I-TASSER (https://aideepmed.com/I-TASSER/), recognized as a top-performing platform in the CASP7–CASP14 assessments, constructs full-length atomic models through iterative fragment assembly and simulation processes. Similarly, Swiss-Model (https://swissmodel.expasy.org/) is widely used for homology modeling, supported by an extensive repository of experimentally determined structural templates. AlphaFold, developed by DeepMind and the winner of the CASP 15 competition, achieves near-experimental accuracy in protein structure prediction directly from amino acid sequences.

Combining predictions from multiple servers enhances the accuracy and reliability of the results by strengthening the consensus among them.

### Model refinement

The Galaxy WEB^44^(https://galaxy.seoklab.org/), ReFOLD^45^(https://www.reading.ac.uk/bioinf/ReFOLD/), Modrefiner^46^(https://aideepmed.com/ModRefiner/), and trRossetta^47^(https://yanglab.qd.sdu.edu.cn/trRosetta/) servers were used. These tools aim to improve local and global structural attributes of the primary models, aligning them more closely with their natural state while necessitating minimal computational resources. The refining methodology has two phases: the initial phase enhances the network of the hydrogen bonding (HB), whereas the subsequent phase implements minimization of the energy of the refined model via the integration of physics-based and knowledge-based force fields^46^. All servers and computational tools were used with their default, publicly available parameters. This ensures full reproducibility of our in silico workflow.

### Model evaluation

Protein modeling faces two major challenges: accurate conformational sampling and reliable evaluation of structural models. To address these, advanced large-scale model quality assessment (QA) methods were applied in combination with model clustering to evaluate and rank the predicted structures. The evaluation process for the refined protein models was measured using various criteria (TM-score, RMSD, QMEAN, Z-score, overall quality, clash score, MolProbity score, and Ramachandran Favored (RF)). These scores estimate model quality, including overall fold, distributions of interatomic connections, and dihedral angles. The structure assessment server^48^, TM-Score server^49^(https://aideepmed.com/TM-score/), SAVES meta server^50^(https://saves.mbi.ucla.edu/), QMEAN^51^(https://swissmodel.expasy.org/qmean/), PROCHECK server^52^, and ProSA^53^(https://prosa.services.came.sbg.ac.at/prosa.php) were used to evaluate the prediction of ACP1 and ACP2 proteins. All servers were employed with their default parameters to ensure full reproducibility of this in silico workflow.

### Prediction of functional motif

Motifs and sequence fingerprints play a crucial role in identifying distant relationships between sequences and mediating protein-protein interactions (PPIs), which are essential for understanding protein function. To predict conserved motifs within the ACP1 and ACP2 chimeric proteins, multiple computational tools were employed, including PROSITE^54^, SMART^55^, MotifFinder (https://www.genome.jp/tools/motif/) and Motif Scan (https://www.genome.jp/tools/motif/)^56^. The PROSITE server (https://prosite.expasy.org/) identifies functional motifs and conserved regions by matching query sequences against predefined pattern expressions and profiles. The SMART server utilizes Hidden Markov Models (HMMs) to analyze multiple sequence alignments and detect functionally significant domains. In addition to domain prediction, it provides detailed annotations of coiled-coil regions, transmembrane helices, and signal sequences, thereby offering insights into potential structural and functional roles of the predicted motifs.

### Structural classification

The InterPro database (https://www.ebi.ac.uk/interpro/) was employed to group protein sequences into families and to identify conserved domains and functionally important sites. InterProScan was employed to analyze the ACP1 and ACP2 protein sequences against the comprehensive InterPro signature collection, which integrates predictive models from multiple member databases to define protein families, domains, and conserved motifs with high accuracy, as well as associated gene ontology (GO) terms^57^. The SUPERFAMILY 2.0 database (http://supfam.org) provides information from UniProtKB and NCBI about the domain annotation of millions of protein superfamilies^58^. However, the CATH database (https://www.cathdb.info/) uses wwPDB to identify domains and classify them according to evolutionary superfamilies, providing functional and structural annotations^59^.

The design and in silico validation of the multi-epitope vaccine constructs followed the established, standard pipeline for computational vaccinology. This workflow included (a) identification of immunogenic epitopes, (b) construction of a chimeric sequence with appropriate linkers and adjuvants, (c) 3D modeling and validation, and (d) validation of immunological potential via molecular docking and simulation, as described by^15,22,60,61^.

### Prediction of B-cell epitope

B-cells recognize antigens and produce antibodies. ABCpred (https://webs.iiitd.edu.in/raghava/abcpred/), which utilizes Artificial Neural Networks (ANN), BepiPred 2.0 at the Immune Epitope Database and Analysis Resource (IEDB)^62^(https://services.healthtech.dtu.dk/services/BepiPred-3.0/), ElliPro^63^(https://tools.iedb.org/ellipro/), and SVMTriP^64^(https://sysbio.unl.edu/SVMTriP/) servers were employed for the prediction of linear B-cell epitopes. However, discontinuous B-cell epitopes were predicted using the ElliPro server. These epitopes are associated with protein folding and long-distance separation in the microbial pathogen sequence^65^. By analyzing pathogen sequence attributes using HMMs and amino acid scales, these servers are regarded as highly accurate, demonstrating superior performance and area under the curve (AUC) values^66^. The default parameters of each server were used unless otherwise specified. For ABCpred, a threshold of 0.51 was applied; for BepiPred 2.0, a threshold of 0.5; and for ElliPro, epitopes with a score ≥ 0.5 and a maximum distance of 6 Å were considered for downstream analysis.

### Prediction of T-cell epitope

T-cell epitopes are recognized through two molecules (MHC I and MHC II), established by CD8 + and CD4 + T-cells, respectively. In cattle, the bovine leukocyte antigen (BoLA) complex serves as the counterpart to the human MHC. Upon activation, CD4 + identifies MHC-II, measuring 9 to 22 aa, developing into THL. Similarly, activated CD8 + identifies MHC-I-limited epitopes of 8 to 12 amino acid residues and develops into CTLs. For this analysis, the following servers and Egyptian alleles were utilized: NetMHCpan-4.1^67^ with BoLA-1:01901 and BoLA-2:00801^68^, MHCII-NP^69^, and NetMHCpanII 4.3 (https://services.healthtech.dtu.dk/services/NetMHCIIpan-4.1/)^67^ with BoLA-DRB3*020:02^70^. NetMHCpan-4.1 (https://services.healthtech.dtu.dk/service.php?NetMHCpan-4.1) employs an ANN trained on binding affinity data. The default threshold for strong binding was 1% however, for weak binding is 5%. These thresholds were explicitly chosen to maximize both sensitivity and specificity and are reported here to ensure reproducibility of the analysis.

### Antigenicity, allergenicity, and toxicity prediction

Antigenicity, allergenicity, and toxicity were predicted using VaxiJen v2.0^71^ (http://www.ddg-pharmfac.net/vaxijen/), AllergenFP v.1.0^72^ (https://ddg-pharmfac.net/AllergenFP/), and ToxinPred^73^(https://crdd.osdd.net/raghava/toxinpred/), respectively. The threshold for virus antigenicity was adjusted to 0.5.

### Epitope prioritization and final selection strategy

Following the initial prediction of B-cell, CTL, and THL epitopes, a stringent, multi-step filtration and ranking strategy was applied to select the final set of dominant peptides for inclusion in the chimeric vaccine constructs. This process adhered to the following four hierarchical criteria:

1. Safety Screening (Essential Filter): All predicted epitopes were first screened for safety. Peptides were immediately excluded if they were predicted as allergenic (using AllergenFP v.1.0) or toxic (using ToxinPred). This step eliminated potential adverse reactions in the host.
2. Immunogenic Potential (Antigenicity Threshold): Remaining non-toxic, non-allergenic epitopes were assessed for antigenicity using the VaxiJen v2.0 server. Only peptides scoring above the threshold of 0.5 were retained, ensuring a high likelihood of initiating an immune response.
3. Rationale for Consensus Design: The vaccine utilizes epitopes derived from consensus sequences of the ACP1 and ACP2 proteins to achieve maximal cross-serotype coverage. This process is necessary to integrate the most common and immunodominant residues from genetically diverse serotypes (A, O, SAT 2), resulting in mosaic (synthetic) epitopes that, while optimized, require validation against natural isolates.
4. Conservation Analysis and Stringent Selection: Epitopes that met the safety and antigenicity criteria were then analyzed for conservation across the Egyptian FMDV serotypes (A, O, and SAT 2) using the Conserved-epitope-finder tool^74^. To validate universal potential, a rigorous, two-part conservation analysis was performed on our retrieved dataset (accessions in Table S1). Firstly, the epitopes derived from the VP1 sequence, which contains the most variable neutralizing sites, were screened against the alignment of all retrieved VP1 sequences only. Secondly, the epitopes derived from the highly conserved internal proteins (VP2, VP3, 3 A, and 3 C) were screened against the alignment of retrieved full-length FMDV Polyprotein sequences.
5. Overlapping and Coverage Maximization (Final Selection): The highest-ranking conserved epitopes were then cross-referenced. Peptides identified as overlapping (B-cell and T-cell overlap) were consolidated to reduce the final vaccine length and increase processing efficiency. The final set of selected epitopes was chosen to maximize both the T-cell and B-cell population coverage against the target al.leles (BoLA-I and BoLA-II).

### Construction & validation of the vaccine

Three vaccine constructs were designed based on predicted B- and T-cell epitopes. The first version was derived from the ACP1 protein and included only epitopes with antigenicity scores greater than 1. The second version was based on the ACP2 protein, including all overlapping B- and T-cell epitopes, regardless of antigenicity. The third version was based on the ACP2 protein, like the second version, but included only epitopes with antigenicity scores greater than 1, effectively selecting the most antigenic regions from the second version.

To enhance the immunogenicity of the three constructions, two adjuvants (PADRE and RS09) were attached at the C-terminal end^75^. The PADRE sequence (AKFVAAWTLKAAA) was selected as it is a well-established, potent universal T-helper epitope known to enhance CD4 + T-cell responses^76–78^. The RS09 peptide (APPHALS) was included as it acts as a synthetic agonist for Toll-like receptor 4 (TLR4), powerfully stimulating the innate immune response. Additionally, to ensure optimal antigen processing and presentation and connect the epitopes and adjuvants, specific linkers were employed^22,79–81^. The rigid EAAAK linker was utilized to fuse the adjuvants to the epitope clusters; its alpha-helical structure provides effective domain separation, preventing steric hindrance and ensuring that the adjuvants and epitopes maintain their independent functional conformations^75,82^. AAY linkers were used for CTL epitopes to facilitate proteasomal cleavage^83^, GPGPG linkers for HTL epitopes to prevent junctional immunogenicity and maintain flexibility, and KK linkers for B-cell epitopes to preserve independent immune recognition^84^. This combination is designed to engage both the innate and adaptive immune systems.

Subsequently, the final construct followed this arrangement: CTL-AAY-THL-GPGPG-B-cell-KK-6 H tag-EAAAK-adjuvant-KK-adjuvant^75^. The final constructs evaluated their antigenicity, allergenicity, and toxicity using VaxiJen v2.0, AllergenFP v1.0, and ToxinPred servers, respectively. Physicochemical parameters and solubility were evaluated using ProtParam and protein sol^85^ and SOLpro^86^ servers.

### Secondary, 3-D structure prediction & refinement of the artificial chimeric vaccines

The secondary structures of the designed vaccines were predicted using RaptorX, PredictProtein, and SOPMA servers. The 3-D structure model was then generated using AlphaFold3, I-TASSER, and Swiss-Model servers. Then, refinement of the 3-D vaccine models was performed using the GalaxyRefine server^44^, and model validation was carried out using the Structure Assessment Server, SAVES meta-server, and ProSA tools. To validate the immunological accessibility of the final vaccine construct, discontinuous (conformational) B-cell epitopes were predicted using the ElliPro server. The analysis was performed on the refined 3-D model of the final vaccine constructs to determine if the folding of the chimeric protein maintained the surface exposure of key antigenic determinants. The prediction parameters were set to the default with a minimum score of 0.5 and a maximum distance of 6 Å.

### Disulfide engineering for enhanced stability

To improve the thermostability of the designed vaccine constructs, disulfide engineering was performed using the Disulfide by Design 2.0 (DbD2) server (http://cptweb.cpt.wayne.edu/DbD2/). The refined 3-D models of the vaccine constructs were analyzed to identify residue pairs suitable for mutation to Cysteine. Selection criteria were based on a Chi3 angle range of −87 ° to + 97 ° and a bond energy threshold suitable for stabilizing flexible loop regions without disrupting the protein fold. This approach mimics natural stabilization strategies used in viral capsid engineering^87^.

### Molecular preparation for docking

Before molecular docking, the toll-like receptor structures (bovine TLR9 and TLR4) were retrieved from the AlphaFold database with accession numbers (Q5I2M5 and Q9GL65), respectively. The final designed vaccine constructs underwent rigorous preparation to ensure physically accurate and energetically favorable starting conformations. This process was executed using the Discovery Studio 2021 software (Dassault Systèmes)^88^ and included the following steps:

1. Cleaning and Standardization: The structures were processed to remove all non-essential components, including crystallization water molecules, buffer ions, and cofactors. This was done to focus the docking calculation exclusively on the binding of the vaccine to the TLR receptor interface.
2. Hydrogen Addition and Charge Assignment: Missing hydrogen atoms were added to both the receptor and ligand structures, and necessary atomic charges were assigned according to the software’s default force field.
3. Energy Minimization: To relieve minor structural strain and achieve a stable geometry, the prepared molecules were subjected to a brief round of energy minimization using Discovery Studio’s default parameters. This crucial step ensured the input structures were in an optimized, low-energy conformation before submission to the rigid-body docking servers.

The resulting minimized and optimized receptor and ligand structures were saved as individual PDB files and used as input for the ClusPro 2.0, HDOCK, and HADDOCK servers.

### Molecular docking

Molecular docking was conducted to assess interactions between the constructed vaccines and TLR9 and TLR4, key components of the innate immune system responsible for recognizing pathogen-associated molecular patterns (PAMPs). Docking simulations were conducted using ClusPro 2.0^89^ (https://cluspro.bu.edu/), HDOCK^90^(http://hdock.phys.hust.edu.cn/), and HADDOCK (https://wenmr.science.uu.nl/haddock2.4/)^91^. Three widely recognized platforms employ advanced algorithms for accurate prediction and analysis of PPI. ClusPro 2.0 employs a fast Fourier transform (FFT) algorithm for rigid-body docking, while HDOCK integrates both template-based modeling and docking for improved accuracy. HADDOCK utilizes biochemical and biophysical information to drive the docking process, enhancing the reliability of the predicted interactions. PDBsum (https://www.ebi.ac.uk/thornton-srv/databases/pdbsum/) was also utilized to analyze the complex interactions at the atomic level^92^. Additionally, the PRODIGY server (https://rascar.science.uu.nl/prodigy/) estimates the binding affinity and complex stability, providing valuable insights into the potential efficacy of vaccine constructs to activate TLR9 and TLR4 pathways^93^. These combined approaches allow for a comprehensive understanding of the molecular interactions that may enhance vaccine immunogenicity.

### Molecular dynamics simulation

Biomolecular interactions were simulated using dynamic techniques with the GROMACS software (version 2024.1) and the comprehensive CHARMM27 force field to examine how three vaccine constructs interact with TLR complexes 9 and 4.

Each molecular system was prepared in a cubic computational environment with adequate spacing (10 Å minimum between molecules and boundaries) to ensure proper hydration and minimize computational artifacts. The TIP3P water model was utilized for solution, a standard choice that works well with the selected force field parameters. To create physiologically relevant conditions, Na⁺ and Cl⁻ ions were introduced to achieve a standard salt concentration (0.15 M)^22,94,95^. The preparation protocol included an initial energy optimization phase combining steepest descent and conjugate gradient approaches for 50,000 iterations, which stabilized the molecular configurations. For calculating molecular interactions, the Particle Mesh Ewald technique was employed for electrostatic forces, with a 10 nm cutoff implemented for van der Waals interactions^96^. System equilibration proceeded through two distinct phases (constant NVT followed by constant NPT), each running for 100 ps with 2 fs time increments^97,98^. The main simulation then ran for 100 ns, with structural snapshots preserved every 10 ps for subsequent examination^99,100^. All computational work utilized resources provided by the Bibliotheca Alexandrina Supercomputing Facility and the High Performance Computing services from the Ministry of Higher Education and Scientific Research (Egypt).

The analytical framework included multiple structural parameters: Root Mean Square Deviation (RMSD)to track overall stability, Root Mean Square Fluctuation (RMSF) to identify flexible regions, radius of gyration (Rg) to monitor molecular compactness, hydrogen bonding (HB) patterns, and solvent-accessible surface (SASA) measurements to understand molecular exposure during the simulation period.

### Binding free energy estimation (MM-PBSA)

The binding free energies of the vaccine–receptor complexes were estimated using the Molecular Mechanics Poisson–Boltzmann Surface Area (MM-PBSA) method, implemented through the gmx_MMPBSA package^101^. This approach complements the docking and molecular dynamics analyses by providing a quantitative measure of the interaction strength and stability between each vaccine construct and its respective TLR receptor. MM-PBSA integrates molecular mechanics energy terms with continuum solvation models to generate an accurate representation of the thermodynamic favourability of complex formation. The overall binding free energy (ΔG_bind) was calculated using the expression:

$$\:{\Delta\:}{G}_{\mathrm{bind}}={G}_{\mathrm{complex}}-{G}_{\mathrm{protein}}-{G}_{\mathrm{ligand}}$$

where $\:{G}_{\mathrm{complex}}$denotes the free energy of the assembled receptor–vaccine complex, while $\:{G}_{\mathrm{protein}}$and $\:{G}_{\mathrm{ligand}}$represent the free energies of the isolated receptor and vaccine construct, respectively.

For these calculations, representative frames were extracted from the equilibrated region of each MD trajectory, ensuring that the analysis captured stable, well-relaxed conformations. The gmx_MMPBSA tool was used to compute van der Waals, electrostatic, polar solvation, and non-polar solvation contributions for each snapshot, followed by averaging across all frames to obtain the final ΔG_bind values. These results were used to evaluate the stability of the complexes, identify key energetic contributors, and highlight interaction sites that support strong receptor engagement by the vaccine constructs.

### Immune simulation

The three versions of the constructed vaccines were used to predict the IR in mice using a C-ImmSim tool (https://kraken.iac.rm.cnr.it/C-IMMSIM/), which employs an agent-based model and PSSMs to simulate the interactions between the adaptive immune system and the antigen over time^102^. To ensure the in silico model satisfies the requirement for maximal immunological response and reflects the long-term field application in endemic areas, the simulation protocol used a three-dose regimen. This schedule, consistent with methodologies for assessing prolonged protection^103^, models the necessary primary series and subsequent strategic booster injection.

This simulation predicted the ability of the vaccines to elicit antibody and cytokine responses and evaluated the activity of B and T cells. Simulation parameters configuration was: three injections at time steps 1, 90, and 168; random seed value 12,345; simulation volume 100; and 1100 simulation steps. All other parameters had remained at default settings.

### Codon optimization and in silico cloning

To ensure the translational feasibility of the designed vaccine candidates, an in silico cloning strategy was executed targeting the pET-28a(+) expression vector. First, the amino acid sequences of the three vaccine constructs (Versions 1, 2, and 3) were reverse-translated and codon-optimized for expression in the *Escherichia coli* (strain K12) host system using the Java Codon Adaptation Tool (JCat) server^104^(https://www.jcat.de/). This optimization focused on preventing codon bias and adjusting the GC content and Codon Adaptation Index (CAI) to levels compatible with the prokaryotic host. A restriction analysis was performed to identify compatible cloning sites. The results showed that the codon-optimized gene of interest and the pET-28a(+) plasmid did not share suitable restriction sites for directional cloning. This was addressed by computationally adding specific nucleotide sequences to the gene’s termini: a BamHI restriction site was appended to the N-terminal (5’) end, and an XhoI restriction site was appended to the C-terminal (3’) end. These sequences provided suitable restriction sites for the accurate insertion of the gene into the plasmid’s multiple cloning site (MCS). Subsequently, the optimized nucleotide sequences were electronically cloned into the pET-28a(+) MCS using SnapGene software (free trial) (GSL Biotech LLC) (https://www.snapgene.com/). This facilitates directional insertion downstream of the T7 promoter and N-terminal 6xHis-tag. The final recombinant plasmids were visualized to validate the integrity of the open reading frame (ORF) and ensure the absence of internal restriction sites that could interfere with the cloning process.

## Results

### Phylogenetic analysis and construction of two chimeric proteins

**Fig. 2:**
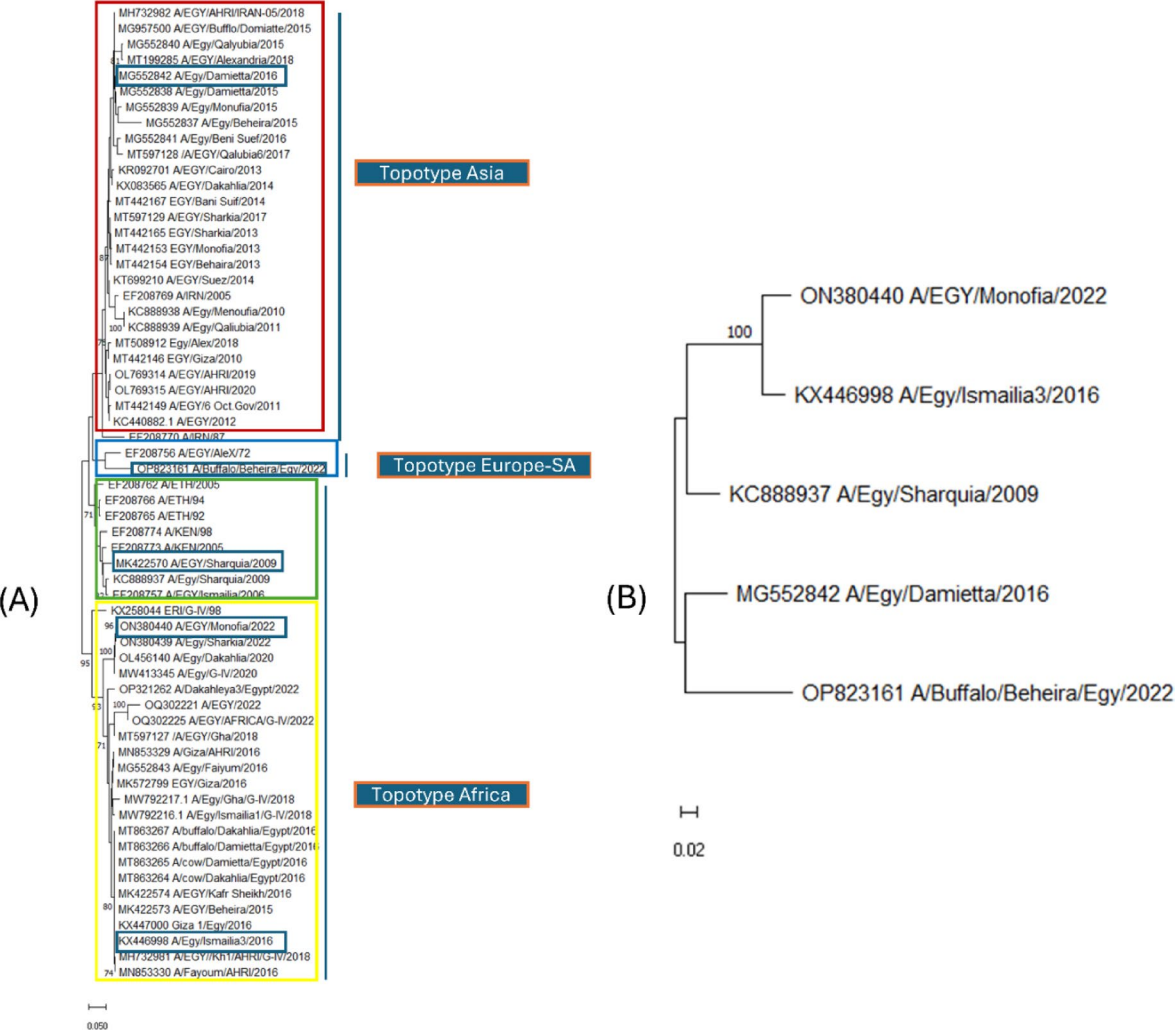
(**A**) The evolutionary relationship was reconstructed through the neighbor-joining technique with the Jones-Taylor-Thornton (JTT) matrix-based model for FMDV’s VP1 protein of serotype A. From the phylogenetic tree, one strain of the VP1 protein was selected from each clade to represent the group. (**B**) The final selected representative strains for each clade were used to create the consensus sequence.

**Fig. 3:**
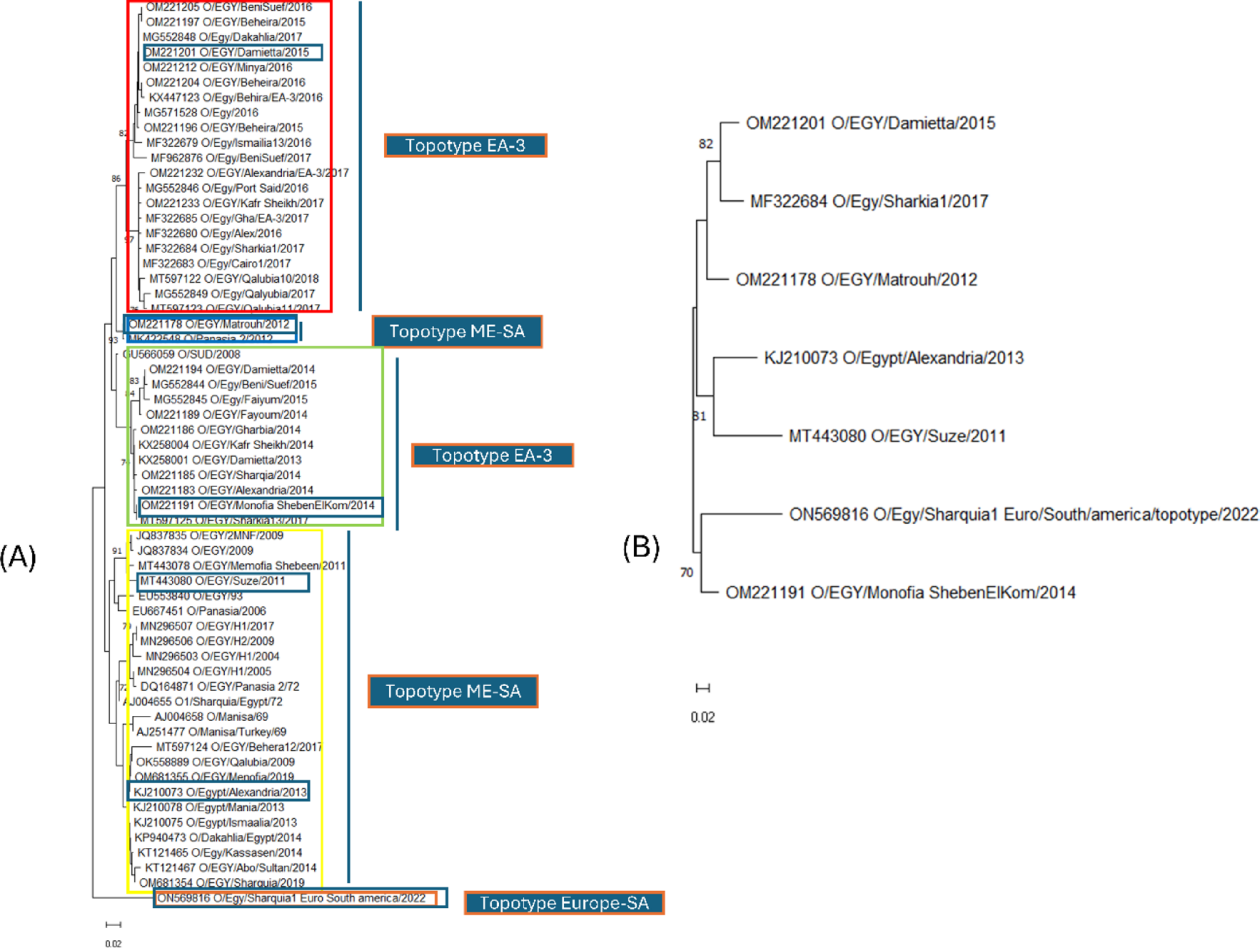
(**A**) The evolutionary relationship was reconstructed through the neighbor-joining technique with the Jones-Taylor-Thornton (JTT) matrix-based model for FMDV’s VP1 protein of serotype O. From the phylogenetic tree, one strain of the VP1 protein was selected from each clade to represent the group. (**B**) The final selected representative strains for each clade were used to create the consensus sequence.

**Fig. 4:**
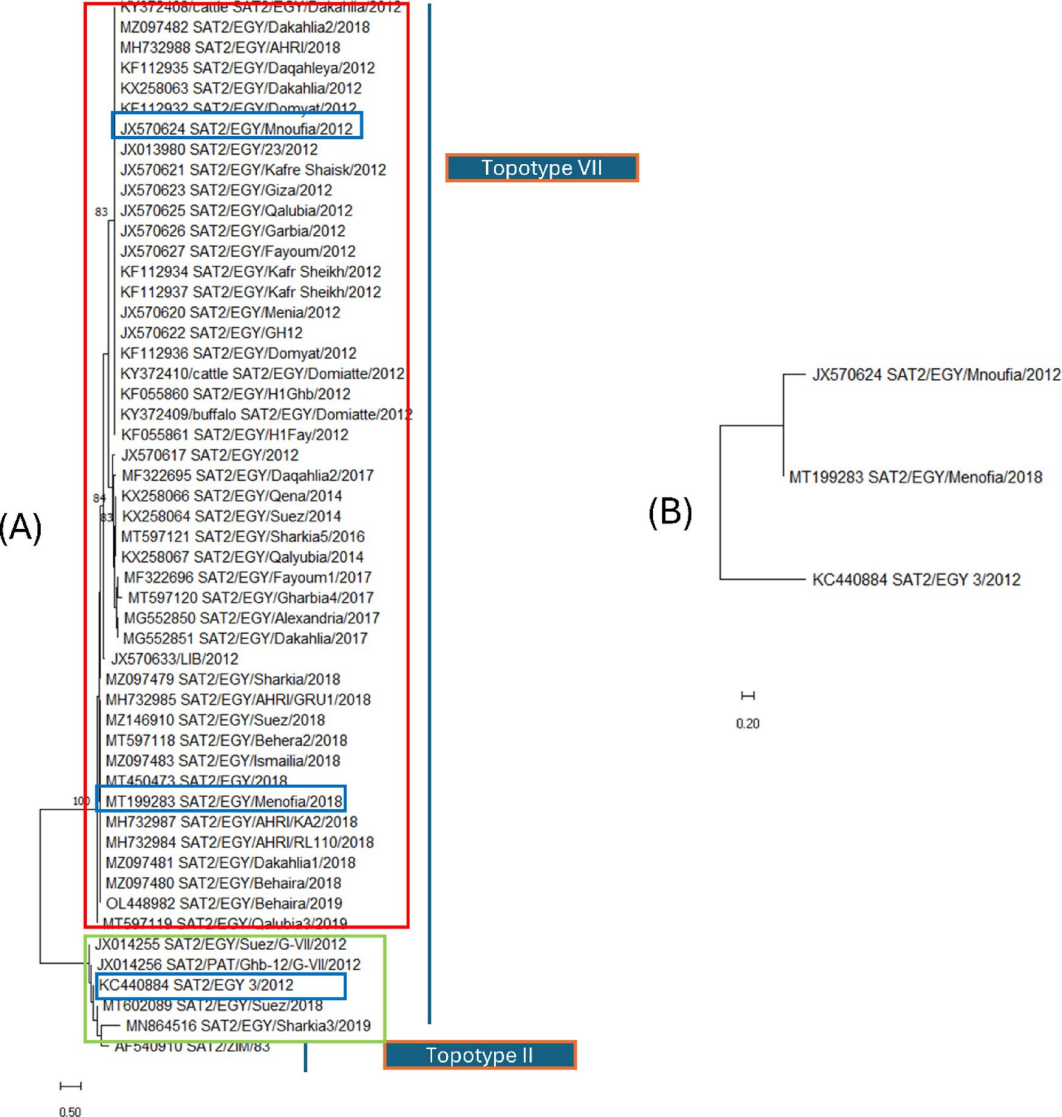
(**A**) The evolutionary relationship was reconstructed through the neighbor-joining technique with the Jones-Taylor-Thornton (JTT) matrix-based model for FMDV’s VP1 protein of serotype SAT 2. From the phylogenetic tree, one strain of the VP1 protein was selected from each clade to represent the group. (**B**) The final selected representative strains for each clade were used to create the consensus sequence.

The phylogenetic trees for serotypes A, O, and SAT 2 in Figs. 2, 3 and 4 illustrate the genetic diversity and clade structures among Egyptian FMDV VP1 protein sequences, confirming the presence of distinct topotypes and clustering patterns. These results were generated using the neighbor-joining approach with the JTT matrix-based model, chosen for its best fit according to the lowest Bayesian Information Criterion (BIC) scores. Each tree clearly separates major topotypes within each serotype, supporting the identification of multiple co-circulating lineages among Egyptian isolates.

For serotype A, the tree distinguishes between Asia, Europe-SA, and Africa topotypes, with clear clustering of geographically and temporally related isolates, facilitating the selection of a representative VP1 strain per clade. In serotype O, several topotypes (EA-3, ME-SA, Europe-SA) are resolved, with strong bootstrap support (> 70%), and representative strains were selected from each major clade, capturing the breadth of observed genetic diversity. SAT 2 demonstrates predominant clustering within topotype VII, as well as its divergence from topotype II clusters, reflecting the unique evolutionary trajectories within this serotype. These representative strains were subsequently used to generate comprehensive consensus sequences for downstream analysis and the design of artificial chimeric proteins, thus integrating the evolutionary and epidemiological insights provided by the protein-level phylogenetic analysis.

Two artificial chimeric proteins were successfully constructed: ACP1, comprising the three SPs (VP1, VP2, and VP3), and ACP2, which includes these three SPs as well as the NSPs (3 A and 3 C). Both constructs follow the same sequential arrangement, VP2 (Rhv region), VP3 (rhv-like region), VP1 (complete), with ACP2 additionally featuring 3 A and 3 C, resulting in final lengths of 577 aa for ACP1 and 943 aa for ACP2.

### Domain prediction

The domains of the two artificial proteins (ACP1 and ACP2) were predicted using the CDD and SMART servers. CDD displayed that the ACP1 protein has two domains, Rhv (1–165 and 166–324) with E-value 8.68e-48 and 2.52e-37, and rhv_like superfamily at position (354–492) with E-value 3.66e-24. The ACP2 protein has an over domain Peptidase_C3 super family at 733–917 with an E-value of 1.50e-65 (Table 1). SMART server revealed that the ACP1 and ACP2 proteins have two and three domains, respectively (Table 2).

The identification of Rhv and rhv-like domains in both ACPs underscores their structural fidelity to the native virion, ensuring the preservation of critical assembly interfaces and receptor-binding topologies necessary for neutralizing antibody induction. Furthermore, the presence of the Peptidase C3 domain in ACP2 suggests the retention of protease-like folding, which is pivotal not only for viral polyprotein processing but also for potentially enhancing intracellular antigen processing and presentation pathways within the host immune system^105–107^.

**Table 1:** Domain prediction of the two chimeric artificial proteins through the CDD server.

Name	Accession	Protein	Position	E-value
Rhv	pfam00073	ACP1 and ACP2	1–165	8.68e-48
			166–324	2.52e-37
rhv_like superfamily	cl13999	ACP1 and ACP2	354–492	3.66e-24
Peptidase_C3 superfamily	cl02893	ACP2	733–917	1.50e-65

**Table 2:** Domain prediction of the two chimeric artificial proteins through the SMART server.

Name	Accession	Protein	Position	E-value	Gene ontology (GO)
Rhv	pfam00073	ACP1 and ACP2	1–165	5.8e-42	GO component: viral capsid (GO:0019028). GO function: structural molecule activity (GO:0005198).
			166–327	2.3e-31	
			354–506	5.8e-18	
Calici_coat domain	PF00915	ACP1 and ACP2	29–174	9e-06	
			179–388	5.9e-07	
Peptidase_C3	PF00548	ACP2	733–917	1.6e-61	GO process: proteolysis (GO:0006508) GO function: cysteine-type endopeptidase activity (GO:0004197)

### Prediction of the secondary structural & solvent accessibility of the ACP1 and ACP2 proteins

To improve the accuracy and reliability of secondary structure and solvent accessibility predictions, eight computational servers were utilized for the ACP1 and ACP2 proteins. While all eight servers provided valuable data, the predictions from the RaptorX server were prioritized for downstream analysis. This server employs an advanced deep learning-based machine, which is widely recognized for its high accuracy in predicting protein structure properties compared to traditional neural network-based methods. The RaptorX server revealed 7% and 13% alpha helix, 34% and 32% beta sheet, 58% and 54% random coil, 27% and 32% exposed, 47% and 41% buried, and 25% membrane for ACP1 and ACP2 proteins, respectively (Table 3).

**Table 3:** Secondary structure & solvent accessibility prediction of the ACP1 and ACP2 proteins.

Protein	Server	Helix (H)	Sheet (E)	Turns (T)/Coils (C)	Buried (b)	Exposed (e)	Membrane (M)
ACP1	SOPMA	12.48%	21.49%	66.03%			
	Predictprotein	6.93%	35.70%	57.37%	59.62%	40.38%	
	RaptorX	**7%**	**34%**	**58%**	**47%**	**27%**	**25%**
	GOR4	20.10%	26.52%	53.38%			
	CFSSP	54.9%	44.5%	9.9%			
	PROTEUS2	7%	40%	53%			
ACP2	SOPMA	25.87%	17.92%	56.20%			
	Predictprotein	14.85%	31.18%	53.93%	55.14%	44.86%	
	RaptorX	**13%**	**32%**	**54%**	**41%**	**32%**	**25%**
	GOR4	29.80%	21.74%	48.46%			
	CFSSP	63.2%	44.8%	11.7%			
	PROTEUS2	15%	34%	51%			

Significant values are given in bold.

The physicochemical properties of the ACP1 and ACP2 proteins were analyzed using the ProtParam tool, revealing favorable characteristics for vaccine development (Table 4). ACP1 consists of 577 amino acids with a molecular weight of 64,189.77 Da, while the larger ACP2 construct contains 943 amino acids with a molecular weight of 104,606.21 Da. Both proteins exhibit a slightly acidic nature, with theoretical isoelectric points (pI) of 6.56 and 6.37, respectively. The constructs were classified as stable, exhibiting instability indices of 25.80 for ACP1 and 27.37 for ACP2, both well below the instability threshold of 40. High aliphatic indices of 78.25 and 80.76 indicate thermostability, while Grand Average of Hydropathicity (GRAVY) values of 0.224 and 0.231 suggest a hydrophobic character. The estimated half-life for both proteins was calculated to be 7.2 h in mammalian reticulocytes (in vitro), > 20 h in yeast, and > 10 h in *Escherichia coli* (in vivo). Furthermore, antigenicity prediction confirmed that both chimeric proteins are antigenic, with VaxiJen scores of 0.5182 for ACP1 and 0.5048 for ACP2, supporting their potential to elicit an immune response.

**Table 4:** Physicochemical features of the ACP1 & ACP2 proteins through the ProtParam tool.

Aspect	ACP1 protein	ACP2 protein
Molecular weight (MW)	64189.77	104606.21
No. of aa	577	943
Formula	C2891H4427N773O848S19	C4671H7285N1265O1387S39
Total no. of negatively charged aa (Glu + Asp)	56	108
Total no. of negatively charged aa (Arg + Lys)	52	99
Theoretical pI	6.56%	6.37%
The estimated half-life	7.2 h (mammalian reticulocytes, in vitro).	
	> 20 h (yeast, in vivo).	
	> 10 h (Escherichia coli, in vivo).	
Grand average of hydropathicity (GRAVY)	0.224	0.231
Aliphatic index	78.25	80.76
Instability index	25.80 (stable)	27.37 (stable)
Antigenicity	0.5182 (Antigenic)	0.5048 (Antigenic)

**Fig. 5:**
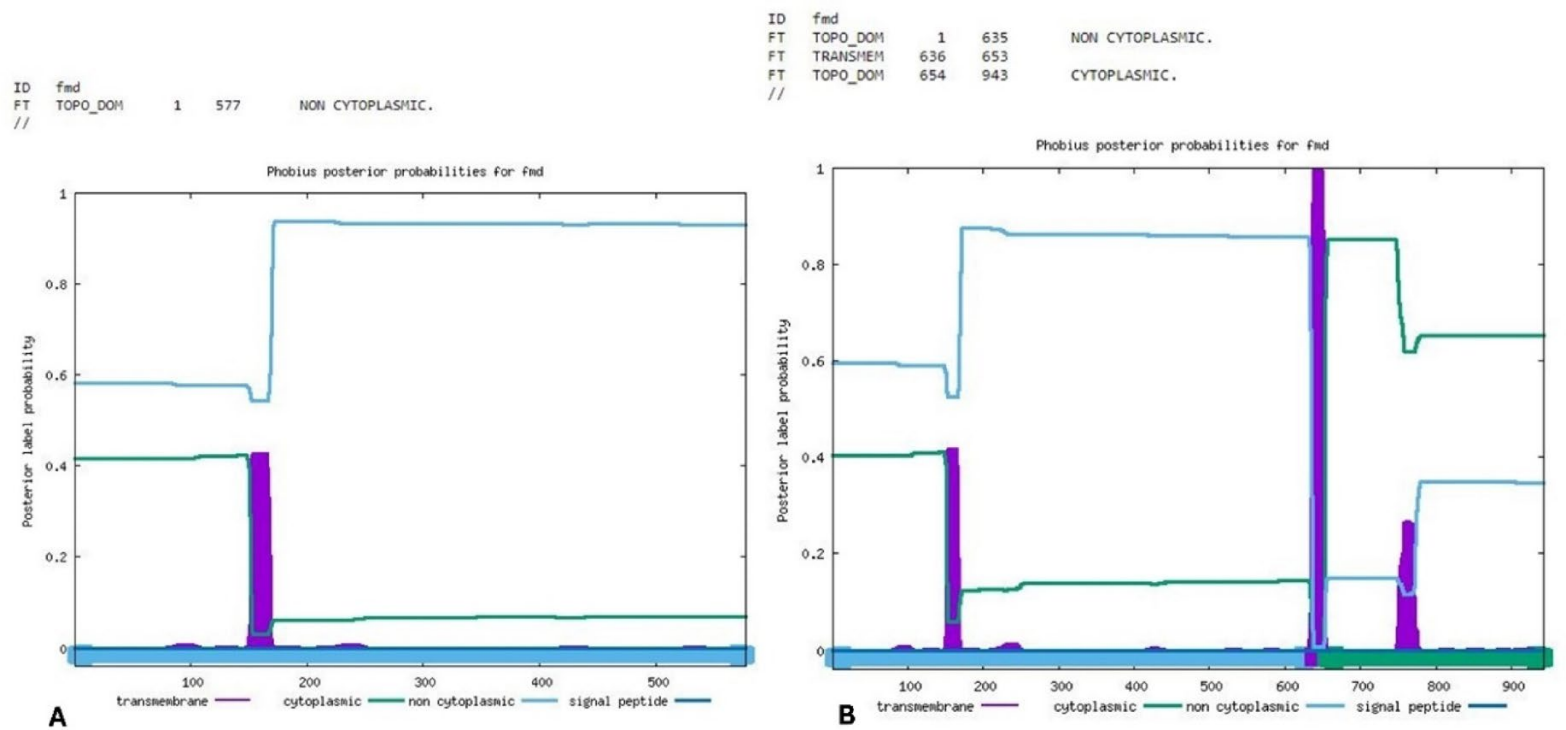
Signal peptide and transmembrane topology prediction of the ACP1 protein (**A**) and ACP2 protein (**B**) using the Phobius prediction server.

Signal peptide and transmembrane topology of the ACP1 and ACP2 proteins were detected using the Phobius prediction server **(**Figs. 4B and 5A**)**, respectively.

### Three-dimensional structure

Construction of the 3-D model passed with three steps: building the initial model, refining, and evaluating. According to accuracy, the best model is chosen (Tables 5 and 6).

ACP1 and ACP2 protein structures were produced using multiple modeling approaches. AlphaFold3, Swiss-Model, LOMETS, and CEthreader servers were employed for regions with reliable target-template alignments, while Phyre2 and I-TASSER were used for unaligned or low-similarity regions. Based on confidence score (C-score) with its typical range (−5, 2) and protein length, the best models from I-TASSER were − 2.21 and − 1.27 in C-score, 0.45 ± 0.15 and 0.56 ± 0.15 in TM-score, and 13.0 ± 4.2Å and 11.9 ± 4.4Å in RMSD for ACP1 and ACP2 proteins, respectively. Swiss-Model provided one and two models for ACP1 and ACP2 proteins with QMEAN scores (0.60 and 0.80), respectively.

In the second phase of model structure prediction, the initial models of ACP1 and ACP2 were refined using GalaxyWEB, ModRefiner, ReFOLD, and trRosetta. Several validation servers were used to evaluate the refined predicted structures of the two target proteins, including the Structure Assessment Server, TM-score, SAVES, QMEAN, PROCHECK, and ProSA servers.

The selected refined models exhibited TM-scores above 0.5, indicating high structural accuracy. TM-scores between 0.17 and 0.5 suggest models with reasonably accurate topology, regardless of protein size^108^. The QMEANDisCo score estimates the absolute quality of a model by comparing it to reference structures solved via X-ray crystallography. It is based on the QMEAN term, with all components integrated using neural networks trained to predict per-residue lDDT scores within the range [0,1]^48^.

**Fig. 6:**
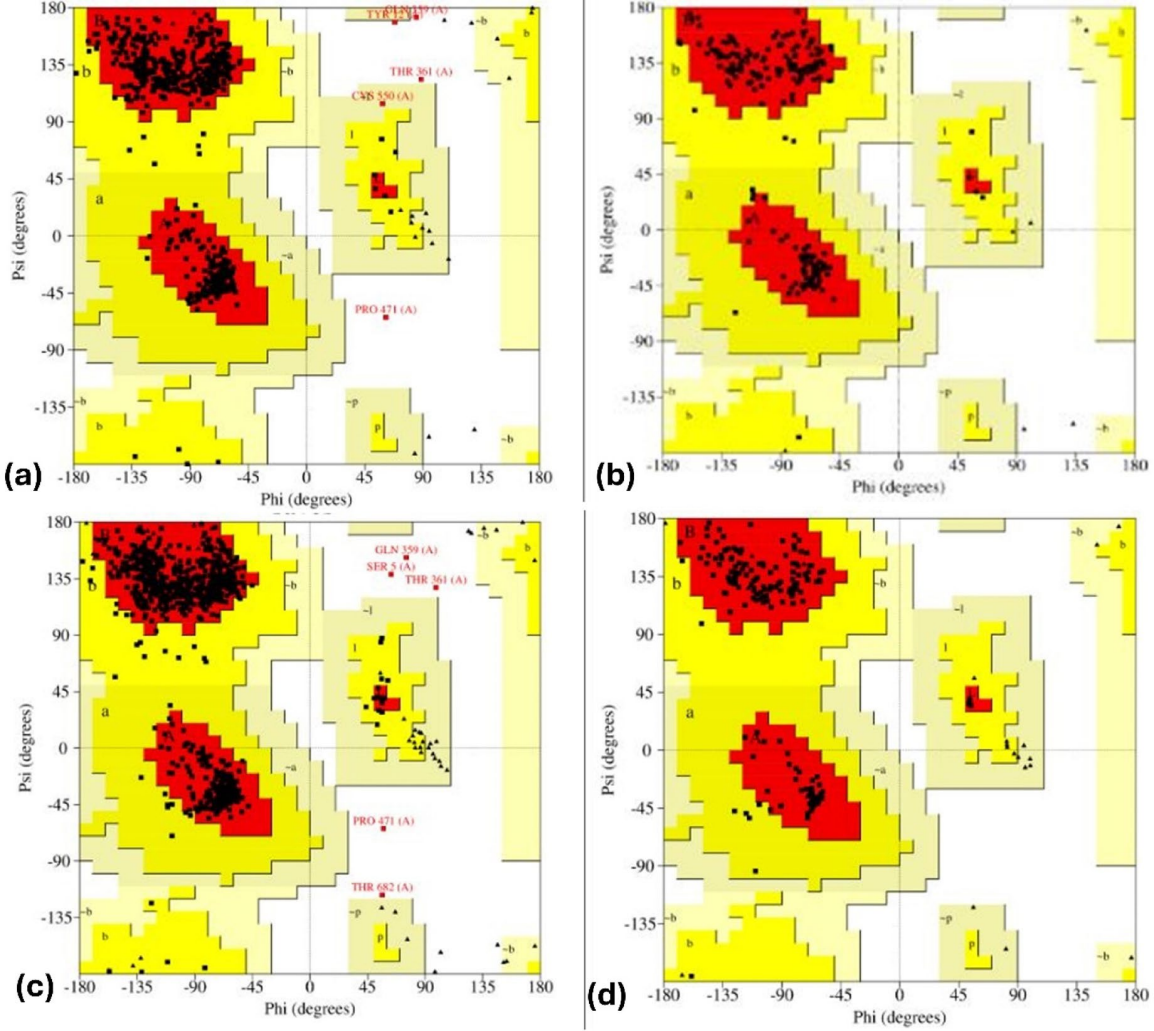
The Ramachandran plot of the ACP1 protein from AlphaFold3 (**a**) and Swiss Model (**b**), and ACP2 from AlphaFold3 (**c**) and Swiss Model (**d**).

Models from AlphaFold3 and Swiss Model servers were the best. For the ACP1 protein, the best models generated by AlphaFold3 and Swiss-Model exhibited RMSD values of 0.33 and 0.47, Z-scores of − 9.0 and − 3.7, TM-scores of 0.9740 and 0.9334, QMEANDisco values of 0.64 ± 0.05 and 0.71 ± 0.06, and ERRAT scores of 87.35 and 88.00, respectively. Similarly, for the ACP2 protein, AlphaFold3 and Swiss-Model produced the most reliable models, with RMSD values of 0.47 and 0.29, Z-scores of − 9.43 and − 6.09, TM-scores of 0.9510 and 0.9864, QMEANDisco values of 0.62 ± 0.05 and 0.87 ± 0.06, and ERRAT scores of 88.84 and 88.82, respectively **(**Fig. 6a–d**)**. Ramachandran plot assessment further confirmed that most residues in both proteins occupied energetically favorable regions, supporting model accuracy. All models have been refined through the GalaxyWeb server, which provided the best-refined structures based on the overall quality metrics (Tables 5 and 6).

**Table 5:** Evaluation of the refined initial 3-D structure of ACP1 protein through different servers based on CASP15.

		RMSD	TM-score	Z-score	QMEANDisco	Overall quality	MolProbity score	Clash score	Ramachandran favored
Modrefiner	Alphafold3 colab	1.154	0.9843		0.62 ± 0.05	67.6732	2.58	56.97	94.96%
	CEthreader	2.816	0.9649		0.33 ± 0.05	34.9177	3.40	91.92	89.04%
	I-TASSER	1.771	0.9747		0.26 ± 0.05	38.5586	3.12	88.72	82.61%
	LOMETS	2.876	0.9754		0.45 ± 0.05	37.2051	3.02	69.92	88.52%
	Phyre2	0.464	0.9925		0.73 ± 0.06	69.8718	2.41	45.74	96.81%
	SWISS-MODEL	0.454	0.9932		0.68 ± 0.06	69.8718	2.14	31.77	97.17%
Galaxyweb	Alphafold3 colab	0.391	0.9653	−8.94	0.64 ± 0.05	85.2126	1.86	11.50	95.83%
	CEthreader	0.496	0.9194	−3.96	0.33 ± 0.05	45.057	2.843	39.5	89.5%
	I-TASSER	0.490	0.9281	−5.09	0.27 ± 0.05	62.2222	2.37	15.30	87.48%
	LOMETS	0.423	0.9497	−8.2	0.46 ± 0.05	69.4757	2.13	11.50	89.91%
	Phyre2	0.470	0.9408	−4.25	0.76 ± 0.06	87.4126	1.43	3.98	96.28%
	SWISS-MODEL	0.475	0.9334	−3.7	0.71 ± 0.06	88	1.22	3.56	97.64%
trRosetta	Alphafold3 colab	1.36	0.853	-	0.60 ± 0.05	83.6812	1.48	2.57	93.22%
	I-TASSER	1.43	0.893	-	0.60 ± 0.05	84.8485	1.52	3.69	94.78%
	LOMETS	1.41	0.839	-	0.59 ± 0.05	78.6642	1.55	3.57	94.09%
	Phyre2	1.95	0.787	-	0.69 ± 0.06	65.8228	0.83	0.00	95.21%
	SWISS-MODEL	2.23	0.792	-	0.67 ± 0.06	75.2874	1.44	2.97	94.81%
Refold	Alphafold3 colab	1.42	0.8872	−8.22	0.64 ± 0.05	72.0562	2.54	31.64	94.26%
	I-TASSER	3.44	0.1524	−4.72	0.27 ± 0.05	74.498	2.27	1.56	79.83%
	LOMETS	2.95	0.4706	−8.51	0.47 ± 0.05	72.007	3.06	2.95	88.35%
	Phyre2 (partial)	1.02	0.9448	−4.38	0.76 ± 0.06	75	2.14	14.93	96.24%
	SWISS-MODEL (partial)	0.87	0.9606	−3.92	0.70 ± 0.06	80.402	2.76	16.35	94.34%

**Table 6:** Evaluation of the refined initial 3-D structure of ACP2 protein using different servers based on CASP15.

		RMSD	TM-score	Z-Score	QMEANDisco	Overall quality	MolProbity score	Clash score	Ramachandran favored
Modrefiner	Alphafold3 colab	0.800	0.9941	-	0.29 ± 0.05	29.2159	3.67	130.32	76.94%
	I-TASSER	0.9874	1.208	-	0.22 ± 0.05	49.944	3.36	87.12	79.60%
	LOMETS	1.639	0.9854	-	0.61 ± 0.05	69.2951	2.53	45.09	94.05%
	Phyre2	0.396	0.9945	-	0.79 ± 0.06	80.6452	2.15	46.01	97.97%
	SWISS-MODEL	0.353	0.9956	-	0.85 ± 0.06	84.2105	2.18	39.83	97.50%
Galaxyweb	Alphafold3 colab	0.472	0.9510	−9.43	0.62 ± 0.05	88.8373	1.553	10.7	98%
	I-TASSER	0.9271	0.467	−5.8	0.23 ± 0.05	66.706	2.24	12.64	86.7
	LOMETS	0.475	0.9303	−9.12	0.29 ± 0.05	39.333	2.988	37.9	79.5%
	Phyre2	0.334	0.9774	−5.99	0.81 ± 0.06	97.8495	1.840	17.1	97.5%
	SWISS-MODEL	0.290	0.9864	−6.09	0.87 ± 0.06	88.8197	1.502	9.5	99%
trRosetta	Alphafold3 colab	3.28	0.509	−10.1	0.49 ± 0.05	85.5962	1.80	5.60	91.82%
	I-TASSER	2.80	0.474	−9.8	0.50 ± 0.05	77.4744	1.91	7.04	90.86%
	LOMETS	2.78	0.506	−6.4	0.50 ± 0.05	83.871	1.68	4.03	91.92%
	Phyre2	2.97	0.979	−6.25	0.83 ± 0.06	95.4802	0.72	0.66	97.97%
	SWISS-MODEL	3.59	0.953	−6.36	0.81 ± 0.06	96.8085	0.94	0.65	96.50%
Refold	Alphafold3 colab	3.76	0.1566	−9.53	0.62 ± 0.05	79.8013	2.40	18.53	93.94%
	I-TASSER	3.47	0.3459	−5.46	0.23 ± 0.05	79.6774	3.49	29.73	83.63%
	LOMETS	3.26	0.1464	−9.31	0.30 ± 0.05	78.626	3.00	13.94	79.60%
	Phyre2	2.73	0.0628	−4.87	0.74 ± 0.06	84.4444	2.45	15.95	94.62%
	SWISS-MODEL	2.99	0.0732	−5.62	0.85 ± 0.06	93.299	2.04	14.25	97%

### Motif prediction

**Fig. 7:**
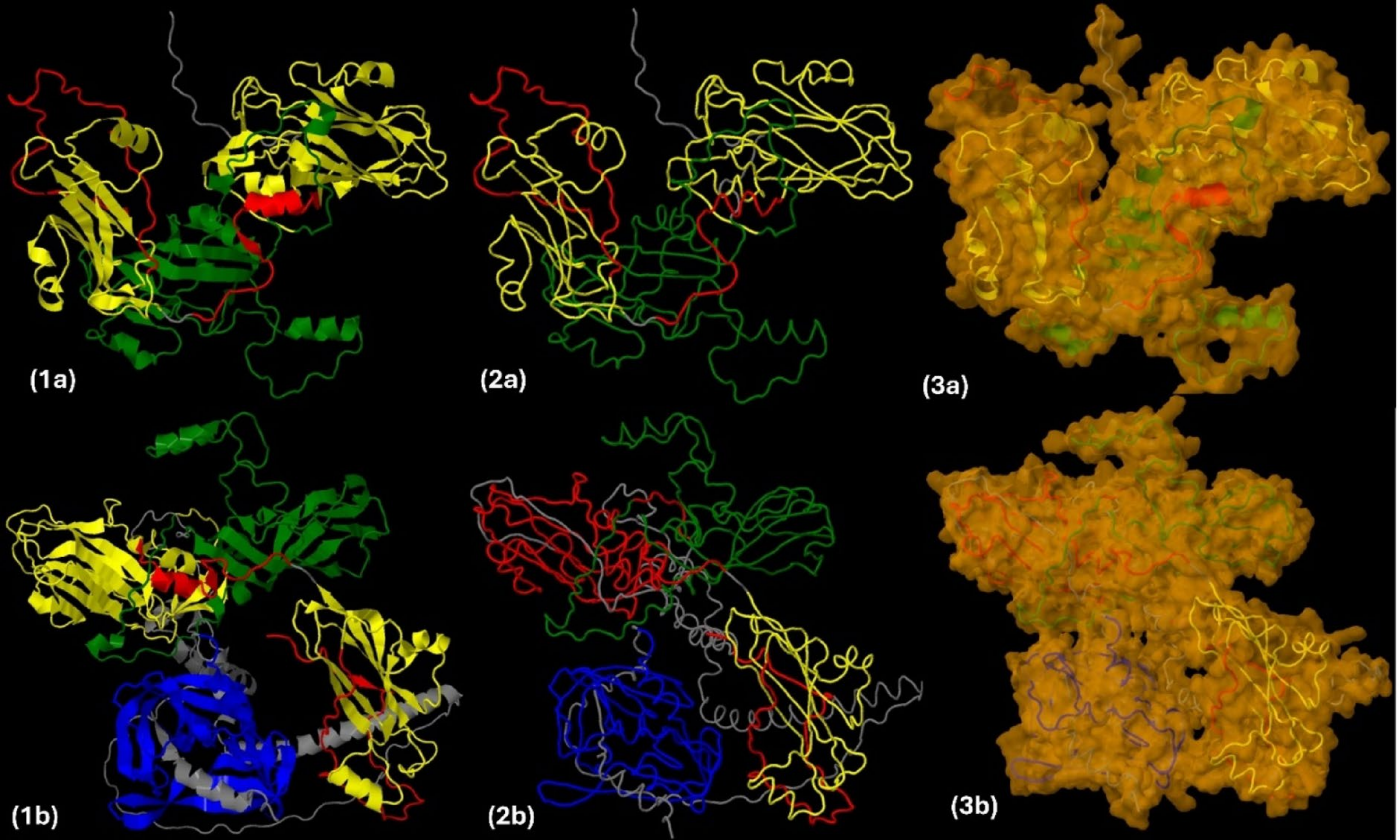
The predicted three-dimensional structures of ACP1 (**a**) and ACP2 (**b**). Panels (1**a**–1**b**) show cartoon representations highlighting the major structural motifs, Rhv (red), Rhv_5 (green), Calici_coat (yellow), and in ACP2 the additional Peptidase_C3 domain (blue), with remaining regions in gray. Panels (2**a**–2**b**) present the refined backbone models, and panels (3**a**–3**b**) display the solvent-accessible surface representations.

Four servers: PROSITE, SMART, MotifFinder, and Motif Scan were applied to determine the PPI, PTMs, and motif function relationship of the ACP1 and ACP2 proteins (Tables 7 and 8). The ACP1 protein has revealed three motifs via the MotifFinder server, including Rhv (PF00073, 1.164, 168.324, and 356.498), Rhv_5 (PF22663, 363.553), and Calici_coat (PF00915, 45.165 and 191.362). However, the ACP2 protein has an over motif Peptidase_C3 (PF00548, 733.917) **(**Fig. 7**)**.

Post-translational modification (PTM) analysis identified several functional motifs in both ACP1 and ACP2 proteins using the Motif Scan server. These include N-glycosylation sites (ASN_GLYCOSYLATION), which contribute to protein folding and stability. These sites identified may influence antigen uptake by dendritic cells and protect the peptide backbone from premature proteolytic degradation, thereby prolonging the immune response. cAMP- and cGMP-dependent protein kinase phosphorylation sites (CAMP_PHOSPHO_SITE) and casein kinase II phosphorylation sites (CK2_PHOSPHO_SITE), which are involved in signal transduction and cellular regulation; and N-myristoylation sites (MYRISTYL), which facilitate membrane association. Additionally, both proteins contain protein kinase C phosphorylation sites (PKC_PHOSPHO_SITE) and tyrosine kinase phosphorylation sites (TYR_PHOSPHO_SITE), which play essential roles in activation and immune signaling, as well as an RGD (arginine–glycine–aspartic acid) cell attachment sequence, indicating potential interaction with integrin receptors that influence cell adhesion and immune responses. Notably, the phosphatase tensin-type domain (PPASE_TENSIN) and the pentatricopeptide repeat (PPR) domain were detected only in the ACP2 protein, suggesting additional structural or regulatory functions unique to this construct.

**Table 7:** Motif prediction of the two target artificial proteins through the motiffinder server.

Category	Protein	Name	Signature	Matching position	E-value
RNA Associated Protein Motif Posttranslational Modifications	ACP1	Rhv	PF00073, picornavirus capsid protein	1.164	4.6e-43
				168.324	2.5e-37
				356.498	5.9e-22
		Rhv_5	PF22663, Picornavirus coat protein	363.553	4.1e-34
		Calici_coat	PF00915, Calicivirus coat protein	45.165	1.4e-05
				191.362	7.2e-07
	ACP2	Peptidase_C3	PF00548, 3 C cysteine protease (picornain 3 C)	733.917	7.5e-59

**Table 8:** Post-translation modification site prediction of the two target artificial proteins through the motif scan server.

Signature	Position
N-glycosylation site. ASN_GLYCOSYLATION	23–26, 129–132, 292–295, 404–407, 460–463, and 519–522
cAMP- and cGMP-dependent protein kinase phosphorylation site. CAMP_PHOSPHO_SITE	386–389 and **840–843**
Casein kinase II phosphorylation site. CK2_PHOSPHO_SITE	14–17, 25–28, 107–110, 203–206, 275–278, 303–306, 310–313, 362–365, 372–375, **610–613**,** 677–680**,** 682–685**,** 793–796**,** and 865–868**
N-myristoylation site. MYRISTYL	7–12, 89–94, 266–271, 290–295, 443–448, 452–457, 529–534, **594–599**,** 732–737**,** 765–770**,** 847–852**,** 895–900**,** 909–914**,** and 915–920**
Protein kinase C phosphorylation site. PKC_PHOSPHO_SITE	47–49, 107–109, 403–405, 561–563, **582–584**,** 610–612**,** 656–658**,** 793–795**,** 836–838**,** and 865–867**
Cell attachment sequence. RGD	505–507
Tyrosine kinase phosphorylation site. TYR_PHOSPHO_SITE	69–76, 535–541, and **790–797**
Phosphatase tensin-type domain profile. PPASE_TENSIN	**369–597**
Pentatricopeptide (PPR) repeat profile. PPR	**579–613**

The bold is referring to the post-translational modification that is over in the ACP 2 protein.

### Structural classification for the two target artificial proteins

The ACP1 protein exhibited two hits located at 1–169 and 174–556, which are classified as positive-stranded ssRNA viruses superfamily and *Picornaviridae*-like VP (VP1, VP2, VP3, and VP4) family. Furthermore, the ACP2 protein showed the same hits with over one at 737–933, which were categorized in Trypsin-like serine proteases superfamily and Viral cysteine protease of trypsin-fold family using the superfamily server (Table 9).

Moreover, the CATH server was employed for the classification of the ACP1 and ACP2 proteins. They were Mainly Beta (2) in class, Jelly Rolls (2.60.120) in topology, Sandwich (2.60) in architecture, and 2.60.120.20 in homologous superfamily (Table 10).

**Table 9:** Structural classification for two target chimeric artificial proteins through the superfamily server.

Domain	Protein	Region	Superfamily	Family	E-value
1	ACP1 and ACP2	1–169	Positive-stranded ssRNA viruses	Picornaviridae-like VP (VP1, VP2, VP3 and VP4)	3.79e-07
2		174–556			3.79e-07
3	ACP2	737–933	Trypsin-like serine proteases	Viral cysteine protease of trypsin-fold	1.18e-54

**Table 10:** Structural classification for two target chimeric artificial proteins through the CATH server.

Level	Description	Match	E-value
Class	Mainly Beta (2)	Genome polyprotein (2.60.120.20/FF/12)	7.6e-108
Architecture	Sandwich (2.60)	2.60.120.20/FF/22	2.0e-26
Topology	Jelly Rolls (2.60.120)	Genome polyprotein (2.60.120.20/FF/11)	2.0e-33
Homologous superfamily	2.60.120.20	Capsid protein VP1 (2.60.120.20/FF/8)	4.5e-8

### Epitope prediction

The combined analysis from SVMTriP, ABCpred, BepiPred 2.0, and ElliPro revealed 3, 12, 4, and 3 predicted linear epitopes, respectively, for the ACP1 protein (Table S2). A similar pattern was observed for the ACP2 protein, with more than four linear epitopes located within NSP regions (Table S3). These predicted epitopes provide valuable insight into potential immunogenic regions that may contribute to effective antibody recognition in vaccine design.

The ElliPro server predicted discontinuous B-cell epitopes comprising 314 and 108 residues for the ACP1 protein, with average scores of 0.708 and 0.74 based on the AlphaFold3 and Swiss-Model structures, respectively. Similarly, for the ACP2 protein, the AlphaFold3 and Swiss-Model structures exhibited 491 and 84 residues, with corresponding average scores of 0.76 and 0.73.

Additionally, the Prediction of MHC-I was performed using NetMHCpan-4.1 with BoLA-1:01901 and BoLA-2:00801 (Table S4). In contrast, the MHCⅡ was predicted using MHCII-NP and NetMHCpanII-4.3 servers with BoLA-DRB3*020:02 (Table S5). The antigenic potential of the epitopes was evaluated using VaxiJen v2.0, while their allergenicity and toxicity were assessed through the AllergenFP and ToxinPred servers, respectively. Only epitopes with antigenicity scores above 0.5 and classified as non-allergenic were selected for further analysis. For MHCI epitope prediction, BoLA-1:01901 and BoLA-2:00801 Egyptian alleles were used. Through the NetMHCpan-4.1 server, the ACP1 and ACP2 proteins exhibited 51 and 66 CTL epitopes, respectively (Tables S4 and S5).

Additionally, for the prediction of MHCII cell epitope, MHCII-NP and NetMHCpanII-4.3 servers were employed with the Egyptian allele BoLA-DRB3*020:02. MHCII-NP and NetMHCpanII servers revealed 1 and 32 THL epitopes of ACP1 protein, respectively (Table S6), and the same in the ACP2 protein, with two THL epitopes increased: YDKIMLDGR and FAYRAATKA peptide sequences at positions (777–791 and 878–892) with antigenicity 0.5249 and 0.8314, respectively.

The integrated results from all B- and T-cell epitope prediction servers were filtered based on the selection criteria defined in the methods (antigenicity > 0.5, non-allergenic, and non-toxic). The conservation analysis, performed against the sequences of all 172 retrieved strains, revealed the compositional status of the 75 prioritized epitopes. This analysis confirmed that 31 epitopes were conserved as exact matches across the entire nature sequences, validating their stability. Notably, 44 epitopes were categorized as synthetic, meaning they were unique mosaic sequences derived from the consensus process and did not exist as exact matches in the natural dataset. These synthetic peptides exhibit only 1–2 aa substitutions across a majority of the circulating serotypes. They were deliberately retained because they mapped to hypervariable yet immunodominant regions and conferred superior population coverage when mapped against the bovine MHC alleles. The final set of 75 dominant epitopes selected for vaccine assembly is summarized in (Table 11), with the full conservation status for each peptide detailed in (Table S7).

This filtering revealed a total of 15 and 19 dominant B-cell epitopes, 21 and 36 dominant CTL epitopes, and 18 and 20 dominant THL epitopes for the ACP1 and ACP2 proteins, respectively (Table 11).

**Table 11:** The final overlapping epitopes from B and T cells through the different servers used.

Epitopes	ACP1		ACP2		label
	Position	Sequence	Position	Sequence	
B- Cell	6–15	VGVTYGYA	6–15	VGVTYGYA	conserved
	105–126	LCSLKDREEYQLTLFPHQFINP	105–126	LCSLKDREEYQLTLFP HQFINP	synthetic
	147–162	YKRHKPWTLVVMVVTP	147–162	YKRHKPWTLVVMVVTP	synthetic
	241–260	TINLHFMFTGPTDHKARYMV	241–260	TINLHFMFTGPTDHKARYMV	synthetic
	261–276	AYVPPGVEVGQPPRTP	261–276	AYVPPGVEVGQPPRTP	synthetic
	277–292	EDAAHCIHAEWDTGLN	277–292	EDAAHCIHAEWDTGLN	synthetic
	297–318	FSIPYISAADYAYTASDTAETT	297–318	FSIPYISAADYAYTASDTAETT	synthetic
	308–321	AYTASDTAETTNVQ	308–321	AYTASDTAETTNVQ	conserved
	322–337	GWVCVLQVTDTHSAEA	322–337	GWVCVLQVTDTHSAEA	synthetic
	338–341	AVVVSVSAG	338–341	AVVVSVSAG	conserved
	342–357	SVSAGPDLEFRFPIDP	342–357	SVSAGPDLEFRFPIDP	conserved
	381–396	TQVQRRKHTDVAFILD	381–396	TQVQRRKHTDVAFILD	synthetic
	407–422	SFVVDLMQTREHALVG	407–422	SFVVDLMQTREHALVG	synthetic
	446–465	LTWVPNGAPETALLNTTNPT	446–465	LTWVPNGAPETALLNTTNPT	synthetic
	507–532	DLAALAAKYADTNHTLPPSFNYGAIT	507–532	DLAALAAKYADTNHTLPPSFNYGAIT	synthetic
			650–665	VIMIRETRKRQQMVDD	synthetic
			698–713	TVGFRERTLPGQKADD	synthetic
			801–816	EFEIKVKGQDMLSDAA	conserved
			923–938	CVSRSMLLKMKAHIDP	conserved
CTL	8–16	VTYGYATAE	8–16	VTYGYATAE	conserved
	22–30	PNTSGLETR	22–30	PNTSGLETR	conserved
	65–73	GVYGKLTDS	65–73	GVYGKLTDS	synthetic
	105–113	LCSLKDREE	105–113	LCSLKDREE	synthetic
	134–142	ITVPYLGVN	134–142	ITVPYLGVN	synthetic
	152–160	PWTLVVMVV	152–160	PWTLVVMVV	conserved
	160–168	VTPLTVVYN	160–168	VTPLTVVYN	synthetic
	182–190	LDVAEACPT	182–190	LDVAEACPT	conserved
	191–199	FLCFDDGKP	191–199	FLCFDDGKP	synthetic
	214–222	FDVSLAAKH	214–222	FDVSLAAKH	synthetic
	240–248	GTINLHFMF	240–248	GTINLHFMF	conserved
	262–270	YVPPGVEVG	262–270	YVPPGVEVG	synthetic
	291–299	LNSSFTFSI	291–299	LNSSFTFSI	synthetic
	347–355	PDLEFRFPI	347–355	PDLEFRFPI	conserved
	355–363	IDPVQTTSA	355–363	IDPVQTTSA	synthetic
	383–391	VQRRKHTDV	383–391	VQRRKHTDV	synthetic
	416–424	REHALVGAL	416–424	REHALVGAL	synthetic
	444–452	NRLTWVPNG	444–452	NRLTWVPNG	synthetic
	509–517	AALAAKYAD	509–517	AALAAKYAD	conserved
	526–534	FNYGAITAT	526–534	FNYGAITAT	synthetic
	543–551	MKRAELYCP	543–551	MKRAELYCP	conserved
			581–589	PSQKSVLYF	conserved
			594–609	GQHEAAIEF	conserved
			610–618	SLKEELRPL	synthetic
			636–644	FEIVALCLT	conserved
			655–670	ETRKRQQMV	synthetic
			682–697	TLDEAEKNP	conserved
			759–767	AICCATGVF	conserved
			792–800	ITDRDYRVF	synthetic
			818–826	MVLHRGNRV	conserved
			837–852	ARMKKGTPV	conserved
			865–873	TYKDIVVCM	conserved
			876–884	DTMPGLFAY	conserved
			884–892	YRAATKAGY	conserved
			927–935	SMLLKMKAH	conserved
			934–949	AHIDPEPHH	conserved
THL	73–87	TNMTAHITVPYLGVN	73–87	TNMTAHITVPYLGVN	synthetic
	113–136	EYQLTLFPHQFINPRTNMTAHITV	113–136	EYQLTLFPHQFINPRTNMTAHITV	synthetic
	148–162	KRHKPWTLVVMVVTP	148–162	KRHKPWTLVVMVVTP	synthetic
	172–186	NNYPGRFTNLLDVAE	172–186	NNYPGRFTNLLDVAE	synthetic
	211–225	LATFDVSLAAKHMSN	211–225	LATFDVSLAAKHMSN	synthetic
	234–248	YYTQYSGTINLHFMF	234–248	YYTQYSGTINLHFMF	conserved
	234–254	YYTQYSGTINLHFMFTGPTDH	234–254	YYTQYSGTINLHFMFTGPTDH	synthetic
	255–269	KARYMVAYVPPGVEV	255–269	KARYMVAYVPPGVEV	synthetic
	279–293	AAHCIHAEWDTGLNS	279–293	AAHCIHAEWDTGLNS	conserved
	296–310	TFSIPYISAADYAYT	296–310	TFSIPYISAADYAYT	synthetic
	302–318	ISAADYAYTASDTAETT	302–318	ISAADYAYTASDTAETT	synthetic
	337–351	AAVVVSVSAGPDLEF	337–351	AAVVVSVSAGPDLEF	conserved
	352–365	RFPIDPVQTTSAGE	352–365	RFPIDPVQTTSAGE	synthetic
	387–401	KHTDVAFILDRFVKV	387–401	KHTDVAFILDRFVKV	synthetic
	414–458	QTREHALVGALLRAATYYFCDLEIAVVHD GNRLTWVPNGAPETAL	414–458	QTREHALVGALLRAATYYFCDLEIAVVHD GNRLTWVPNGAPETAL	synthetic
	505–519	RGDLAALAAKYADTN	505–519	RGDLAALAAKYADTN	synthetic
	523–548	PPSFNYGAITATKPVELLYRMKRAEL	523–548	PPSFNYGAITATKPVELLYRMKRAEL	synthetic
	553–567	PLLAAYKHTDRRHKQ	553–567	PLLAAYKHTDRRHKQ	synthetic
			777–791	YDKIMLDGR	conserved
			878–892	FAYRAATKA	conserved

### Vaccine construction & validation

The universal artificial construct vaccine against FMDV was designed using the final overlapping epitopes identified from various prediction servers, linked through appropriate peptide linkers. Three vaccine versions were developed based on the artificial proteins and their antigenicity profiles.

The first version depends on the ACP1 protein and selected epitopes with antigenicity higher than one. It consists of sixteen CTL epitopes connected by AAY linkers, three THL epitopes joined by GPGPG linkers, and four B-cell epitopes linked by KK linkers (Figure S1). The final construct consisted of 368 amino acids, had an antigenicity score of 0.9654, and was predicted to be non-allergenic and non-toxic.

The second version relies on the ACP2 protein and selects all antigenic epitopes. It consists of thirty-one CTL epitopes connected by AAY linkers, five THL epitopes linked by GPGPG linkers, and eight B-cell epitopes joined via KK linkers (Figure S2). The complete vaccine sequence was 648 amino acids long, with an antigenicity score of 0.8390, and was also predicted to be non-allergenic and non-toxic.

The third version is based on the ACP2 protein and selected epitopes with antigenicity higher than one. It consists of twenty-two CTL epitopes linked by AAY linkers, three THL epitopes connected by GPGPG linkers, and six B-cell epitopes joined by KK linkers (Figure S3). This construction was 504 amino acids long, exhibited an antigenicity score of 0.9202, and was determined to be non-allergenic and non-toxic. The physicochemical features of all three constructed vaccine versions were subsequently analyzed using the ProtParam tool (Table 12).

**Table 12:** Physicochemical features of the three versions of the constructed vaccine using the ProtParam tool.

Aspect	Version 1	Version 2	Version 3
MW	39386.65	70557.84	54537.03
No. of aa	368	648	504
Formula	C1806H2714N474O508S6	C3207H4903N855O897S24	C2487H3773N659O703S12
Total no. of negatively charged residues (Asp + Glu)	30	58	48
Total no. of positively charged residues (Arg + Lys)	36	79	57
Theoretical pI	8.90	9.24	8.93
The estimated half-life	100 h (mammalian reticulocytes, in vitro).		
	> 20 h (yeast, in vivo).		
	> 10 h (*Escherichia coli*, in vivo).		
Grand average of hydropathicity (GRAVY)	−0.091	−0.171	−0.183
Instability index	19.55 (Stable)	25.48 (Stable)	21.41 (Stable)
Aliphatic index	74.84	73.33	73.67
Antigenicity	0.9687 (Antigenic)	0.8371 (Antigenic)	0.9232 (Antigenic)
Allergenicity	Non allergic	Non allergic	Non allergic

### Secondary structure prediction

The secondary structure of the three versions of the constructed vaccines was predicted using the RaptorX, PredictProtein, and SOPMA servers. It consists of alpha helix (13%, 24%, and 15%), beta-sheet (25%, 23%, and 27%), random coil (60%, 51%, and 57%), exposed residues (38%, 40%, and 43%), buried amino acid (37%, 33%, and 33%), and intermediate (23%, 25%, and 22%) for the three versions of the artificial chimeric vaccines, respectively.

### 3-D structure prediction, refinement, & validation of the three constructed vaccines

The initial 3D models of the three vaccine constructs (Versions 1, 2, and 3) were generated using AlphaFold3, Swiss-Model, and I-TASSER servers **(**Fig. 81A, 2A, and 3A**)**. To enhance their stereochemical quality and relax local structural clashes, the initial models were subjected to refinement using the GalaxyRefine server. AlphaFold3 displayed the most accurate models for the constructs, which were subsequently refined using the GalaxyRefine tool. A comparative quality assessment was performed to quantify the improvement pre- and post-refinement.

Initially, the unrefined models for Versions 1, 2, and 3 displayed ERRAT quality factors of 59.19, 65.89, and 55.29, respectively. The refined models exhibited TM-scores of 0.8451, 0.6674, and 0.8388, RMSD values of 0.693, 1.212, and 0.717, MolProbity scores of 1.334, 1.397, and 0.915, and clash scores of 2.9, 1.5, and 0.8, respectively **(**Fig. 81B, 2B, and 3B**)**. Additionally, Ramachandran plot analyses indicated 96.2%, 91.3%, and 97% of aa in favored regions, validating the stereochemical quality of the models. The corresponding QMEANDisco values were 0.24, 0.24, and 0.21, with ERRAT scores of 76.1928, 98.6428, and 71.5278 (Table 13), and Z-scores of − 2.97, − 2.97, and − 2.54 **(**Fig. 81C, 2C, and 3C**)**. Overall, these results confirm that these refined models possess accurate folding, stable geometry, and high structural reliability, supporting their suitability for further molecular docking and immunoinformatics analyses.

To verify that the selected B-cell epitopes retained their antigenicity in the context of the final folded vaccines, discontinuous epitope prediction was performed on the refined 3-D model of the three versions of the constructed vaccine using the ElliPro server. The analysis revealed (188, 317, and 252) distinct conformational B-cell epitopes with scores ranging from (9.206, 26.098, and 17.428) (Tables S8-S10). Crucially, the structural mapping confirmed that the high-affinity B-cell epitopes were located on the solvent-accessible surface loops of the construct vaccines rather than buried, ensuring their availability for efficient interaction with B-cell receptors.

**Fig. 8:**
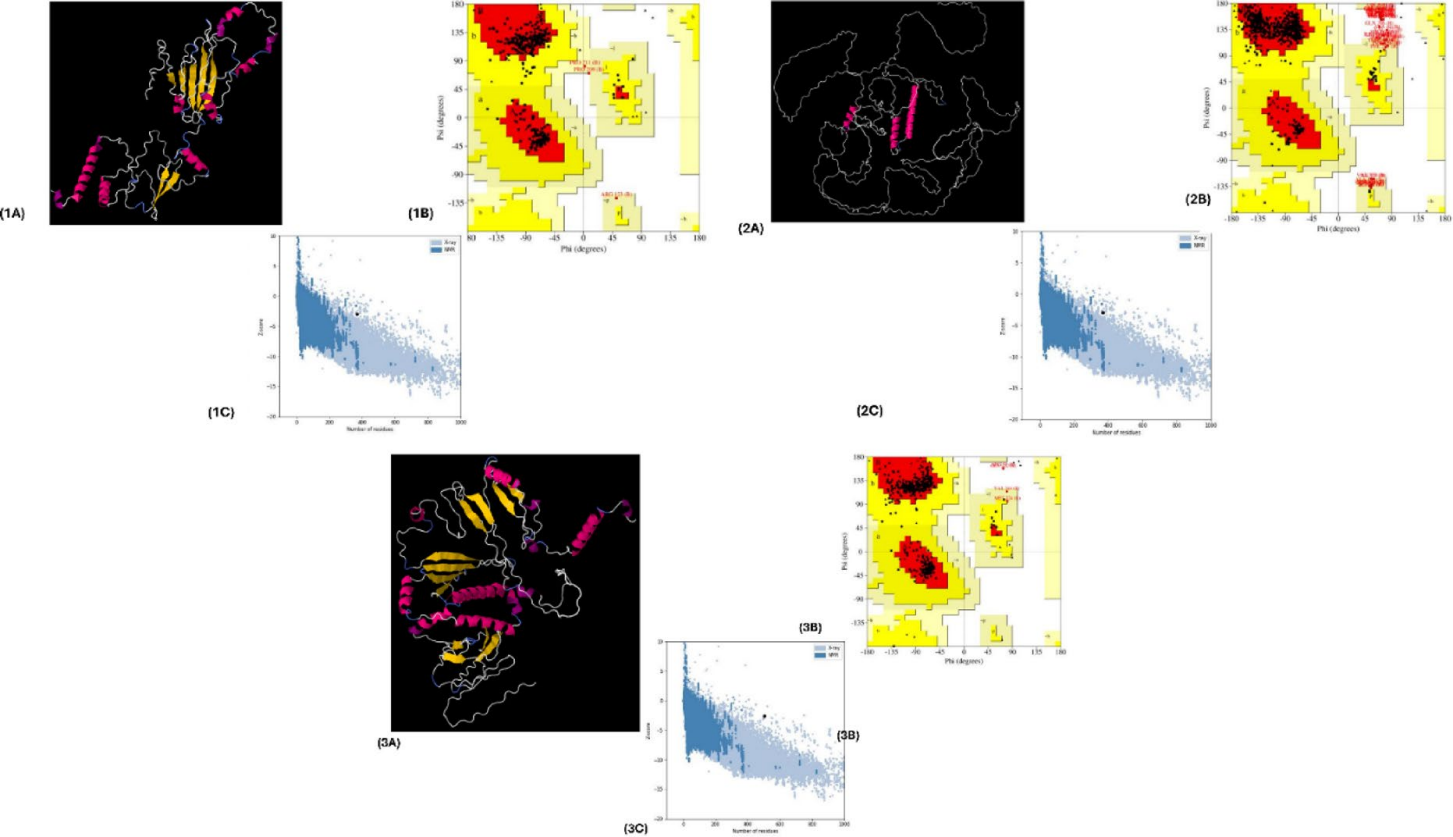
Constructed vaccine candidate against FMDV. (1) version 1, (2) version 2, and (3) version. (**A**) The 3D cartoon representation of the designed vaccines. (**B**) The Ramachandran plot illustrates stereochemical quality. (**C**) The Z-score indicating overall structural quality and stability.

**Table 13:** Evaluation of 3-D structure predicted for three artificial vaccines through different servers according to CASP15.

		TM score	RMSD	Z-Score	QMEANDisco	Overall quality	MolProbity score	Clash score	Ramachandran favored
Version 1	Alphafold3 colab	0.8451	0.693	−2.97	0.24 ± 0.05	76.1928	1.334	2.9	96.2%
	I-TASSER	0.9307	0.484	−4.48	0.27 ± 0.05	71.9101	2.447	13.24	86.6%
	Swiss-model	0.8821	0.607	−1.93	0.34 ± 0.07	65.3846	1.568	8.5	97.5%
Version 2	Alphafold3 colab	0.6674	1.212	−2.97	0.24 ± 0.05	98.6428	1.397	1.5	91.3%
	I-TASSER	0.9263	0.486	−3.8	0.24 ± 0.05	75.2182	2.302	15.4	87.5%
	Swiss-model	0.8958	0.595	0.51	0.34 ± 0.09	27.7778	1.583	6.2	96.3%
Version 3	Alphafold3 colab	0.8388	0.717	−2.54	0.21 ± 0.05	71.5278	0.915	0.8	97%
	I-TASSER	0.8963	0.560	−3.69	0.24 ± 0.05	43.8479	2.868	29.7	82.5%
	Swiss-model	0.9049	0.534	−0.25	0.25 ± 0.06	36.6906	2.026	10.1	91.5%

### Disulfide engineering

The DbD2 analysis identified multiple residue pairs capable of forming stabilizing disulfide bonds. For the final prioritized construct (Vaccine Version 3), two optimal pairs were selected for cysteine mutation based on their favorable geometric parameters (Chi3 angle) and structural location within flexible loops. The selected mutations were Tyr96-Leu99 (chi_3: −94.95ᴼ, Energy: 4.20 kcal/mol) and Pro283-Asp287 (chi_3: −80.17ᴼ, Energy: 5.01 kcal/mol). These engineered disulfide bridges are predicted to significantly reduce the conformational entropy of the unfolded state, thereby enhancing the vaccine’s resistance to thermal degradation during storage and transport. Similar stabilizing pairs Leu28-Arg31 were identified for Versions 1 and 2 (chi_3: −115.56ᴼ, Energy: 3.72 kcal/mol) (Tables S11–S13).

###  Molecular docking

The three versions of the artificial chimeric vaccines were docked with bovine TLR9 and TLR4 using ClusPro 2.0, HADDOCK, and HDOCK servers. Among these platforms, ClusPro 2.0 produced the most reliable docking results, generating 30 complex models based on PPI simulations. ClusPro 2.0 utilizes FFT correlation algorithms to explore billions of possible conformations, providing a highly exhaustive and computationally efficient docking approach. These FFT-based rigid-body methods score and rank predicted complexes through correlation functions that evaluate shape complementarity, electrostatic interactions, and desolvation energy. Although rigid docking techniques typically yield more accurate models within the top-ranked predictions, flexible docking methods such as HADDOCK and HDOCK can provide refined, target-specific conformations with enhanced precision in local interaction modeling^109^.

**Fig. 9:**
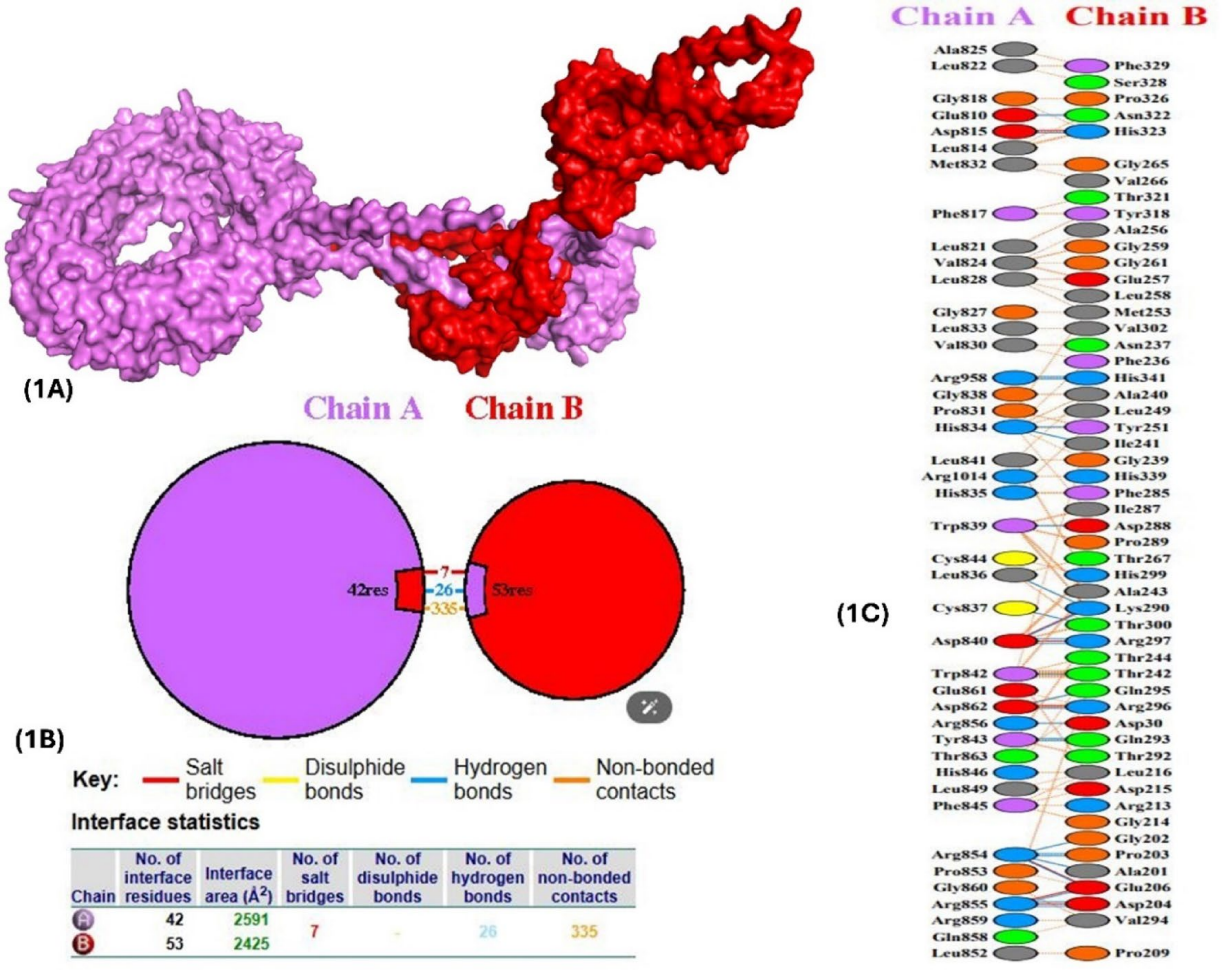
(**A**) Visualization of the docking interaction between the constructed vaccine version 1 and the TLR9 complex, where the vaccine is represented in red and TLR9 in violet. (**B**) Statistical assessment of interaction strength and interface properties. (**C**) Protein–protein interaction (PPI) map illustrating interacting residues and bonding patterns. The same analyses were performed for vaccine version 2.

**Fig. 10:**
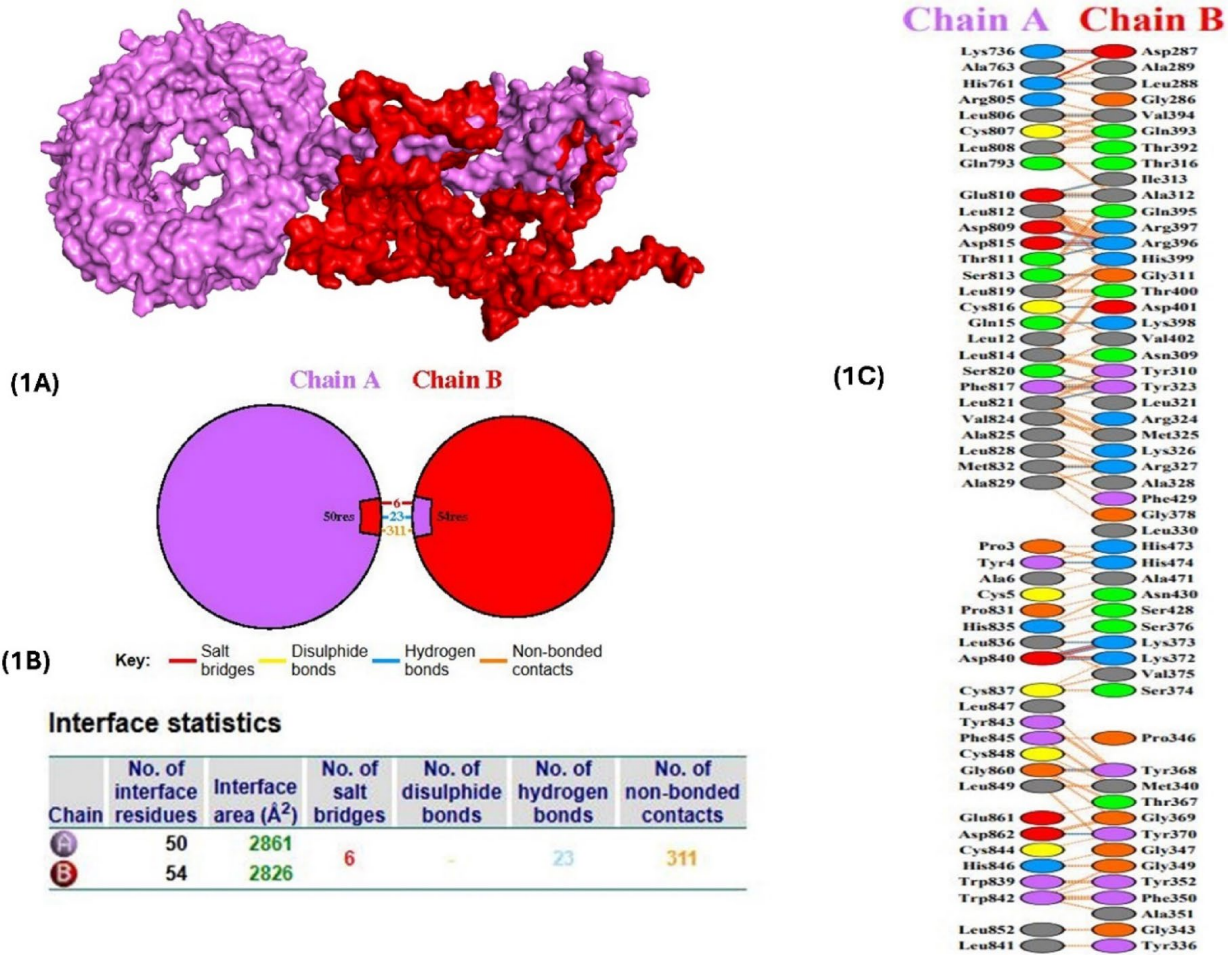
(**A**) Visualization of the docking interaction between the constructed vaccine version 3 and the TLR9 complex, where the vaccine is represented in red and TLR9 in violet. (**B**) Statistical assessment of interaction strength and interface properties. (**C**) Protein–protein interaction (PPI) map illustrating interacting residues and bonding patterns.

**Fig. 11:**
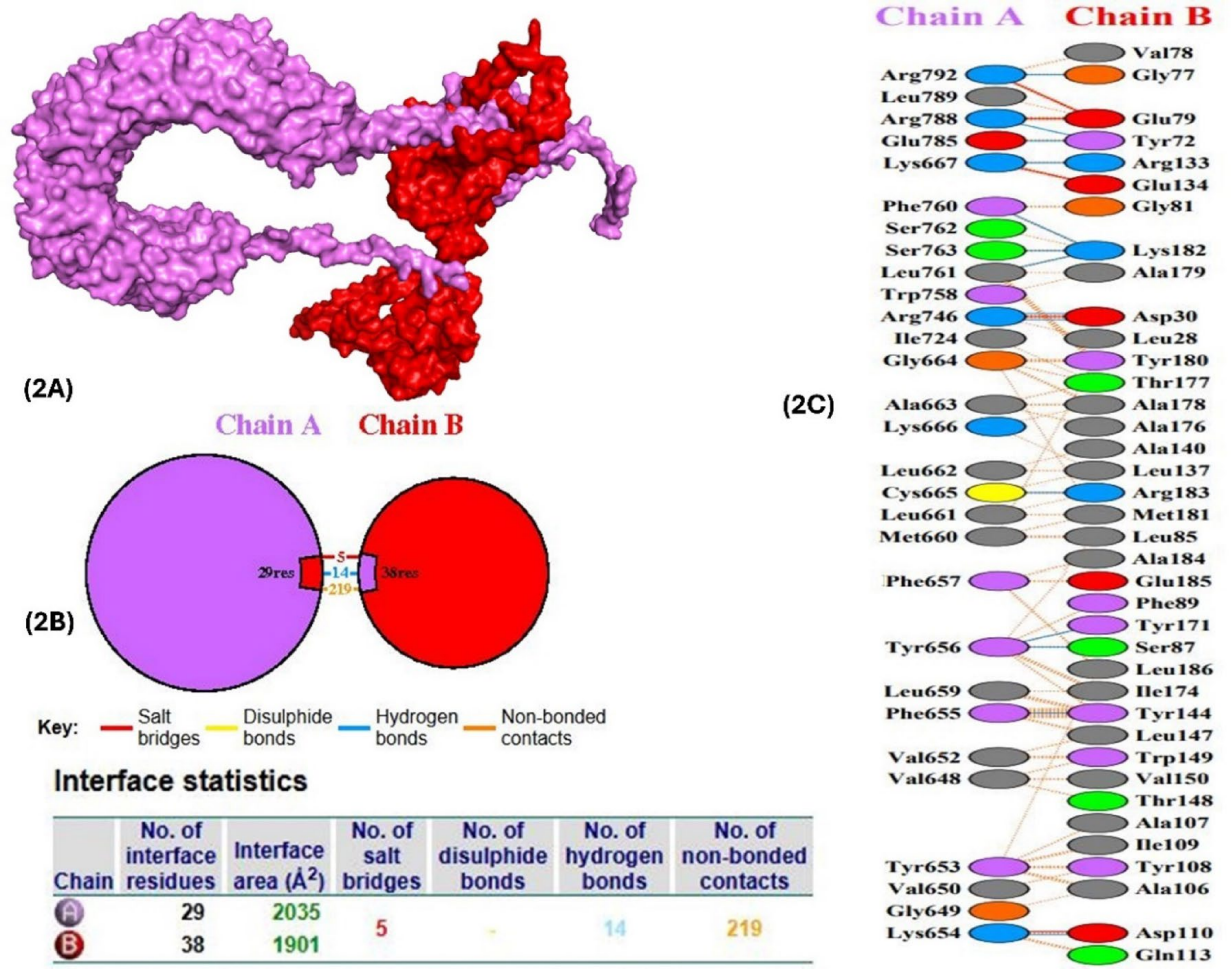
(**A**) Visualization of the docking interaction between the constructed vaccine version 1 and the TLR4 complex, where the vaccine is represented in red and TLR4 in violet. (**B**) Statistical assessment of interaction strength and interface properties. (**C**) Protein–protein interaction (PPI) map illustrating interacting residues and bonding patterns. The same analyses were performed for vaccine version 2.

**Fig. 12:**
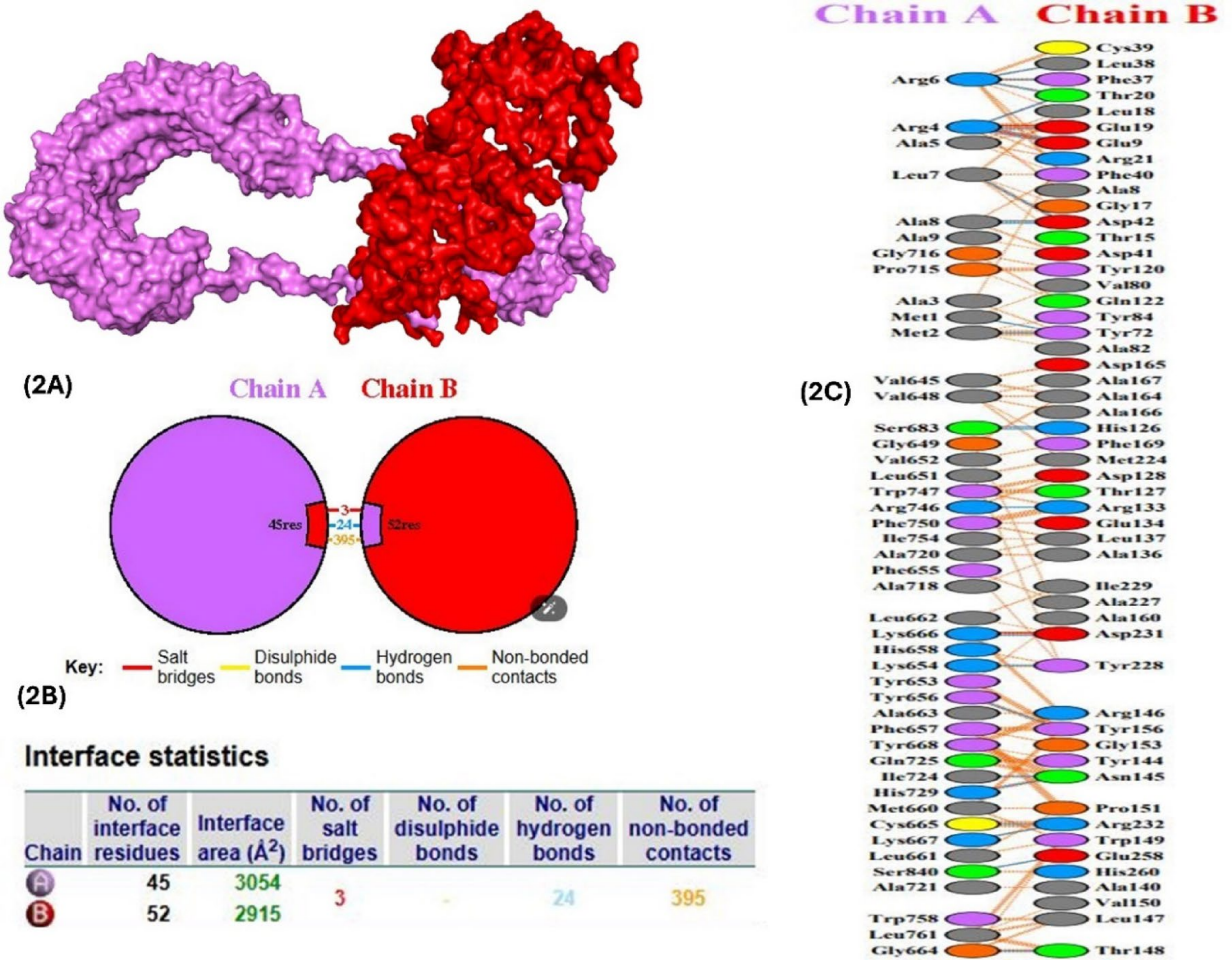
(**A**) Visualization of the docking interaction between the constructed vaccine version 3 and the TLR4 complex, where the vaccine is represented in red and TLR4 in violet. (**B**) Statistical assessment of interaction strength and interface properties. (**C**) Protein–protein interaction (PPI) map illustrating interacting residues and bonding patterns.

For TLR9, the lowest docking scores were − 2093.2, −2093.2, and − 2512.9 kcal/mol in Clusters 0, 5, and 17 for the three versions, respectively. These clusters contained 54, 14, and 17 members, respectively, representing the conformations with the strongest binding affinities. For TLR4, the docking scores revealed − 1912.8 kcal/mol in Cluster 20, which had 12 members for versions 1 and 2, and − 2231.3 kcal/mol in Cluster 0, which had 43 members for version 3. The PRODIGY server was employed to calculate the binding affinity of the three versions of vaccines. ΔG of TLR9 was − 15.8, −15.8, and − 19.4 kcal/mol, and for TLR4 was − 11.4, −11.4, and − 16.9 kcal/mol for the three versions, respectively. The PPI was displayed using the PDBsum server, and Discovery Studio 2021 software was applied to delve into the docking findings **(**Figs. 9, 10, 11 and 12**)**.

### Molecular dynamics simulation

**Fig. 13:**
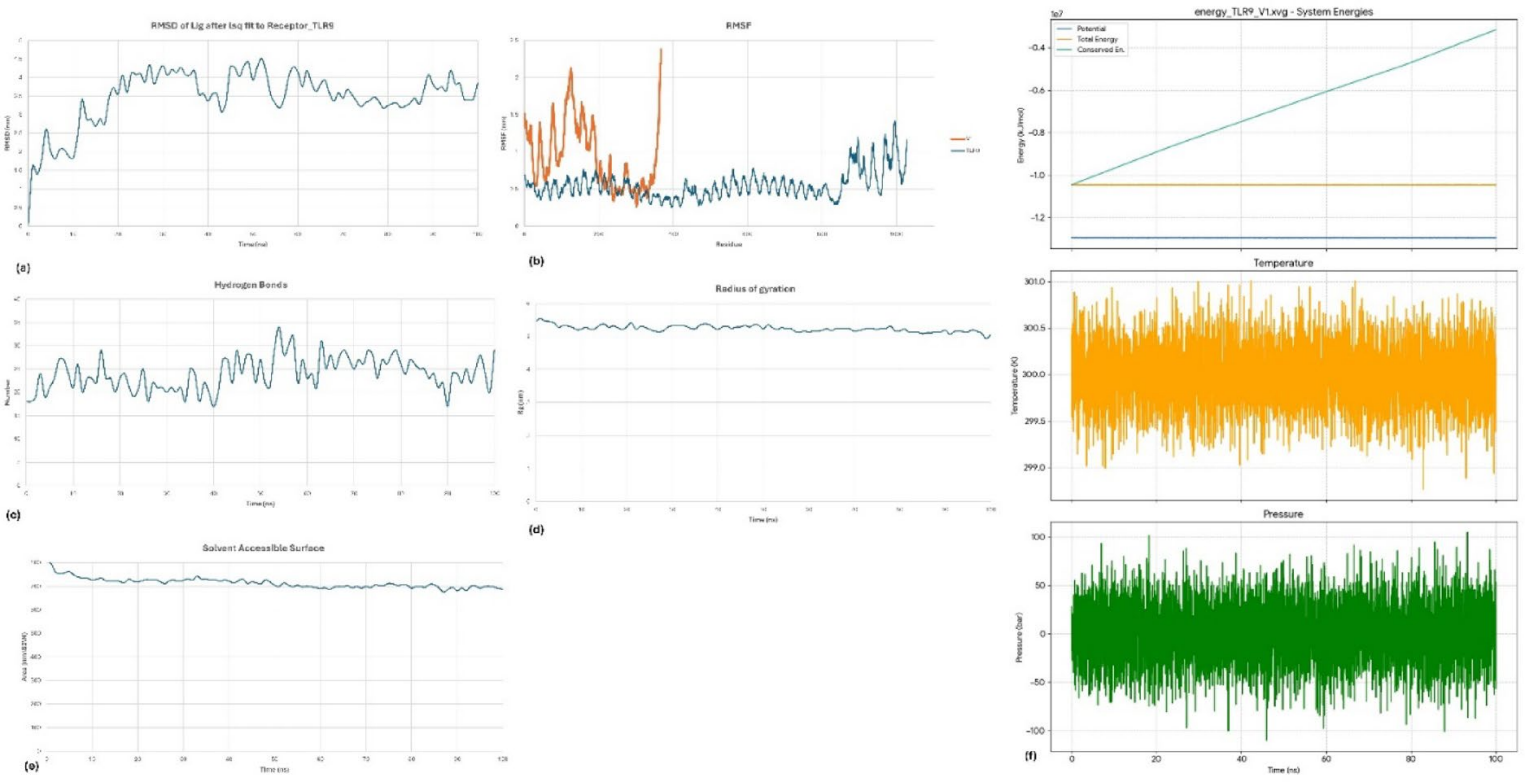
GROMACS molecular dynamics simulations plots of version 1 and the TLR9 complex. (**a**) RMSD, (**b**) RMSF, (**c**) Hydrogen bond, (**d**) Radius of gyration, (**e**) SASA, and (**f**) Energy. The same results for version 2 and the TLR9 complex.

**Fig. 14:**
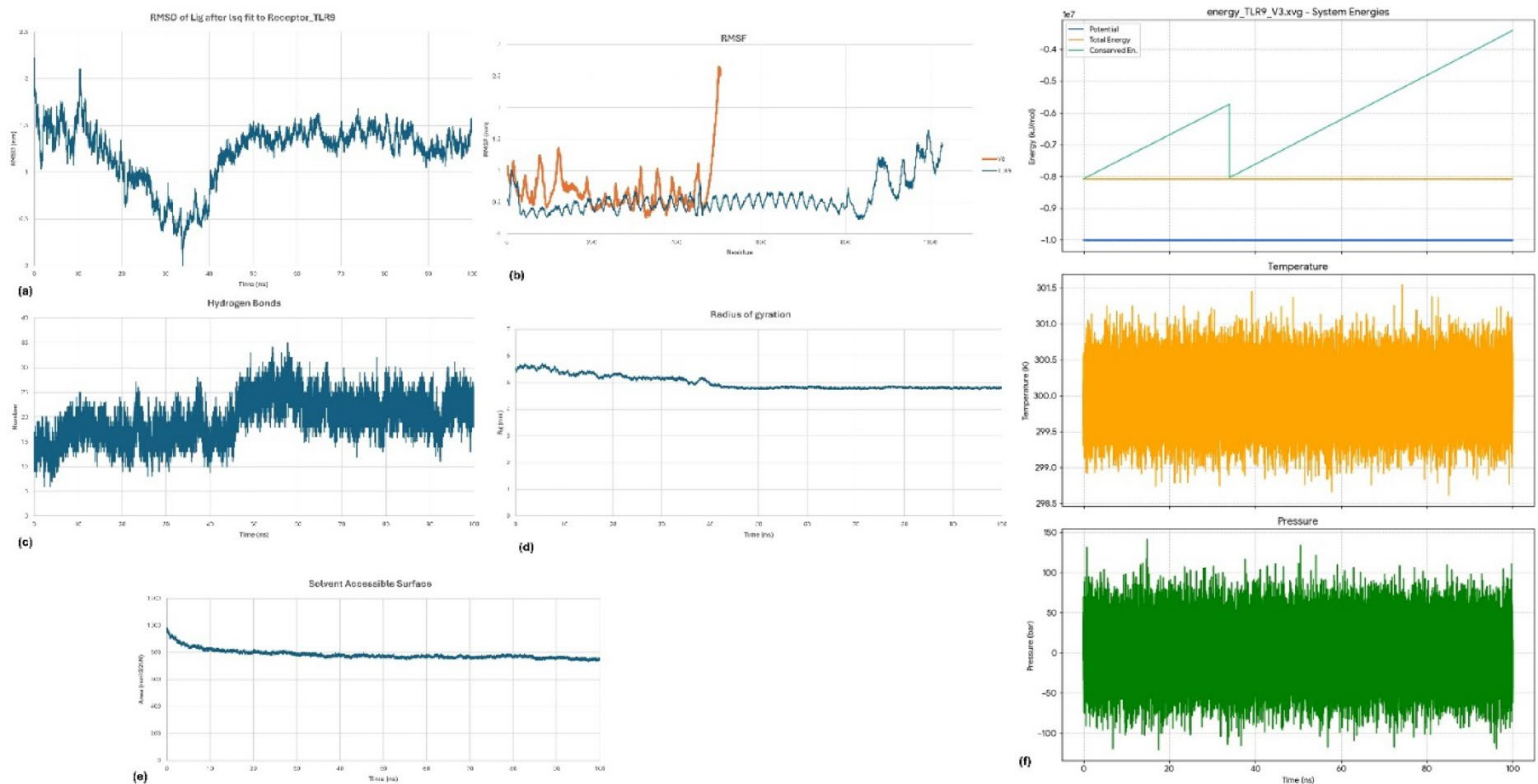
GROMACS molecular dynamics simulations plots of version 3 and the TLR9 complex. (**a**) RMSD, (**b**) RMSF, (**c**) Hydrogen bond, (**d**) Radius of gyration, (**e**) SASA, and (**f**) Energy.

**Fig. 15:**
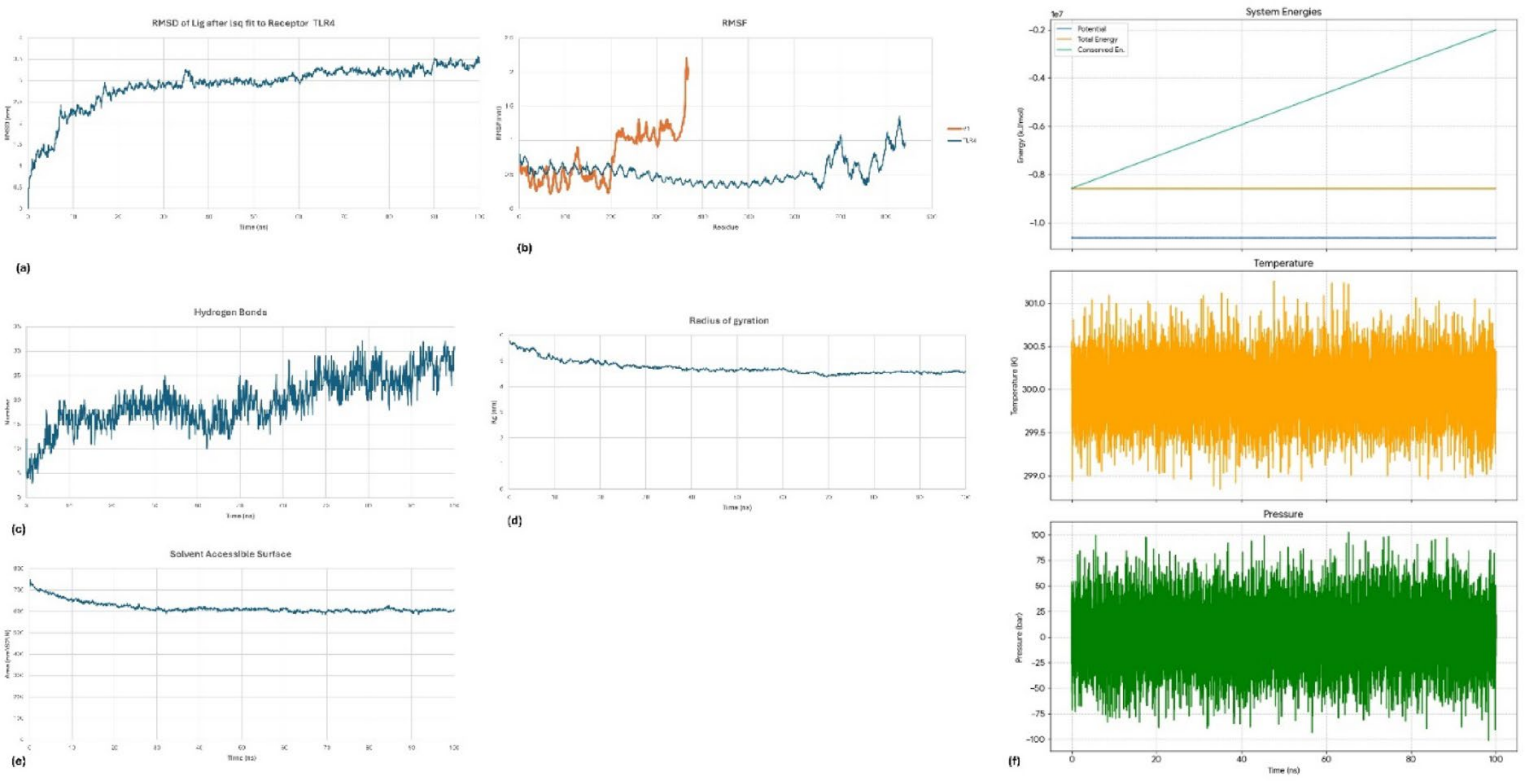
GROMACS molecular dynamics simulations plots of version 1 and the TLR4 complex. (**a**) RMSD, (**b**) RMSF, (**c**) Hydrogen bond, (**d**) Radius of gyration, (**e**) SASA, and (**f**) Energy. The same results for version 2 and the TLR4 complex.

**Fig. 16:**
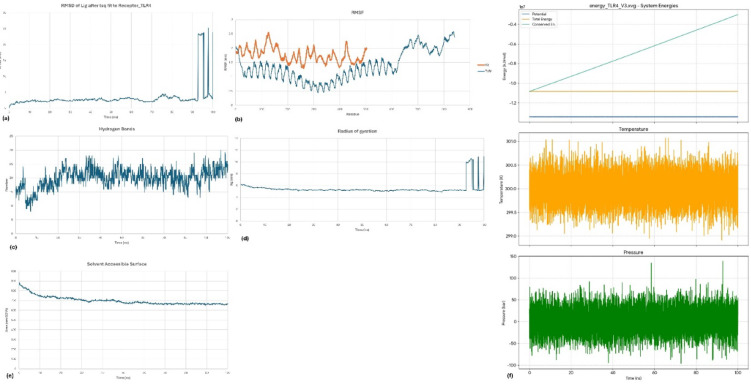
GROMACS molecular dynamics simulations plots of version 3 and the TLR4 complex. (**a**) RMSD, (**b**) RMSF, (**c**) Hydrogen bond, (**d**) Radius of gyration, (**e**) SASA, and (**f**) Energy.

To evaluate the structural stability and dynamic behavior of the vaccine-receptor complexes, the RMSD of the backbone atoms was calculated over the 100 ns simulation trajectory **(**Figs. 13, 14, 15 and 16a**)**. For the TLR9 complexes, both TLR9_V1 and TLR9_V3 exhibited high structural stability. The RMSD profiles showed an initial increase during the equilibration phase but rapidly converged to a stable plateau, indicating that the vaccine candidates maintain a consistent binding pose within the TLR9 receptor site without undergoing significant conformational drift. Similarly, the TLR4 complexes (TLR4_V1 and TLR4_V3) demonstrated robust stability. The TLR4_V1 complex stabilized at an average RMSD of approximately 2.88 nm, while TLR4_V3 showed comparable behavior with minimal fluctuations. The convergence of these RMSD curves confirms that all four systems reached a thermodynamic equilibrium, with the ligand securely anchored in the receptor’s active site.

The local flexibility of the protein residues was analyzed using RMSF to distinguish between rigid core regions and flexible loops **(**Figs. 13, 14, 15 and 16b**)**. In the TLR9 systems (V1 and V3), the overall RMSF values were low, reflecting a stable protein core. Crucially, the residues located at the binding interface exhibited significantly reduced fluctuations (< 0.4 nm), confirming that the binding of the vaccine candidates restricts the mobility of these residues and stabilizes the complex. A similar trend was observed for the TLR4 complexes (V1 and V3), where the RMSF profile indicated that the main structural fluctuations were confined to the N- and C-terminal ends and exposed loop regions, while the crucial Leucine-Rich Repeat (LRR) domains involved in ligand recognition remained rigid and stable.

Intermolecular hydrogen bonds are pivotal for the specificity and affinity of the vaccine-receptor interaction **(**Figs. 13, 14, 15 and 16c**)**. The time-evolution analysis of hydrogen bonds for TLR9_V1 and TLR9_V3 revealed a dense and persistent network of interactions. Both complexes maintained a significant number of hydrogen bonds throughout the simulation, providing the necessary directional anchoring to stabilize the docking pose. For the TLR4_V1 and TLR4_V3 complexes, the analysis similarly displayed a robust occupancy of hydrogen bonds. This continuous formation and reforming of bonds ensures that the vaccine candidates remain tightly bound to the TLR4 receptor surface, preventing dissociation and complementing the electrostatic interactions identified in the binding energy analysis.

The global compactness of the tertiary structure was monitored via the Rg **(**Figs. 13, 14, 15 and 16d**)**. For both TLR9_V1 and TLR9_V3, the Rg values remained relatively constant throughout the 100 ns trajectory, showing no significant drift or abrupt shifts. This indicates that the TLR9 receptor maintains its structural integrity and compactness upon ligand binding. Likewise, the TLR4_V1 and TLR4_V3 complexes exhibited stable Rg profiles, suggesting that the binding of the vaccine candidates does not induce unfolding or significant domain separation in the TLR4 receptor. The consistent Rg values across all four systems support the formation of tight, stable binary complexes.

SASA was calculated to assess the burial of hydrophobic residues and the interaction interface **(**Figs. 13, 14, 15 and 16e**)**. In the TLR9 systems (V1 and V3), the total SASA remained stable, while the interface area analysis indicated a substantial reduction in solvent accessibility upon binding. This points to a favorable hydrophobic effect driving the interaction. Similarly, for TLR4_V1 and TLR4_V3, the stable SASA values and the burial of surface area at the interface confirm that the vaccine candidates fit well into the hydrophobic pockets of the TLR4 receptor, shielding these regions from the solvent and further stabilizing the complex.

Finally, the thermodynamic stability of the systems was rigorously assessed through energy profiles **(**Figs. 13, 14, 15 and 16f**)**. The TLR9_V1 complex showed a robust stability with an average potential energy of approximately − 1.29 × 10⁷ kJ/mol, while TLR9_V3 maintained a stable average of −1.00 × 10⁷ kJ/mol. For the TLR4 systems, TLR4_V1 stabilized at an average potential energy of −1.06 × 10⁷ kJ/mol, and TLR4_V3 exhibited a highly favorable energetic state with an average of −1.34 × 10⁷ kJ/mol. The temperature and pressure for all systems fluctuated stably around 300 K and 1 bar, respectively. These consistent and negative potential energy values confirm that all four simulated complexes are energetically favorable and free from steric clashes, representing stable local minima on the potential energy surface.

### MM-PBSA binding free energy

The binding free energies ($\:{\Delta\:}{\mathrm{G}}_{\mathrm{bind}}$) for the four vaccine-receptor complexes (TLR9-V1, TLR9-V3, TLR4-V1, TLR4-V3) were calculated using the MM-PBSA method. This analysis decomposes the total binding energy into specific contributions: Van der Waals $\:\left({\Delta\:}{\mathrm{G}}_{\mathrm{vdw}}\right)$, Electrostatic ($\:{\Delta\:}{\mathrm{G}}_{\mathrm{ele}}$), Polar Solvation ($\:{\Delta\:}{\mathrm{G}}_{\mathrm{pol}}$), and Non-Polar Solvation ($\:{\Delta\:}{\mathrm{G}}_{\mathrm{np}}$) energies.

The final binding energy ($\:{\Delta\:}{\mathrm{G}}_{\mathrm{Total}}$) is calculated as:

$$\:{\Delta\:}{\mathrm{G}}_{\mathrm{Total}}$$ = $$\:{\Delta\:}{\mathrm{G}}_{\mathrm{Complex}}$$ - $$\:{\Delta\:}{\mathrm{G}}_{\mathrm{R}\mathrm{e}\mathrm{c}\mathrm{e}\mathrm{p}\mathrm{t}\mathrm{o}\mathrm{r}}$$+ $$\:{\Delta\:}{\mathrm{G}}_{\mathrm{Ligand}}$$.

**Fig. 17:**
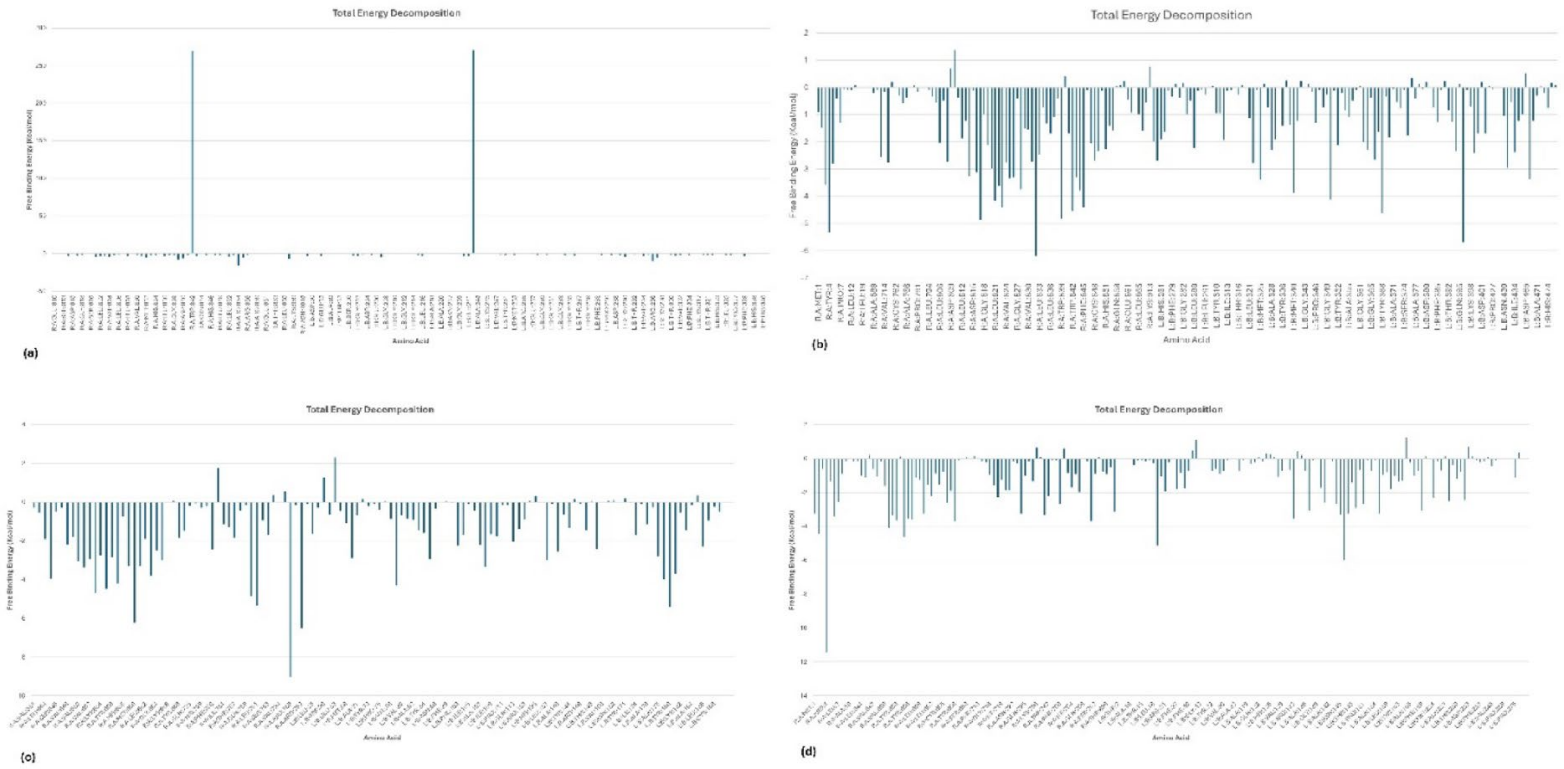
Illustrating the MMPBSA binding affinity for the TLR9_V1 (**a**), TLR9_V3 (**b**), TLR4_V1 (**c**), and TLR4_V3 (**d**).

TLR9_V3 complex exhibits an exceptionally high binding affinity with a $\:{\Delta\:}{\mathrm{G}}_{\mathrm{bind}}\:$of −308.38 kcal/mol. The stability is driven by strong favorable contributions from both Van der Waals forces (−465.95 kcal/mol) and Electrostatic interactions (−550.19 kcal/mol). This indicates a highly complementary fit between the vaccine candidate V3 and the TLR9 receptor, involving both shape complementarity (hydrophobic packing) and specific salt-bridge/hydrogen bond networks **(**Fig. 17a).

In contrast, the TLR9_V1 complex shows a positive total binding energy (+ 289.91 kcal/mol), suggesting thermodynamic instability in this specific simulation setup. While the electrostatic contribution is favorable (−435.78 kcal/mol), the Van der Waals term is positive (+ 254.13 kcal/mol), which typically indicates steric clashes or poor packing at the interface. This suggests that V3 is the superior candidate for TLR9 binding compared to V1 **(**Fig. 17b).

TLR4_V1 complex forms a stable interaction with a$\:\:{\Delta\:}{\mathrm{G}}_{\mathrm{bind}}$ of −247.12 kcal/mol. The binding is dominated by a massive electrostatic contribution (−1080.84 kcal/mol), suggesting that charge-charge interactions (salt bridges) are the primary mechanism of stabilization (Fig. 17c).

Similarly, TLR4_V3 forms a stable complex with TLR4 a$\:\:{\Delta\:}{\mathrm{G}}_{\mathrm{bind}}$ = −205.82 kcal/mol). Like V1, it relies heavily on electrostatic interactions (−868.52 kcal/mol) but has a slightly lower overall affinity compared to V1 **(**Fig. 17d). Both V1 and V3 are strong binders for TLR4, but V1 exhibits a slightly higher calculated affinity (−247 vs. −206 kcal/mol), primarily due to stronger electrostatic engagement with the receptor Table 14.

**Table 14:** Summarizes the calculated $\:{\Delta\:}{\mathrm{G}}_{\mathrm{Components}}$ for each complex (all values in kcal/mol):

Component	TLR9_V1	TLR9_V3	TLR4_V1	TLR4_V3
Van der waals$\:\left({\Delta\:}{\mathrm{G}}_{\mathrm{v}\mathrm{d}\mathrm{w}}\right)$	+ 254.13	−465.95	−359.16	−266.79
Electrostatic ($\:{\Delta\:}{\mathrm{G}}_{\mathrm{ele}}$)	−435.78	−550.19	−1080.84	−868.52
Polar Solvation ($\:{\Delta\:}{\mathrm{G}}_{\mathrm{pol}}$)	+ 516.69	+ 774.47	+ 1242.86	+ 968.14
Non-polar solvation ($\:{\Delta\:}{\mathrm{G}}_{\mathrm{np}}$)	−45.13	−66.72	−49.97	−38.65
Gas phase energy ($\:{\Delta\:}{\mathrm{G}}_{\mathrm{gas}}$)	−181.65	−1016.14	−1440.01	−1135.31
Solvation energy ($\:{\Delta\:}{\mathrm{G}}_{\mathrm{solv}}$)	+ 471.56	+ 707.76	+ 1192.88	+ 929.49
Total binding energy ($\:{\Delta\:}{\mathrm{G}}_{bind}$)	+ 289.91	−308.38	−247.12	−205.82

### Immune simulation

**Fig. 18:**
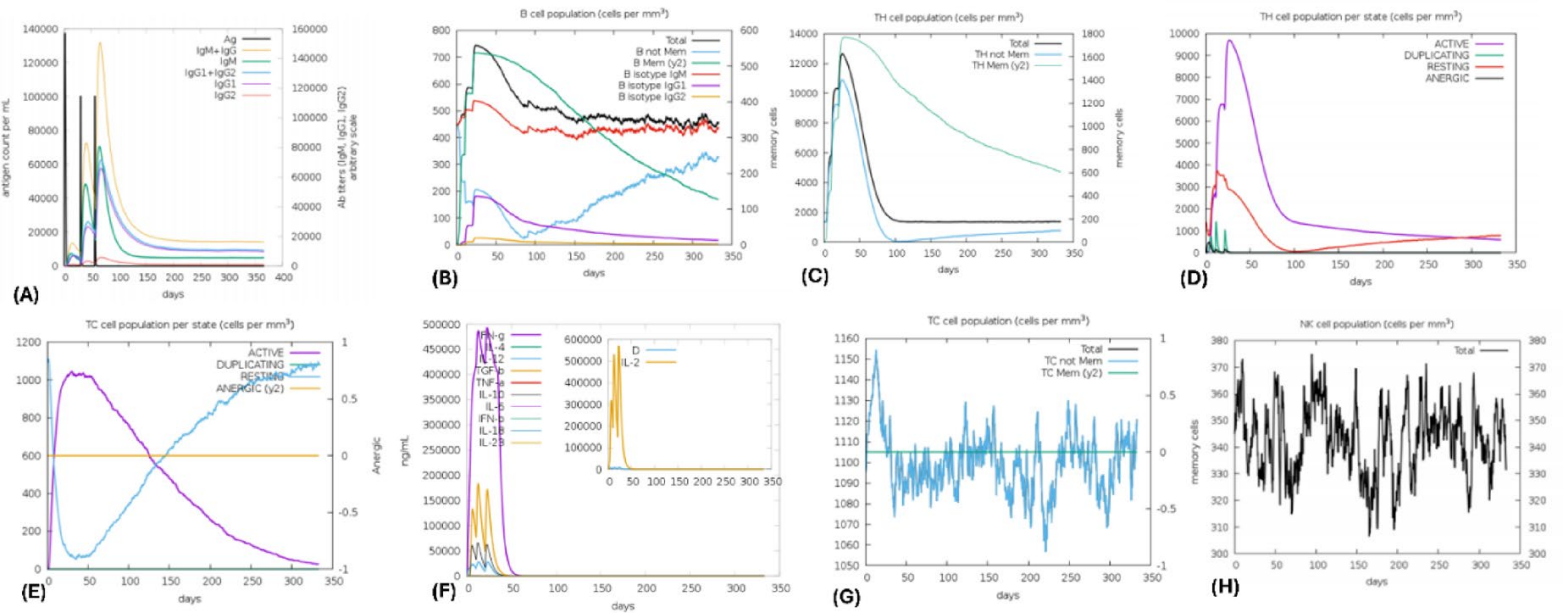
Immune simulation profile of the universal FMD vaccine (version 1). (**A**) Immunoglobulin levels following antigen exposure. (**B**) Distribution of B-cell subtypes. (**C**) Expansion of T-helper lymphocyte (THL) populations. (**D**) THL subsets categorized by activation status. (**E**) Cytotoxic T lymphocyte (CTL) populations based on activation. (**F**) Cytokine responses, including IL-2 and IFN-γ production. (**G**) CTL presents the total memory cells (**H**) Natural killer cells.

**Fig. 19:**
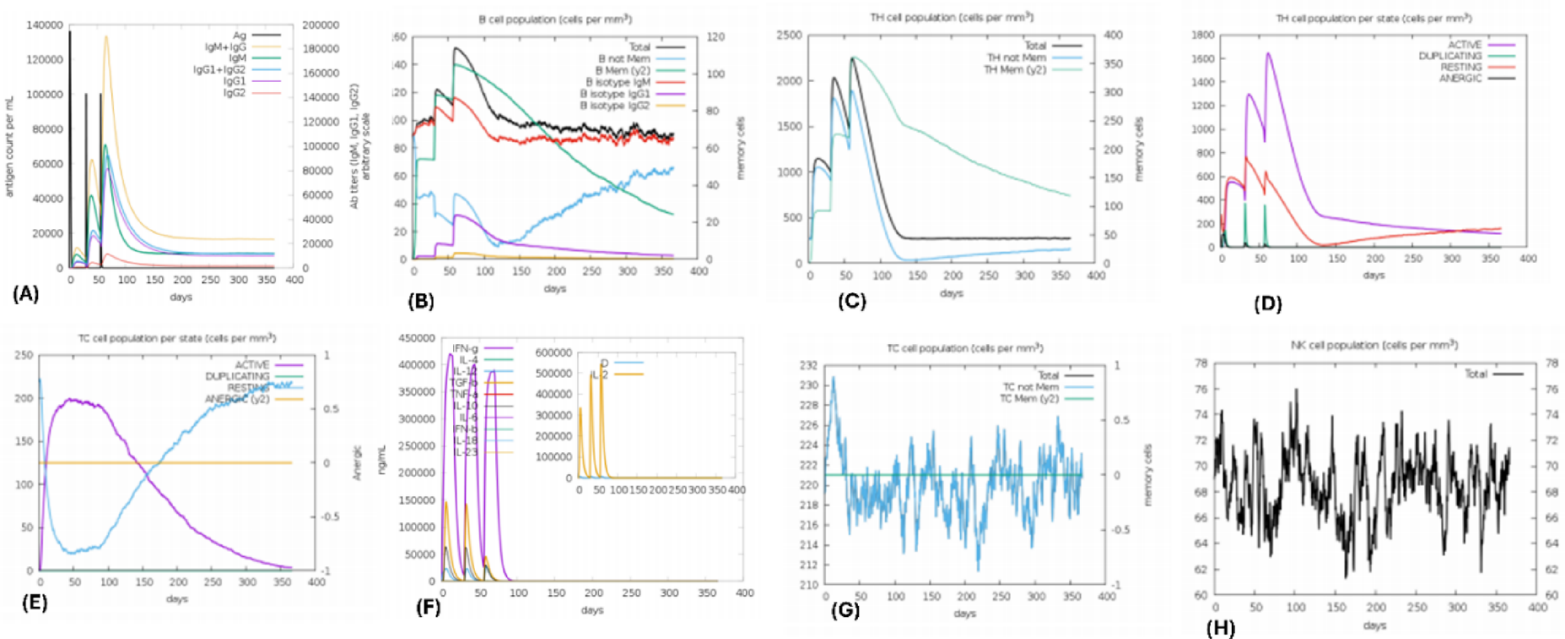
Immune simulation profile of the universal FMD vaccine (version 2). (**A**) Immunoglobulin levels following antigen exposure. (**B**) Distribution of B-cell subtypes. (**C**) Expansion of T-helper lymphocyte (THL) populations. (**D**) THL subsets categorized by activation status. (**E**) Cytotoxic T lymphocyte (CTL) populations based on activation. (**F**) Cytokine responses, including IL-2 and IFN-γ production. (**G**) CTL presents the total memory cells (**H**) Natural killer cells.

**Fig. 20:**
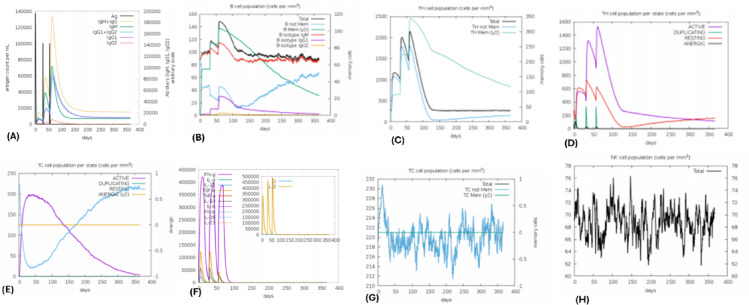
Immune simulation profile of the universal FMD vaccine (version 3). (**A**) Immunoglobulin levels following antigen exposure. (**B**) Distribution of B-cell subtypes. (**C**) Expansion of T-helper lymphocyte (THL) populations. (**D**) THL subsets categorized by activation status. (**E**) Cytotoxic T lymphocyte (CTL) populations based on activation. (**F**) Cytokine responses, including IL-2 and IFN-γ production. (**G**) CTL presents the total memory cells (**H**) Natural killer cells.

The C-ImmSim server was employed to simulate the murine IR elicited by the three designed FMDV chimeric vaccine constructs **(**Figs. 18, 19 and 20**)**. The simulations showed a robust and coordinated activation of cellular and humoral immune responses. (1) Antibody Response: Levels of IgM and IgG antibodies increased progressively following vaccine administration, indicating effective stimulation of primary and secondary immune responses **(**Figs. 18A, 19A, 20A**)**. (2) B-Cell Activation: B-cell populations showed a marked increase in memory B-cells, confirming long-term humoral protection **(**Figs. 18B , 19B, 20B**)**. (3) Helper T-Cell (THL) Response: The population of helper T lymphocytes (THLs) expanded significantly and peaked following vaccination, demonstrating enhanced immune activation **(**Figs. 18C, D, 19C,D, 20C,D**)**. (4) CTL Response: CTL populations increased progressively after vaccination, indicating the induction of cellular immune memory **(**Figs. 18E, 19E, 20E**)**. (5) Cytokine Production: Elevated levels of interleukin-2 (IL-2) and interferon-gamma (IFN-γ) were observed, reflecting strong antigen-specific immune stimulation **(**Figs. 18F, 19F, 20F**)**. (6) Cytotoxic T-cell Dynamics: The dynamics of the CTL population were analyzed, revealing active engagement and proliferation in response to antigen injections (Figs. 18G, 19G, 20G). (7) Natural Killer (NK): The vaccine triggered immediate innate activity, evidenced by the rapid fluctuation of NK cells (Figs. 18H, 19H, 20H).

Overall, these findings highlight that the three FMDV chimeric vaccines can trigger robust cellular and humoral IR in mice, characterized by high antibody titers and increased cytokine production, underscoring their potential as universal FMDV vaccine candidates.

### In silico cloning and expression feasibility

The reverse translation and codon optimization of the three vaccine constructs resulted in nucleotide sequences with ideal physicochemical properties for expression in *E. coli.* The JCat analysis revealed a CAI of 1.0 for all three versions, indicating perfect codon usage alignment with the host’s translation machinery. Furthermore, the GC content was optimized to 54.53% (Version 1), 52.98% (Version 2), and 53.97% (Version 3), falling well within the optimal range (40–60%) to ensure transcript stability.

**Fig. 21:**
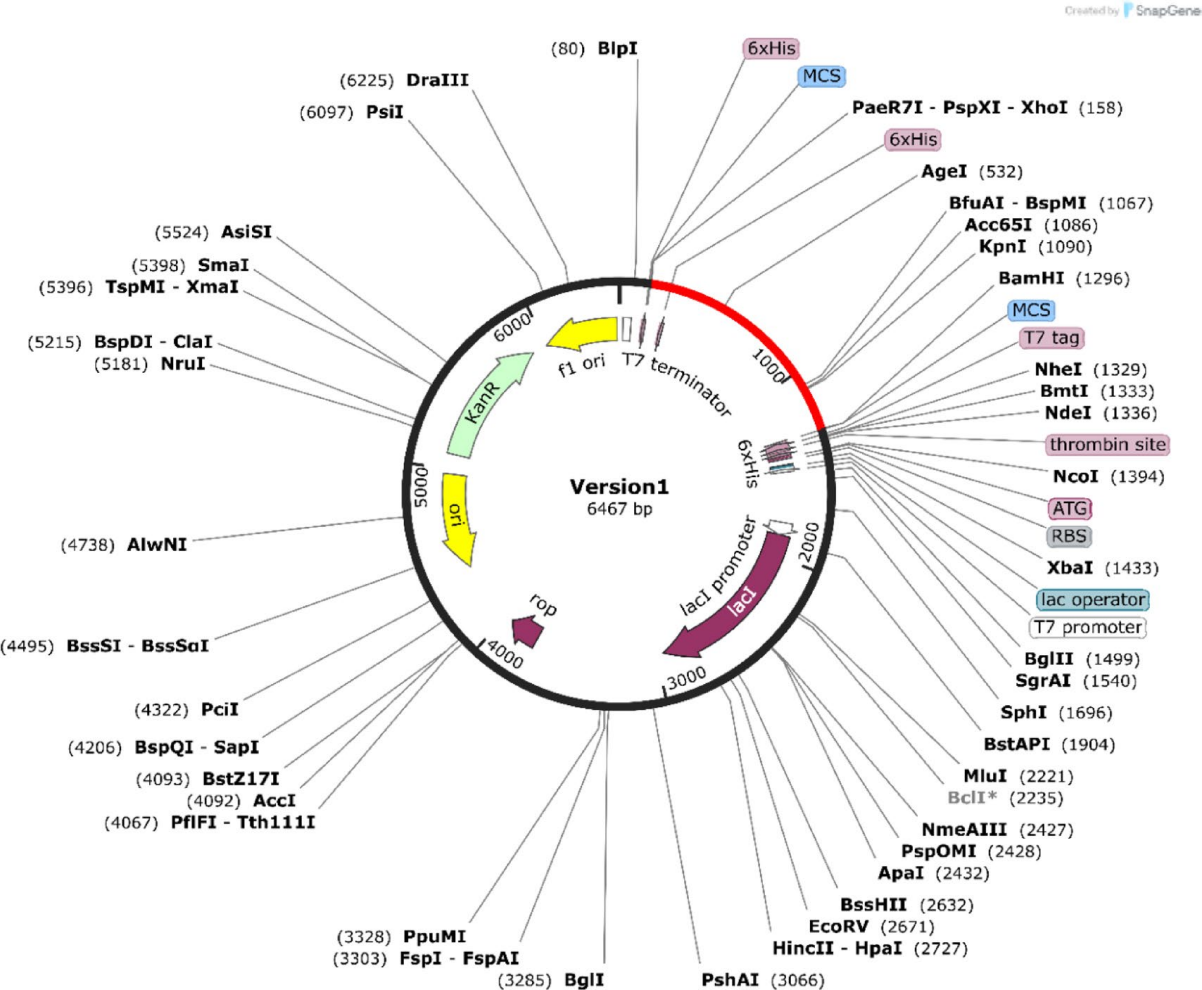
In silico restriction cloning of Vaccine Version 1 into the pET-28a(+) expression vector. The circular plasmid map, generated using SnapGene, displays the optimized vaccine gene insert (red) cloned between the BamHI (5’) and XhoI (3’) restriction sites. The construct is designed for high-level expression under the control of the T7 promoter.

**Fig. 22:**
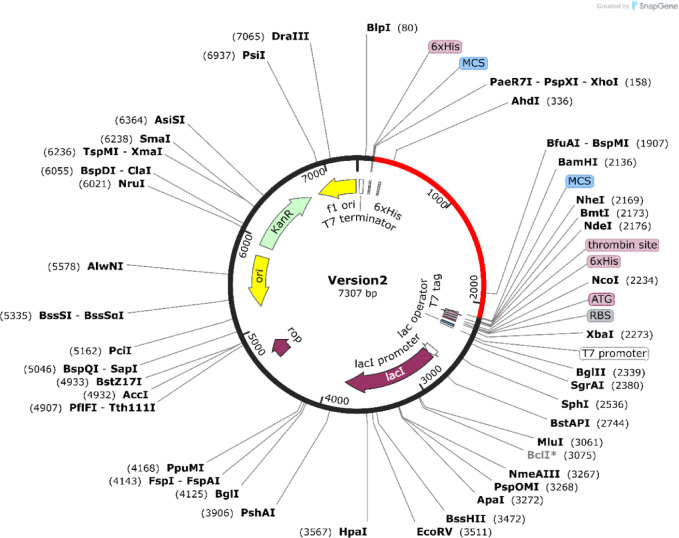
In silico restriction cloning of Vaccine Version 2 into the pET-28a(+) expression vector. The circular plasmid map, generated using SnapGene, displays the optimized vaccine gene insert (red) cloned between the BamHI (5’) and XhoI (3’) restriction sites. The construct is designed for high-level expression under the control of the T7 promoter.

**Fig. 23:**
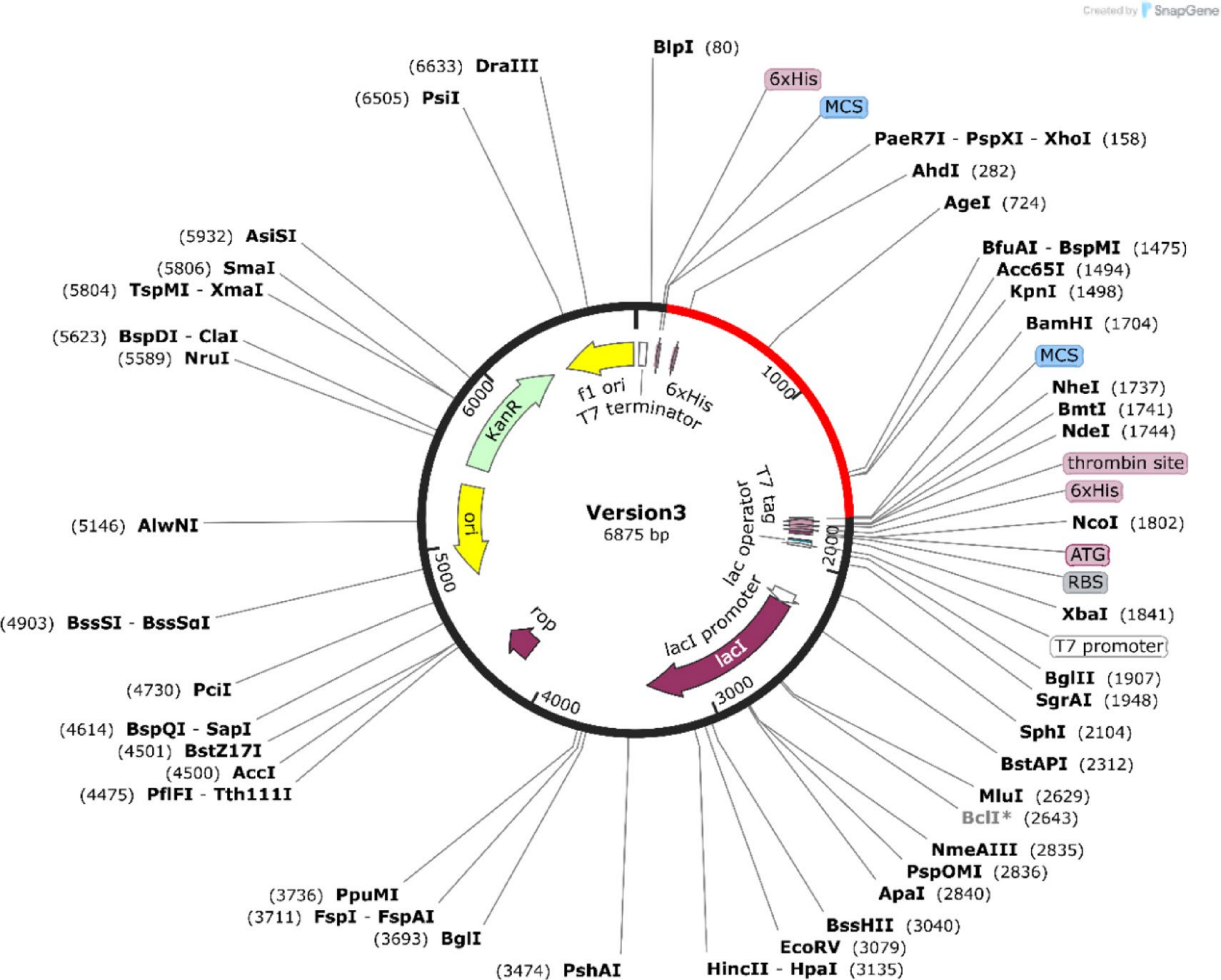
In silico restriction cloning of Vaccine Version 3 into the pET-28a(+) expression vector. The circular plasmid map, generated using SnapGene, displays the optimized vaccine gene insert (red) cloned between the BamHI (5’) and XhoI (3’) restriction sites. The construction is designed for high-level expression under the control of the T7 promoter.

The in silico cloning simulation in SnapGene confirmed the successful insertion of the vaccine genes into the pET-28a(+) vector. The addition of BamHI and XhoI adapters facilitated precise directional insertion. The resulting recombinant plasmids, pET28a-VacV1 (6,473 bp), pET28a-VacV2 (7,313 bp), and pET28a-VacV3 (6,881 bp), are depicted in **(**Figs. 21, 22 and 23**)**, respectively. The maps confirm that the vaccine genes are correctly flanked by the T7 promoter and T7 terminator, maintaining the correct reading frame for the N-terminal 6xHis-tag. These constructs were verified to maintain the correct reading frame for successful high-level expression in the *E. coli* system.

## Discussion

Foot-and-mouth disease virus (FMDV) remains a major challenge for livestock health because of its high variability and rapid mutation rate^110^. These characteristics limit the effectiveness of serotype-specific vaccines and allow outbreaks to occur even in vaccinated populations^111,112^. This situation emphasizes the need for broad-spectrum vaccines capable of providing cross-serotype protection.

In this study, two artificial chimeric proteins (ACP1 and ACP2) were designed by combining conserved regions from structural proteins (VP1–VP3) and non-structural proteins (3 A and 3 C) of Egyptian FMDV serotypes A, O, and SAT2. The goal was to create a broad-spectrum vaccine candidate capable of triggering strong and long-lasting immunity. The structural proteins contain the dominant neutralizing sites, including the conserved RGD motif in VP1, which mediates integrin binding and drives strong antibody responses^10,113–115^. The non-structural proteins add T-cell–stimulating regions that help generate a broader immune response^13,116^.

The domain composition of the chimeric proteins provides insight into their immunological behavior. The Rhv/Rhv-like domain contributes to capsid stability and proper exposure of conformational epitopes needed for neutralizing antibody recognition^107^. Rhv plays a central role in forming the icosahedral shell that protects viral RNA and facilitates receptor binding, a key step for viral entry into host cells, consistent with previous findings. Structurally, the Rhv domain’s eight-stranded beta-sandwich configuration ensures stability while presenting key antigenic sites for immune recognition^105^. The presence of Calici_coat–related features, particularly within the P2 subdomain, suggests an enhanced display of antigenic regions that improve B-cell activation^106^. In addition, the Peptidase_C3 domain, derived from the 3 C protease, supports antigen processing and T-cell activation through its conserved proteolytic and host-interaction functions^117,118^. These preserved domains strengthen the biological relevance of ACP1 and ACP2 and indicate their ability to mimic critical viral features.

Post-translational modification predictions add another layer of biological relevance. Additionally, Phosphorylation sites contribute to the regulation of PPIs and signaling pathways, which are crucial for activating immune responses^119^. Glycosylation, on the other hand, enhances protein stability, solubility, and immune recognition by mimicking natural viral antigens, thereby improving the immunogenicity of the chimeric proteins^120^. Also, Tensins, especially PTEN, have a significant relationship with manipulating FMDV to the host cell signaling pathways to enhance its replication and evade immune detection. PTEN, as a lipid phosphatase, has a vital role in regulating the PI3K/Akt signaling pathway, which FMDV may exploit to facilitate its lifecycle by inhibiting pathways that promote cell survival and growth^121^. The integration of these PTMs into ACP1 and ACP2 ensures they closely resemble natural viral proteins, further increasing their potential to stimulate both cellular and humoral IR. These findings underscore the importance of incorporating PTMs into vaccine design, as they play a pivotal role in optimizing protein functionality and enhancing overall immunogenicity.

The physicochemical properties of ACP1 and ACP2 reveal that the proteins are thermally stable, with high aliphatic indices and low instability scores. Secondary structure prediction displayed a balanced composition of alpha helices, beta sheets, and random coils across the ACP1 and ACP2 proteins, which agrees with a study performed by^122^. This structural diversity ensures proper folding and functionality while enhancing immunogenicity. The absence of signal peptides in transmembrane topology predictions suggests that these proteins are well-suited for extracellular secretion, simplifying vaccine formulation and delivery, and this is the same finding with^123^.

The immunological architecture of ACP2 includes diverse B-cell, CTL, and Th epitopes. This variety increases the likelihood of activating both humoral and cellular responses. Similar epitope-rich constructs have been reported in earlier multi-epitope vaccine studies targeting bacteria and viruses^22,80,81,107,122–128^. Neutralizing epitopes from VP1 support strong antibody generation, and the inclusion of non-structural epitopes enhances T-cell engagement. Furthermore, the inclusion of an adjuvant sequence at the C-terminal of the designed vaccine constructs contributed to an increased estimated half-life, aligning with observations reported in earlier studies^75^. Observed cytokine patterns, such as IL-2 and IFN-γ production, indicate activation of both Th1 and Th2 responses^66,129^.

The consensus-based design produces both conserved and mosaic epitopes. The conserved epitope core aligns with circulating isolates, while the mosaic epitopes expand the immune coverage by capturing variation across A, O, and SAT2. These synthetic epitopes contain the most frequent residues found in multiple strains. This approach increases the likelihood of recognition across diverse BoLA alleles in cattle. This strategy is particularly advantageous in regions with high antigenic variability. Notably, our findings align with those of Raza et al. (2019)^107^, who demonstrated that consensus-derived epitopes enhance broad immune coverage in rapidly evolving viruses. However, these synthetic epitopes require experimental confirmation through assays such as competitive ELISA and virus-neutralization tests.

General reviews of multi-epitope vaccines in livestock articulate the typical pipeline and evaluation metrics we adopted^9^. When compared to recent computational vaccine designs from China and Africa, our construct exhibits two major improvements:

(1) Cross-serotype design using a consensus sequence rather than serotype-specific epitopes^103^;

(2) Higher structural stability based on combined TM-score, RMSD, and stability predictions.

Studies from China often focus on single serotypes, while African studies mainly emphasize VP1 alone^4,130^. Our multi-serotype consensus strategy positions ACP2 as a more universal immunogen, potentially better suited for regions like Egypt where multiple serotypes co-circulate.

Collectively, these findings suggest that ACP2 integrates antigenic breadth with structural robustness, making it a promising candidate for future experimental evaluation.

The MM-PBSA analysis identifies TLR9_V3 and TLR4_V1 as the most energetically favorable complexes for their respective receptors. For TLR9, Candidate V3 is clearly superior, showing high affinity (−308.38 kcal/mol) while V1 appears unstable. For TLR4, both candidates bind strongly, with V1 showing a modest advantage in binding energy (−247.12 kcal/mol) over V3.

These results align with the structural stability observed in the RMSD/RMSF analyses, confirming that V3 (for TLR9) and V1 (for TLR4) are the most promising multi-epitope vaccine candidates.

The immune simulation offers important insight into how the vaccine may behave under real field conditions. Using a standard three-dose schedule, the model showed that the constructs can effectively trigger immunological memory. The strongest response was the humoral one. Both IgG1 and IgG2 levels rose sharply and remained elevated long after the final booster. This is encouraging because protection against FMDV depends mainly on neutralizing antibodies that prevent viral entry.

The simulation also indicated a dominant Th1-type response, reflected by increased IFN-γ levels. This cytokine pattern supports isotype switching and enhances antiviral defenses. Although strong T-helper and B-cell memory populations were generated, CTL memory was less prominent. This suggests that the vaccine’s protective effect is likely driven through antibody-mediated mechanisms supported by CD4 + T-cells rather than direct CD8 + cytotoxic activity. This aligns well with known correlates of FMDV protection, where circulating antibody titers remain the most reliable indicator of immunity. Importantly, the durability of the simulated immune response over the 350-day period suggests that the vaccine could match the semi-annual vaccination schedule commonly used in FMD-endemic regions such as Egypt.

Overall, the biological interpretation of our findings suggests that ACP2 offers a strong combination of structural stability, immunological breadth, and domain functionality. These features support its potential as a universal FMDV vaccine candidate and justify further laboratory and in vivo evaluation.

This study is based entirely on computational predictions and therefore has several inherent limitations. Structural modeling, antigenicity scoring, epitope mapping, and stability assessments depend on algorithmic assumptions that may not fully replicate biological behavior. Additionally, predicted B-cell epitopes may differ in conformation when expressed in vitro, and immunogenicity scores do not guarantee effective immune responses in vivo. The protective efficacy of ACP2 cannot be confirmed without experimental validation, including expression profiling, antigen purification, immunogenicity assays, and multi-serotype challenge studies in relevant animal models. Therefore, the results presented here should be considered preliminary until supported by laboratory and field evaluations.

## Limitations and future perspectives

Despite the comprehensive computational framework applied in this study, several limitations should be acknowledged. The present work is entirely based on in silico analyses, including epitope prediction, structural modeling, molecular docking, molecular dynamics simulations, and immune simulations. While these approaches provide valuable insights into the potential immunogenicity and stability of the proposed vaccine construct, they cannot fully replicate the complexity of biological systems.

Therefore, experimental validation remains essential to confirm the safety, immunogenicity, and protective efficacy of the designed vaccine. Future studies should focus on in vitro expression and purification of the chimeric protein, followed by immunological assays to evaluate antigenicity and cytokine responses. In addition, in vivo studies using appropriate animal models are required to assess the immune response, protection efficiency, and possible adverse effects.

Overall, the current study provides a rational computational foundation that can guide subsequent experimental vaccine development against FMDV.

## Conclusion

This study successfully established a computational framework for a broad-spectrum FMDV vaccine, integrating evolutionary insights from Egyptian serotypes A, O, and SAT 2. The results conclusively demonstrate that the ACP2 construct possessed the strongest antigenicity and structural stability among all the ACPs tested, driven by the synergistic integration of conserved structural domains with immunogenic non-structural proteins. The robust binding to bovine TLRs and the simulation of sustained B- and T-cell memory responses suggest that ACP2 overcomes the serotype-specificity limitations of current inactivated vaccines. Future research directions must now pivot from computational design to experimental validation, including: (1) in vitro expression and purification of the recombinant ACP2 protein to assess solubility and yield; (2) in vivo immunogenicity analysis in murine and bovine models to quantify antibody titers and cytokine profiles; and (3) comprehensive interserotype challenge tests to confirm broad-spectrum protection against circulating field isolates.

## Supplementary Information

Below is the link to the electronic supplementary material.

Supplementary Material 1

Supplementary Material 2

## Data Availability

All data generated or analyzed during this study are included in this published article. All sequence data presented in this study are publicly available in the NCBI GenBank database; accession numbers are included in Supplementary Table S1.
