# Supplementary material for: Immunoinformatics-based design of artificial chimeric proteins as universal vaccine candidates against foot-and-mouth disease virus serotypes A, O, and SAT2
Source: Sci Rep. 2026 Jun 9;16:17866. doi: 10.1038/s41598-026-49135-5 (PMC13249879; doi:10.1038/s41598-026-49135-5)
Supplement: Supplementary file 1 — Supplementary Material 1 [file 41598_2026_49135_MOESM1_ESM.docx]

Table (S1) The total sequences retrieved of A, O, and SAT2 serotypes of FMDV in Egypt

| Serotype A | | | | | | | | | |
| --- | --- | --- | --- | --- | --- | --- | --- | --- | --- |
| MW413345 | KX258044 | OL769314 | KC440882 | OL769315 | MT597128 | MT508912 | MT442149 | KC888938 | KC888939 |
| KR092701 | MT442146 | MH732982 | MT863265 | MG552838 | MG552842 | MT442153 | MG552841 | MG552839 | MT442154 |
| MH732981 | MN853329 | EF208756 | EF208757 | EF208762 | EF208765 | EF208766 | EF208774 | EF208769 | EF208773 |
| KX083565 | KT699210 | MG552840 | MT597129 | MT863267 | MT199285 | MG957500 | KC888937 | MG552837 | MK422570 |
| KX447000 | MT863264 | MT863266 | MT442165 | MT442167 | ON380439 | ON380440 | OL456140 | OP321262 | MN853330 |
| OQ302221 | OQ302225 | MT597127 | MG552843 | EF208770 | OP823161 | MK572799 | MK422573 | MK422574 | KX446998 |
| MW792216 | MW792217 |  | | | | | | | |
| Serotype O | | | | | | | | | |
| MK422548 | EU667451 | AJ004658 | DQ164871 | OM221189 | KJ210073 | AJ251477 | MF322684 | ON569816 | JQ837834 |
| MG571528 | KX258001 | MT443080 | OM221178 | OM221191 | OM221201 | OM221233 | MF322685 | MF962876 | JQ837835 |
| KT121465 | KX258004 | MN296504 | GU566059 | MG552845 | MN296506 | OM221183 | OM221196 | MT597125 | OM221194 |
| EU553840 | MG552844 | MN296503 | MG552846 | MN296507 | OM221204 | OM221205 | OM221232 | MT443078 | OM221186 |
| MG552848 | OM221212 | MF322680 | OM221185 | OM221197 | OM681355 | MT597122 | MT597123 | KT121467 | MT597124 |
| KX447123 | MF322683 | MF322679 | MG552849 | AJ004655 | OK558889 | KJ210078 | KJ210075 | KP940473 | OM681354 |
| Serotype SAT 2 | | | | | | | | | |
| JX570617 | JX570633 | AF540910 | MZ097483 | JX570622 | MT597121 | MT597120 | MZ097480 | MZ097482 | MG552850 |
| JX013980 | KX258067 | MT199283 | MZ097481 | KY372408 | KY372409 | KY372410 | MT450473 | MT597118 | MT597119 |
| JX570620 | KX258066 | MF322695 | MZ146910 | MH732988 | MH732987 | MH732985 | MH732984 | MZ097479 | JX014255 |
| JX570621 | KF112932 | MF322696 | OL448982 | KF112936 | KF112937 | KF055860 | KF055861 | KX258063 | KX258064 |
| KF112935 | KF112934 | JX570623 | JX570624 | JX570625 | JX570626 | MN864516 | JX570627 | MG552851 | MT602089 |
| JX014256 | KC440884 |  | | | | | | | |

Table (S2) B-cell Prediction of ACP1 Protein Target Through Different Servers

| Server | Sequence | Position | Score | Antigenicity | Allergenicity | toxicity |
| --- | --- | --- | --- | --- | --- | --- |
| SVMTrip | LMQTREHALVGALLRAATYY | 412 - 431 | 0.309 | 0.5454 | Non-allergen | Non-toxin |
|  | LTWVPNGAPETALLNTTNPT | 446 - 465 | 0.239 | 0.6343 |  |  |
|  | TINLHFMFTGPTDHKARYMV | 241 - 260 | 0.235 | 0.8035 |  |  |
| ABCpred | SVSAGPDLEFRFPIDP | 342-357 | 0.91 | 1.2242 |  |  |
|  | TFSIPYISAADYAYTA | 296-311 | 0.84 | 0.8461 |  |  |
|  | REEYQLTLFPHQFINP | 111-126 | 0.84 | 0.5548 |  |  |
|  | DTNHTLPPSFNYGAIT | 517-532 | 0.82 | 1.0708 |  |  |
|  | SAADYAYTASDTAETT | 303-318 | 0.80 | 0.7440 |  |  |
|  | LCSLKDREEYQLTLFP | 105-120 | 0.78 | 1.2691 |  |  |
|  | DAAHCIHAEWDTGLNS | 278-293 | 0.73 | 0.6249 |  |  |
|  | VEVSAVGNQFNGGCLL | 83-98 | 0.71 | 0.7906 |  |  |
|  | DLAALAAKYADTNHTL | 507-522 | 0.61 | 0.9630 |  |  |
|  | TQVQRRKHTDVAFILD | 381-396 | 0.61 | 1.1112 |  |  |
|  | YKRHKPWTLVVMVVTP | 147-162 | 0.60 | 0.8935 |  |  |
|  | SFVVDLMQTREHALVG | 407-422 | 0.52 | 0.8402 |  |  |
| BepiPred | VGVTYGYA | 6-15 |  | 1.3662 |  |  |
|  | EDAAHCIHAEWDTGLN | 277-297 |  | 0.7190 |  |  |
|  | AYTASDTAETTNVQGWVCVLQVTDTHSAEAAVVVSVSAG | 308-346 |  | 0.5431 |  |  |
|  | QTTSAGESADPVTTTVENYGGNTQVQRRKHTD | 359-390 |  | 0.6200 |  |  |
| Ellipro | QTTSAGESADPVTTTVENYGGNTQVQRRK | 359-387 | 0.711 | 0.5828 |  |  |
|  | GPTDHKARYMVAYVPPGVEVGQPPRTPEDAAHCIHAEWDTGLNS | 250-293 | 0.707 | 0.5461 |  |  |
|  | GWVCVLQVTDTHSAEA | 322-337 | 0.665 | 0.9015 |  |  |

Table (S3) B-cell Prediction of ACP2 Protein Over the ACP1 Protein

| Sequence | Position | Score | Antigenicity | Allergenicity | Toxicity |
| --- | --- | --- | --- | --- | --- |
| VIMIRETRKRQQMVDD | 650-665 | 0.90 | 0.8773 | Non-allergen | Non-toxin |
| TVGFRERTLPGQKADD | 698-713 | 0.82 | 1.4218 |  |  |
| EFEIKVKGQDMLSDAA | 801-816 | 0.67 | 1.2454 |  |  |
| CVSRSMLLKMKAHIDP | 923-938 | 0.93 | 0.5032 |  |  |

Table (S4) MHCI Prediction of the ACP1 Protein Using NetMHCpan Server with BoLA-1:01901 and BoLA-2:00801 Egyptian Alleles

| Sequence | Position | Score | Antigenicity | Allergenicity | Toxicity |
| --- | --- | --- | --- | --- | --- |
| VTYGYATAE | 8-16 | 0.004172 | 1.1129 | Non-allergen | Non-toxin |
| PNTSGLETR | 22-30 | 0.004680 | 1.2409 |  |  |
| LETRVVQAE | 27-35 | 0.002664 | 0.6101 |  |  |
| GLETRVVQA | 26-34 | 0.002065 | 0.7309 |  |  |
| GVYGKLTDS | 65-73 | 0.000589 | 0.9381 |  |  |
| LCSLKDREE | 105-113 | 0.000589 | 1.9884 |  |  |
| DREEYQLTL | 110-118 | 0.000125 | 0.9155 |  |  |
| REEYQLTLF | 111-119 | 0.000628 | 0.7912 |  |  |
| ITVPYLGVN | 134-142 | 0.008753 | 0.5078 |  |  |
| HKPWTLVVM | 150-158 | 0.000247 | 0.8742 |  |  |
| PWTLVVMVV | 152-160 | 0.000660 | 0.9871 |  |  |
| VMVVTPLTV | 157-165 | 0.002276 | 0.5822 |  |  |
| MVVTPLTVV | 158-166 | 0.003471 | 0.7777 |  |  |
| VTPLTVVYN | 160-168 | 0.004862 | 0.8522 |  |  |
| LDVAEACPT | 182-190 | 0.000598 | 0.7551 |  |  |
| FLCFDDGKP | 191-199 | 0.000561 | 1.5476 |  |  |
| VLATFDVSL | 210-218 | 0.005373 | 0.5885 |  |  |
| FDVSLAAKH | 214-222 | 0.005034 | 1.6373 |  |  |
| YSGTINLHF | 238-246 | 0.006813 | 1.1831 |  |  |
| GTINLHFMF | 240-248 | 0.011283 | 1.7291 |  |  |
| LHFMFTGPT | 244-252 | 0.008377 | 1.2152 |  |  |
| YVPPGVEVG | 262-270 | 0.003879 | 1.4110 |  |  |
| VEVGQPPRT | 267-275 | 0.002399 | 0.8650 |  |  |
| LNSSFTFSI | 291-299 | 0.011891 | 1.0552 |  |  |
| FTFSIPYIS | 295-303 | 0.004532 | 0.6611 |  |  |
| AEAAVVVSV | 335-343 | 0.003933 | 0.6416 |  |  |
| PDLEFRFPI | 347-355 | 0.000844 | 1.5698 |  |  |
| IDPVQTTSA | 355-363 | 0.005552 | 1.3246 |  |  |
| PVQTTSAGE | 357-365 | 0.015532 | 0.7460 |  |  |
| VQTTSAGES | 358-366 | 0.031050 | 0.7614 |  |  |
| GGNTQVQRR | 378-386 | 0.009000 | 0.5022 |  |  |
| NTQVQRRKH | 380-388 | 0.002818 | 0.7756 |  |  |
| VQRRKHTDV | 383-391 | 0.004162 | 1.3148 |  |  |
| TDVAFILDR | 389-397 | 0.009951 | 0.9245 |  |  |
| LMQTREHAL | 412-420 | 0.025331 | 0.8762 |  |  |
| REHALVGAL | 416-424 | 0.003282 | 1.0304 |  |  |
| NRLTWVPNG | 444-452 | 0.001909 | 1.3167 |  |  |
| LTWVPNGAP | 446-454 | 0.010306 | 1.0692 |  |  |
| LLNTTNPTA | 458-466 | 0.001114 | 0.6104 |  |  |
| LNTTNPTAY | 459-467 | 0.006712 | 0.6356 |  |  |
| TTNPTAYHK | 461-469 | 0.003007 | 0.7697 |  |  |
| LALPYTAPH | 475-483 | 0.015534 | 0.5062 |  |  |
| GDLAALAAK | 506-514 | 0.023932 | 1.0053 |  |  |
| AALAAKYAD | 509-517 | 0.009842 | 1.3949 |  |  |
| LPPSFNYGA | 522-530 | 0.000182 | 1.0790 |  |  |
| FNYGAITAT | 526-534 | 0.001274 | 1.6442 |  |  |
| VELLYRMKR | 537-545 | 0.248036 | 1.1280 |  |  |
| LYRMKRAEL | 540-548 | 0.008790 | 0.5189 |  |  |
| MKRAELYCP | 543-551 | 0.001512 | 1.5664 |  |  |
| LLAAYKHTD | 554-562 | 0.013209 | 0.9343 |  |  |
| KQKIIAPEK | 566-574 | 0.005777 | 0.6168 |  |  |

Table (S5) MHCI Prediction of the ACP2 Protein more than the ACP1 Protein Using NetMHCpan Server with BoLA-1:01901 and BoLA-2:00801 Egyptian Alleles

| Sequence | Position | Score | Antigenicity | Allergenicity | Toxicity |
| --- | --- | --- | --- | --- | --- |
| PSQKSVLYF | 581-589 | 0.012447 | 0.6291 | Non-allergen | Non-toxin |
| GQHEAAIEF | 594-609 | 0.134833 | 0.8012 |  |  |
| SLKEELRPL | 610-618 | 0.121794 | 1.2889 |  |  |
| FEIVALCLT | 636-644 | 0.044914 | 2.0318 |  |  |
| ETRKRQQMV | 655-670 | 0.015593 | 1.0678 |  |  |
| TLDEAEKNP | 682-697 | 0.000808 | 0.5105 |  |  |
| AICCATGVF | 759-767 | 0.006306 | 0.6044 |  |  |
| ITDRDYRVF | 792-800 | 0.107538 | 1.0280 |  |  |
| MVLHRGNRV | 818-826 | 0.012147 | 0.6275 |  |  |
| ARMKKGTPV | 837-852 | 0.011085 | 0.6828 |  |  |
| TYKDIVVCM | 865-873 | 0.005912 | 1.0376 |  |  |
| DTMPGLFAY | 876-884 | 0.212526 | 0.9710 |  |  |
| YRAATKAGY | 884-892 | 0.018954 | 0.8238 |  |  |
| SMLLKMKAH | 927-935 | 0.014347 | 0.6908 |  |  |
| AHIDPEPHH | 934-949 | 0.009367 | 1.8094 |  |  |
| YDKIMLDGR | 777-785 | 0.001008 | 0.5249 |  |  |
| FAYRAATKA | 878-892 | 0.019054 | 0.8314 |  |  |

Table (S6) MHCII Prediction of the Two Target Proteins Using MHCII-NP and NetMHCpanII-4.3 Servers with BoLA-DRB3*020:02 Egyptian Allele

| Server | Sequence | Position | Score | Antigenicity | Allergenicity | Toxicity |
| --- | --- | --- | --- | --- | --- | --- |
| MHCII-NP | VPPGVEVGQPPRTPE | 263-277 |  | 0.7120 | Non-allergen | Non-toxin |
| NetMHCpanII - 4.3 | RGDLAALAAKYADTN | 505-519 | 0.9864 | 0.8463 |  |  |
|  | EYQLTLFPHQFINPR | 113-127 | 0.6964 | 0.6128 |  |  |
|  | GTINLHFMFTGPTDH | 240-254 | 0.3983 | 1.0087 |  |  |
|  | DLAALAAKYADTNHT | 507-521 | 0.3866 | 1.0607 |  |  |
|  | KARYMVAYVPPGVEV | 255-269 | 0.2321 | 0.6839 |  |  |
|  | INPRTNMTAHITVPY | 125-138 | 0.0438 | 0.7090 |  |  |
|  | VVMVVTPLTVVYNPP | 156-170 | 0.2850 | 0.5183 |  |  |
|  | TKPVELLYRMKRAEL | 534-548 | 0.0510 | 0.9744 |  |  |
|  | PPSFNYGAITATKPV | 523-537 | 0.1260 | 1.0512 |  |  |
|  | AADYAYTASDTAETT | 304-318 | 0.1848 | 0.7460 |  |  |
|  | SAADYAYTASDTAET | 303-317 | 0.1611 | 0.6789 |  |  |
|  | GDRVLATFDVSLAAK | 207-221 | 0.0886 | 0.6689 |  |  |
|  | PWTLVVMVVTPLTVV | 152-166 | 0.1024 | 0.8550 |  |  |
|  | KRHKPWTLVVMVVTP | 148-162 | 0.0341 | 0.9753 |  |  |
|  | TFSIPYISAADYAYT | 296-310 | 0.0703 | 0.8405 |  |  |
|  | ISAADYAYTASDTAE | 302-316 | 0.1052 | 0.8278 |  |  |
|  | TYYFCDLEIAVVHDG | 429-443 | 0.0853 | 0.5637 |  |  |
|  | GVYGKLTDSYAYMRN | 65-79 | 0.0916 | 0.5267 |  |  |
|  | YYTQYSGTINLHFMF | 234-248 | 0.0437 | 0.9930 |  |  |
|  | PLLAAYKHTDRRHKQ | 553-567 | 0.0474 | 0.6522 |  |  |
|  | QTREHALVGALLRAA | 414-428 | 0.0571 | 0.6909 |  |  |
|  | AAVVVSVSAGPDLEF | 337-351 | 0.0586 | 1.1572 |  |  |
|  | KHTDVAFILDRFVKV | 387-401 | 0.0306 | 0.5004 |  |  |
|  | TNMTAHITVPYLGVN | 73-87 | 0.0512 | 0.7178 |  |  |
|  | NNYPGRFTNLLDVAE | 172-186 | 0.0486 | 0.8421 |  |  |
|  | GNRLTWVPNGAPETA | 443-457 | 0.0415 | 0.6366 |  |  |
|  | AQYYTQYSGTINLHF | 232-246 | 0.0431 | 0.7416 |  |  |
|  | LATFDVSLAAKHMSN | 211-225 | 0.0324 | 0.8393 |  |  |
|  | NRLTWVPNGAPETAL | 444-458 | 0.0300 | 0.6442 |  |  |
|  | FRFPIDPVQTTSAGE | 351-365 | 0.0099 | 0.6137 |  |  |
|  | DPVQTTSAGESADPV | 356-370 | 0.0020 | 0.7680 |  |  |
|  | AAHCIHAEWDTGLNS | 279-293 | 0.0008 | 0.6380 |  |  |

Table (S7) conserved epitopes between the different serotypes

| Epitope | VP1_A | VP1_O | VP1_SAT2 | All serotypes | VP2, VP3, 3A, 3C |
| --- | --- | --- | --- | --- | --- |
| Epitope 1 | 0 | 2 | 0 | 2 | 1 |
| Epitope 2 | 0 | 0 | 0 | 0 | 0 |
| Epitope 3 | 0 | 0 | 0 | 0 | 0 |
| Epitope 4 | 0 | 0 | 0 | 0 | 0 |
| Epitope 5 | 0 | 0 | 0 | 0 | 0 |
| Epitope 6 | 0 | 0 | 0 | 0 | 0 |
| Epitope 7 | 0 | 0 | 0 | 0 | 0 |
| Epitope 8 | 0 | 3 | 0 | 3 | 1 |
| Epitope 9 | 0 | 0 | 0 | 0 | 0 |
| Epitope 10 | 0 | 0 | 5 | 5 | 1 |
| Epitope 11 | 0 | 0 | 4 | 4 | 1 |
| Epitope 12 | 0 | 0 | 0 | 0 | 0 |
| Epitope 13 | 0 | 0 | 0 | 0 | 0 |
| Epitope 14 | 0 | 0 | 0 | 0 | 0 |
| Epitope 15 | 0 | 0 | 0 | 0 | 0 |
| Epitope 16 | 0 | 0 | 0 | 0 | 1 |
| Epitope 17 | 0 | 0 | 0 | 0 | 0 |
| Epitope 18 | 1 | 1 | 4 | 6 | 3 |
| Epitope 19 | 0 | 1 | 0 | 1 | 2 |
| Epitope 20 | 0 | 2 | 0 | 2 | 1 |
| Epitope 21 | 1 | 2 | 5 | 8 | 3 |
| Epitope 22 | 0 | 0 | 0 | 0 | 0 |
| Epitope 23 | 0 | 0 | 1 | 1 | 0 |
| Epitope 24 | 1 | 0 | 0 | 1 | 1 |
| Epitope 25 | 1 | 2 | 0 | 3 | 2 |
| Epitope 26 | 0 | 0 | 0 | 0 | 0 |
| Epitope 27 | 1 | 2 | 1 | 4 | 3 |
| Epitope 28 | 0 | 0 | 0 | 0 | 1 |
| Epitope 29 | 1 | 0 | 0 | 1 | 1 |
| Epitope 30 | 1 | 3 | 0 | 4 | 2 |
| Epitope 31 | 0 | 0 | 0 | 0 | 0 |
| Epitope 32 | 0 | 0 | 0 | 0 | 0 |
| Epitope 33 | 0 | 0 | 4 | 4 | 1 |
| Epitope 34 | 0 | 0 | 0 | 0 | 0 |
| Epitope 35 | 0 | 0 | 0 | 0 | 0 |
| Epitope 36 | 0 | 0 | 0 | 0 | 0 |
| Epitope 37 | 0 | 0 | 0 | 0 | 0 |
| Epitope 38 | 0 | 0 | 27 | 27 | 0 |
| Epitope 39 | 0 | 0 | 0 | 0 | 0 |
| Epitope 40 | 58 | 0 | 50 | 108 | 2 |
| Epitope 41 | 1 | 1 | 5 | 7 | 3 |
| Epitope 42 | 1 | 1 | 5 | 7 | 3 |
| Epitope 43 | 0 | 0 | 0 | 0 | 1 |
| Epitope 44 | 1 | 1 | 0 | 2 | 2 |
| Epitope 45 | 0 | 0 | 0 | 0 | 1 |
| Epitope 46 | 1 | 1 | 4 | 6 | 3 |
| Epitope 47 | 1 | 1 | 4 | 6 | 3 |
| Epitope 48 | 0 | 0 | 0 | 0 | 0 |
| Epitope 49 | 1 | 1 | 4 | 6 | 3 |
| Epitope 50 | 1 | 1 | 0 | 2 | 2 |
| Epitope 51 | 1 | 1 | 4 | 6 | 3 |
| Epitope 52 | 1 | 1 | 5 | 7 | 3 |
| Epitope 53 | 0 | 1 | 0 | 1 | 2 |
| Epitope 54 | 1 | 1 | 0 | 2 | 2 |
| Epitope 55 | 0 | 1 | 4 | 5 | 2 |
| Epitope 56 | 1 | 0 | 0 | 1 | 1 |
| Epitope 57 | 0 | 0 | 0 | 0 | 0 |
| Epitope 58 | 0 | 0 | 0 | 0 | 0 |
| Epitope 59 | 0 | 0 | 0 | 0 | 1 |
| Epitope 60 | 0 | 0 | 0 | 0 | 0 |
| Epitope 61 | 1 | 3 | 0 | 4 | 1 |
| Epitope 62 | 0 | 0 | 0 | 0 | 0 |
| Epitope 63 | 0 | 0 | 0 | 0 | 0 |
| Epitope 64 | 1 | 3 | 0 | 4 | 2 |
| Epitope 65 | 0 | 0 | 0 | 0 | 0 |
| Epitope 66 | 0 | 0 | 0 | 0 | 0 |
| Epitope 67 | 0 | 0 | 5 | 5 | 1 |
| Epitope 68 | 0 | 0 | 0 | 0 | 0 |
| Epitope 69 | 0 | 0 | 0 | 0 | 0 |
| Epitope 70 | 0 | 0 | 0 | 0 | 0 |
| Epitope 71 | 0 | 0 | 0 | 0 | 0 |
| Epitope 72 | 0 | 0 | 0 | 0 | 0 |
| Epitope 73 | 0 | 0 | 0 | 0 | 0 |
| Epitope 74 | 1 | 1 | 1 | 3 | 2 |
| Epitope 75 | 0 | 1 | 0 | 1 | 2 |

Table (S8) B-cell Discontinuous epitopes of Version 1 of vaccine constructs

| No. | Residues | Number of residues | Score |
| --- | --- | --- | --- |
| 1 | B:H126, B:T127, B:D128, B:V129, B:A130, B:A131, B:Y132, B:R133, B:H135 | 9 | 0.91 |
| 2 | B:H338, B:H339, B:H340, B:H341, B:E342, B:A343, B:A344, B:A345, B:K346, B:A347, B:K348, B:F349, B:V350, B:A351, B:A352, B:W353, B:T354, B:L355, B:K356, B:A357, B:A358, B:A359, B:K360, B:K361, B:A362, B:P363, B:P364, B:H365, B:A366, B:L367, B:S368 | 31 | 0.879 |
| 3 | B:Y268, B:G269, B:Y270, B:A271, B:K272 | 5 | 0.795 |
| 4 | B:P13, B:N14, B:T15, B:S16, B:G17, B:L18, B:E19, B:T20, B:R21, B:C39 | 10 | 0.755 |
| 5 | B:Y72, B:Y73, B:V74, B:P75, B:P76, B:G77, B:V78, B:E79, B:V80, B:G81, B:A82 | 11 | 0.704 |
| 6 | B:A221, B:K222, B:Y223, B:A224, B:D225, B:T226, B:N227, B:G228, B:P229, B:G230, B:P231, B:G232, B:P233, B:P234, B:S235, B:F236 | 16 | 0.697 |
| 7 | B:A243, B:T244, B:K245, B:P246, B:V247, B:E248, B:L249, B:L250, B:R252, B:M253, B:K254, B:R255, B:A256, B:E257, B:L258, B:G259, B:P260, B:G261, B:K273, B:S274, B:V275, B:S276, B:A277, B:G278, B:P279, B:D280, B:L281, B:E282, B:F283, B:R284, B:F285, B:P286, B:I287, B:D288, B:P289, B:K290 | 36 | 0.688 |
| 8 | B:Y108, B:I109, B:D110, B:P111, B:V112, B:Q113, B:T114, B:T115, B:S116, B:A117, B:A118, B:A119, B:Y120, B:V121, B:Q122, B:R123 | 16 | 0.685 |
| 9 | B:L306, B:D307, B:K308, B:K309, B:D310, B:L311, B:A312, B:A313, B:L314, B:A315, B:A316, B:K317, B:Y318, B:A319, B:D320, B:T321, B:N322 | 17 | 0.639 |
| 10 | B:Y5, B:A6, B:T7, B:A8, B:E9, B:A10, B:A11, B:Y12 | 8 | 0.633 |
| 11 | B:V150, B:P151, B:N152, B:G153, B:A154, B:A155, B:A157, B:A158, B:L159, B:A160, B:A161, B:K162, B:Y163, B:A164, B:D165, B:A166 | 16 | 0.625 |
| 12 | B:H323, B:T324, B:L325 | 3 | 0.599 |
| 13 | B:A136, B:L137, B:V138, B:G139, B:A140, B:L141, B:A142, B:A143, B:N145, B:R146 | 10 | 0.597 |

Table (S9) B-cell Discontinuous epitopes of Version 2 of vaccine constructs

| No. | Residues | Number of residues | Score |
| --- | --- | --- | --- |
| 1 | B:T634, B:L635, B:K636, B:A637, B:A638, B:A639, B:K640, B:K641, B:A642, B:P643, B:P644, B:H645, B:A646, B:L647, B:S648 | 15 | 0.976 |
| 2 | B:K355, B:A356, B:H357, B:A358, B:A359, B:Y360, B:A361, B:H362, B:I363, B:D364, B:P365, B:E366, B:P367 | 13 | 0.923 |
| 3 | B:N145, B:R146, B:L147, B:T148 | 4 | 0.919 |
| 4 | B:K610, B:A611, B:H612, B:I613, B:D614, B:P615, B:H616, B:H617 | 8 | 0.916 |
| 5 | B:W149, B:V150, B:P151, B:N152, B:G153, B:A154, B:A155, B:Y156 | 8 | 0.903 |
| 6 | B:K342, B:A343, B:G344, B:Y345, B:A346, B:A347, B:Y348, B:S349, B:M350, B:L351, B:L352, B:K353, B:M354 | 13 | 0.891 |
| 7 | B:F629, B:V630, B:A631, B:A632, B:W633 | 5 | 0.877 |
| 8 | B:R603, B:S604, B:M605, B:L606, B:L607 | 5 | 0.871 |
| 9 | B:R183, B:A184, B:E185, B:L186, B:Y187 | 5 | 0.856 |
| 10 | B:A71, B:Y72, B:Y73, B:V74, B:P75, B:P76, B:G77, B:V78, B:E79, B:V80, B:G81, B:A82, B:A83, B:Y84, B:L85, B:N86, B:S87, B:S88, B:F89 | 19 | 0.831 |
| 11 | B:A339, B:A340, B:T341 | 3 | 0.807 |
| 12 | B:R21, B:A22, B:A23, B:Y24 | 4 | 0.802 |
| 13 | B:F91, B:S92, B:I93, B:A94, B:A95, B:Y96, B:P97, B:D98, B:L99, B:E100, B:F101 | 11 | 0.77 |
| 14 | B:H135, B:A136, B:L137, B:V138, B:G139, B:A140, B:L141, B:A142, B:A143, B:Y144 | 10 | 0.763 |
| 15 | B:Y171, B:G172, B:A173, B:I174, B:T175, B:A176, B:T177, B:A178, B:A179, B:Y180, B:M181, B:K182 | 12 | 0.754 |
| 16 | B:A157, B:A158, B:L159, B:A160, B:A161, B:K162, B:Y163 | 7 | 0.74 |
| 17 | B:E220, B:E221, B:L222, B:P224, B:L225, B:A226, B:A227, B:Y228, B:F229, B:E230, B:I231, B:V232, B:A233, B:L234, B:C235, B:L236, B:T237, B:A238, B:A239, B:Y240 | 20 | 0.733 |
| 18 | B:A462, B:A463, B:T464, B:K465, B:A466, B:G467, B:P468, B:G469, B:P470, B:G471, B:V472, B:G473, B:V474, B:T475 | 14 | 0.705 |
| 19 | B:H368, B:H369, B:A370, B:A371, B:Y372 | 5 | 0.702 |
| 20 | B:C600, B:V601, B:S602 | 3 | 0.685 |
| 21 | B:L25, B:C26, B:S27, B:L28, B:K29, B:D30 | 6 | 0.678 |
| 22 | B:G205, B:Q206, B:E208, B:A209, B:A210, B:I211, B:E212, B:F213, B:A214, B:A215 | 10 | 0.658 |
| 23 | B:K315, B:D316, B:I317, B:V318, B:V319, B:C320, B:M321, B:A322, B:A323, B:Y324, B:D325, B:T326, B:M327, B:P328, B:G329, B:L330, B:F331, B:A332, B:Y333, B:A334, B:A335, B:Y336 | 22 | 0.655 |
| 24 | B:C188, B:P189, B:A190, B:A191, B:Y192, B:P193, B:S194, B:Q195, B:K196, B:S197, B:V198 | 11 | 0.655 |
| 25 | B:A164, B:D165, B:A166, B:A167, B:Y168, B:F169, B:N170 | 7 | 0.652 |
| 26 | B:Y216, B:S217, B:L218, B:K219 | 4 | 0.642 |
| 27 | B:Q247, B:M248, B:V249, B:A250, B:A251, B:Y252, B:T253, B:L254 | 8 | 0.628 |
| 28 | B:P13, B:N14, B:T15, B:S16, B:G17, B:L18 | 6 | 0.621 |
| 29 | B:V284, B:F285, B:A286, B:A287, B:Y288, B:M289, B:V290 | 7 | 0.608 |
| 30 | B:R280, B:D281, B:Y282, B:R283 | 4 | 0.602 |
| 31 | B:H618, B:H619, B:H620, B:H621, B:E622, B:A623, B:A624, B:A625, B:K626, B:A627, B:K628 | 11 | 0.59 |
| 32 | B:A373, B:A374, B:V375, B:V376, B:V377, B:S378 | 6 | 0.575 |
| 33 | B:G455, B:P456, B:G457, B:F458, B:A459, B:Y460, B:R461 | 7 | 0.552 |
| 34 | B:F103, B:P104, B:I105, B:A106, B:A107, B:Y108, B:I109, B:D110, B:P111, B:V112, B:Q113, B:T114 | 12 | 0.538 |
| 35 | B:H57, B:A58, B:A59, B:Y60, B:G61, B:T62, B:I63 | 7 | 0.514 |
| 36 | B:Y200, B:F201, B:A202, B:A203, B:Y204 | 5 | 0.506 |

Table (S10) B-cell Discontinuous epitopes of Version 3 of vaccine constructs

| No. | Residues | Number of residues | Score |
| --- | --- | --- | --- |
| 1 | B:A493, B:A494, B:A495, B:K496, B:K497, B:A498, B:P499, B:P500, B:H501, B:A502, B:L503, B:S504 | 12 | 0.974 |
| 2 | B:K357, B:A358, B:G359, B:P360, B:G361, B:P362, B:G363, B:V364, B:G365, B:V366, B:T367 | 11 | 0.948 |
| 3 | B:Y3, B:G4, B:Y5, B:A6, B:T7, B:A8, B:E9, B:A10, B:A11, B:Y12, B:P13, B:N14, B:T15, B:S16, B:G17, B:L18 | 16 | 0.885 |
| 4 | B:H126, B:T127, B:D128, B:V129, B:A130, B:A131, B:Y132, B:R133, B:E134 | 9 | 0.867 |
| 5 | B:A479, B:A480, B:A481, B:K482, B:A483, B:K484, B:F485, B:V486, B:A487, B:A488, B:W489, B:T490, B:L491, B:K492 | 14 | 0.863 |
| 6 | B:T445, B:L446, B:P447, B:G448, B:Q449, B:K450, B:A451, B:D452, B:D453 | 9 | 0.852 |
| 7 | B:G274, B:P275, B:D276, B:L277, B:E278, B:F279, B:G280, B:P281, B:G282, B:P283, B:G284, B:R285, B:G286, B:D287, B:L288, B:A289, B:A290, B:L291, B:A292, B:A293, B:K294, B:Y295, B:A296, B:D297, B:T298, B:N299, B:G300, B:P301, B:G302, B:P303, B:G304 | 31 | 0.744 |
| 8 | B:E19, B:T20, B:R21, B:A71, B:Y72, B:V74, B:P75, B:P76, B:G77, B:V78, B:E79, B:V80, B:G81, B:A82, B:A83, B:L85 | 16 | 0.707 |
| 9 | B:G345, B:P346, B:G347, B:P348, B:G349, B:F350, B:A351, B:Y352, B:R353, B:A354, B:A355, B:T356 | 12 | 0.7 |
| 10 | B:I405, B:L406, B:D407, B:K408, B:K409, B:D410, B:L411, B:A412, B:A413, B:L414, B:A415, B:A416, B:K417, B:Y418, B:A419, B:D420, B:T421, B:N422, B:K455, B:F457 | 20 | 0.686 |
| 11 | B:E33, B:A34, B:A35, B:Y36 | 4 | 0.646 |
| 12 | B:K29, B:D30, B:R31, B:E32 | 4 | 0.645 |
| 13 | B:N145, B:L147, B:T148 | 3 | 0.643 |
| 14 | B:F37, B:L38, B:C39, B:F40, B:D41, B:D42, B:G43, B:K44, B:P45, B:A46 | 10 | 0.636 |
| 15 | B:L53, B:A54, B:A55, B:K56, B:H57, B:A58, B:A59 | 7 | 0.631 |
| 16 | B:A312, B:I313, B:T314, B:A315, B:T316, B:K317, B:P318, B:V319, B:E320, B:P386, B:I387, B:D388, B:P389, B:K390 | 14 | 0.616 |
| 17 | B:W149, B:V150, B:P151, B:N152, B:G153, B:A154, B:A155, B:A157, B:A158, B:L159, B:A160, B:A161, B:K162, B:Y163, B:A164, B:D165, B:A166 | 17 | 0.593 |
| 18 | B:D110, B:P111, B:V112, B:Q113, B:T114, B:T115, B:S116, B:A117, B:A118, B:A119, B:Y120 | 11 | 0.585 |
| 19 | B:T435, B:K436, B:K437 | 3 | 0.542 |
| 20 | B:K391, B:T392, B:Q393, B:V394, B:Q395 | 5 | 0.538 |
| 21 | B:C188, B:P189, B:A190 | 3 | 0.536 |
| 22 | B:D342, B:G343, B:R344 | 3 | 0.536 |
| 23 | B:H423, B:T424, B:L425, B:P426, B:P427, B:S428, B:F429 | 7 | 0.529 |
| 24 | B:H135, B:A136, B:L137, B:L141 | 4 | 0.511 |
| 25 | B:Y368, B:G369, B:Y370, B:A371 | 4 | 0.511 |
| 26 | B:Y24, B:L25, B:C26 | 3 | 0.504 |

Table (S11) Disulfide Engineering of Version 1 of the constructed vaccine

| Res1 Chain | Res1 Seq # | Res1 AA | Res2 Chain | Res2 Seq # | Res2 AA | Chi3 | Energy | Sum B-Factors |
| --- | --- | --- | --- | --- | --- | --- | --- | --- |
| A | 28 | LEU | A | 31 | ARG | -115.56 | 3.72 | 0 |
| A | 61 | GLY | A | 201 | ALA | -98.96 | 3.92 | 0 |
| A | 96 | TYR | A | 99 | LEU | 71.66 | 2.87 | 0 |
| A | 151 | PRO | A | 156 | TYR | 123.92 | 5.64 | 0 |
| A | 211 | PRO | A | 215 | ASP | 109.82 | 2.82 | 0 |
| A | 211 | PRO | A | 218 | ALA | -81.55 | 5.8 | 0 |
| A | 215 | ASP | A | 218 | ALA | 106.27 | 2.87 | 0 |
| A | 226 | THR | A | 230 | GLY | -93.75 | 2.05 | 0 |
| A | 276 | SER | A | 282 | GLU | 115.68 | 6.5 | 0 |
| A | 306 | LEU | A | 310 | ASP | 113.33 | 3.22 | 0 |
| A | 362 | ALA | A | 366 | ALA | 82.15 | 5.38 | 0 |

Table (S12) Disulfide Engineering of Version 2 of the constructed vaccine

| Res1 Chain | Res1 Seq # | Res1 AA | Res2 Chain | Res2 Seq # | Res2 AA | Chi3 | Energy | Sum B-Factors |
| --- | --- | --- | --- | --- | --- | --- | --- | --- |
| A | 28 | LEU | A | 31 | ARG | -115.56 | 3.72 | 0 |
| A | 61 | GLY | A | 201 | ALA | -98.96 | 3.92 | 0 |
| A | 96 | TYR | A | 99 | LEU | 71.66 | 2.87 | 0 |
| A | 151 | PRO | A | 156 | TYR | 123.92 | 5.64 | 0 |
| A | 211 | PRO | A | 215 | ASP | 109.82 | 2.82 | 0 |
| A | 211 | PRO | A | 218 | ALA | -81.55 | 5.8 | 0 |
| A | 215 | ASP | A | 218 | ALA | 106.27 | 2.87 | 0 |
| A | 226 | THR | A | 230 | GLY | -93.75 | 2.05 | 0 |
| A | 276 | SER | A | 282 | GLU | 115.68 | 6.5 | 0 |
| A | 306 | LEU | A | 310 | ASP | 113.33 | 3.22 | 0 |
| A | 362 | ALA | A | 366 | ALA | 82.15 | 5.38 | 0 |

Table (S13) Disulfide Engineering of Version 3 of the constructed vaccine

| Res1 Chain | Res1 Seq # | Res1 AA | Res2 Chain | Res2 Seq # | Res2 AA | Chi3 | Energy | Sum B-Factors |
| --- | --- | --- | --- | --- | --- | --- | --- | --- |
| A | 96 | TYR | A | 99 | LEU | -94.95 | 4.2 | 0 |
| A | 188 | CYS | A | 191 | ALA | 126.82 | 4.9 | 0 |
| A | 283 | PRO | A | 287 | ASP | -80.17 | 5.01 | 0 |
| A | 283 | PRO | A | 290 | ALA | -87.9 | 4.37 | 0 |
| A | 332 | PRO | A | 336 | TYR | 118.51 | 6.4 | 0 |
| A | 407 | ASP | A | 410 | ASP | 118.64 | 3.53 | 0 |


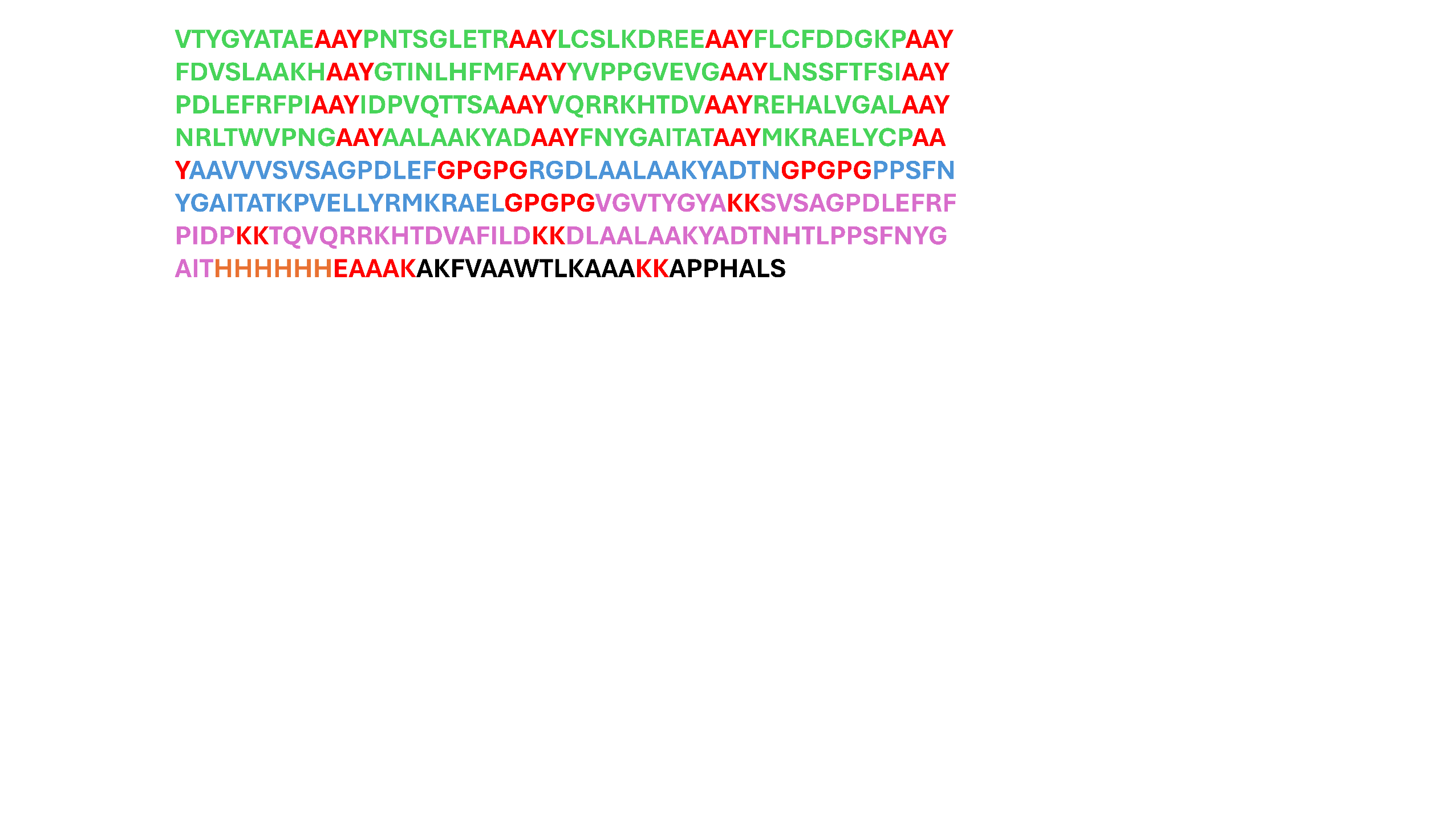


Fig. S1: The designed version 1 vaccine candidate is sequenced as CTL-AAY-THL-GPGPG-B cell-KK-6H tag-EAAAK-adjuvant-KK-adjuvant. CTL epitopes are shown in green, HTL epitopes in blue, B-cell epitopes in purple, and the PADRE sequence in black, while all linkers are highlighted in red.


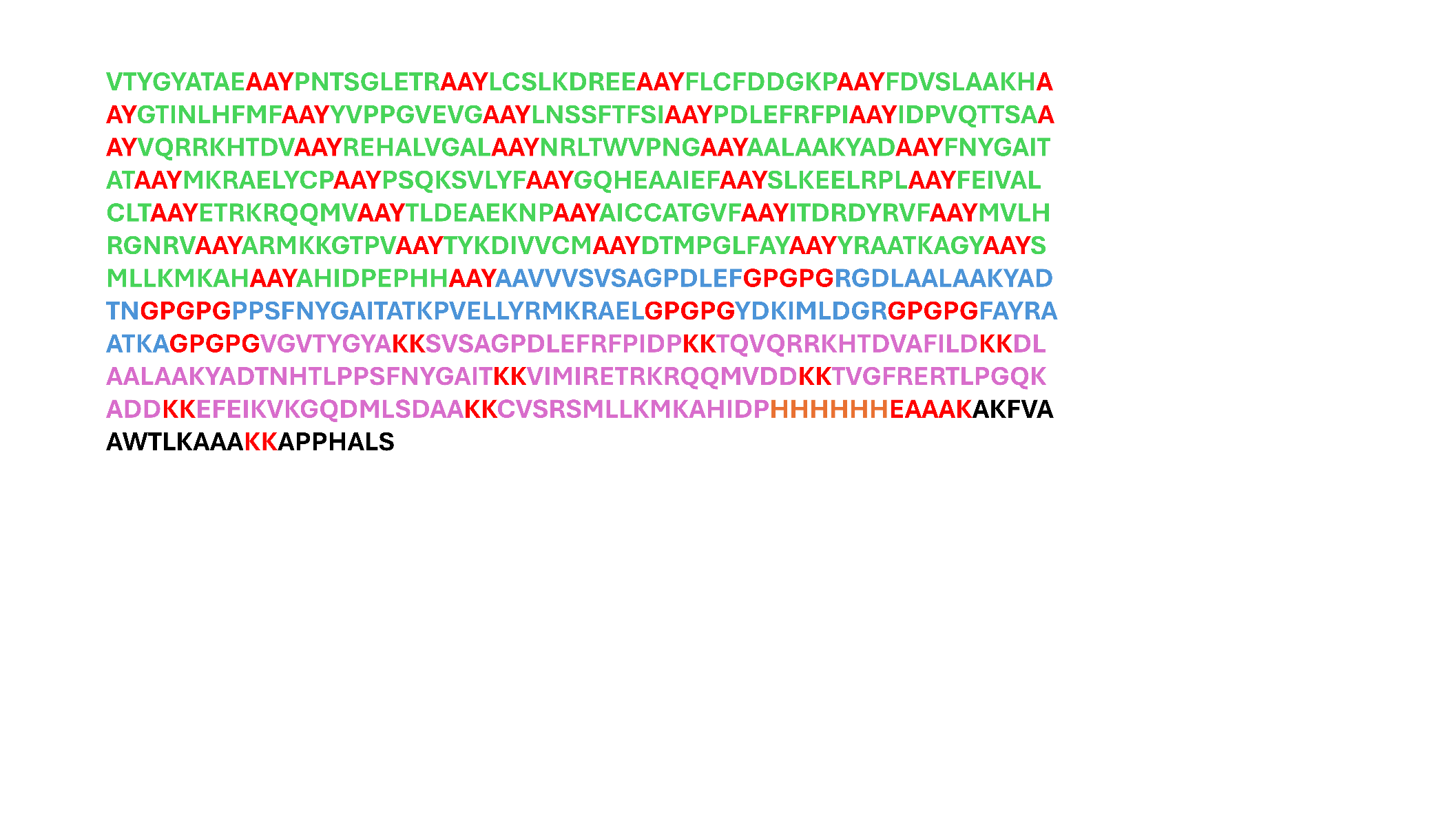


Fig. S2: The designed version 2 vaccine candidate is sequenced as CTL-AAY-THL-GPGPG-B cell-KK-6H tag-EAAAK-adjuvant-KK-adjuvant. CTL epitopes are shown in green, HTL epitopes in blue, B-cell epitopes in purple, and the PADRE sequence in black, while all linkers are highlighted in red.


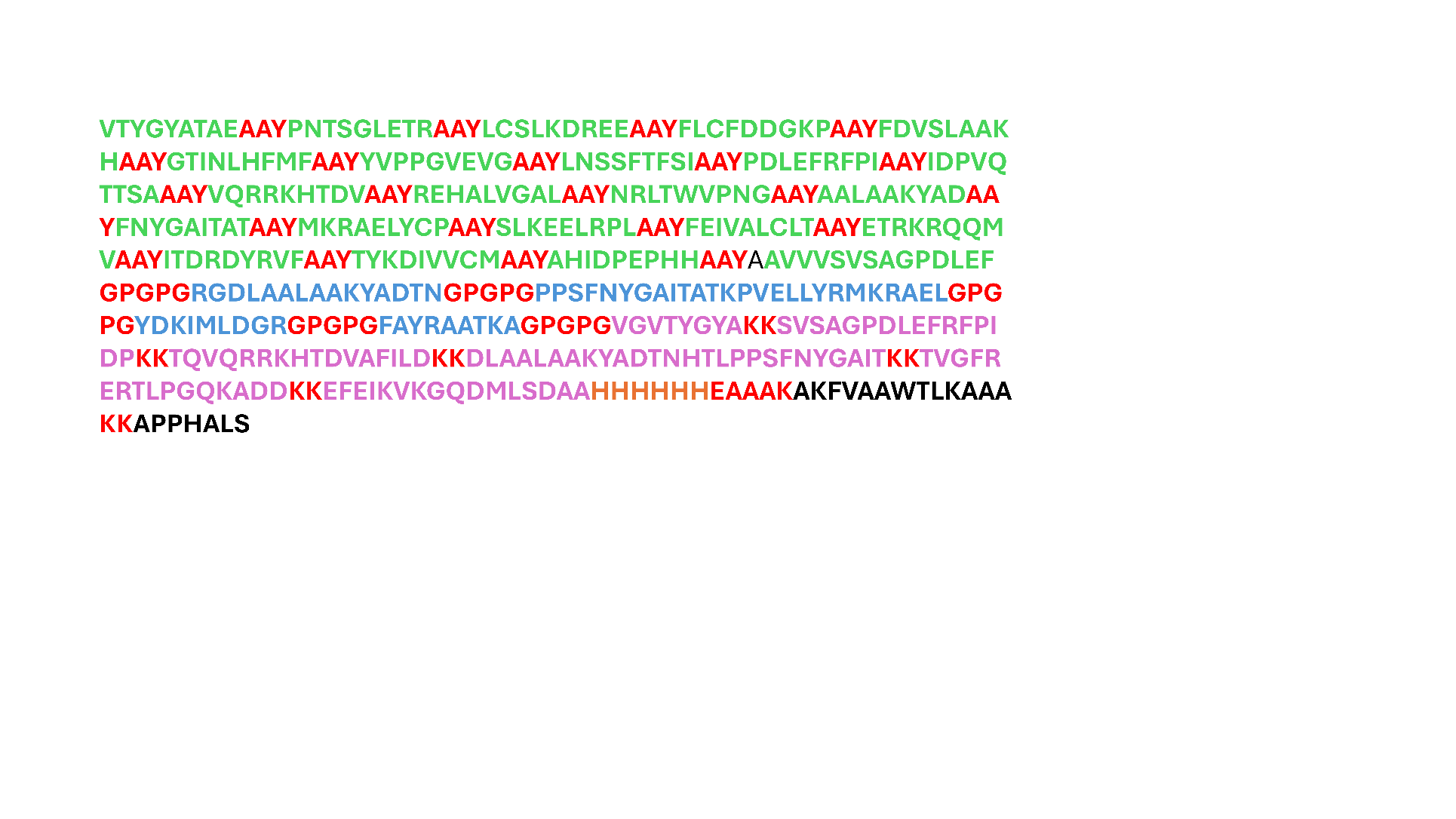


Fig. S3: The designed version 3 vaccine candidate is sequenced as CTL-AAY-THL-GPGPG-B cell-KK-6H tag-EAAAK-adjuvant-KK-adjuvant. CTL epitopes are shown in green, HTL epitopes in blue, B-cell epitopes in purple, and the PADRE sequence in black, while all linkers are highlighted in red.
